# An essential pentatricopeptide repeat protein in the apicomplexan remnant chloroplast

**DOI:** 10.1111/cmi.13108

**Published:** 2019-09-16

**Authors:** Joanna L. Hicks, Imen Lassadi, Emma F. Carpenter, Madeleine Eno, Alexandros Vardakis, Ross F. Waller, Christopher J. Howe, R. Ellen R. Nisbet

**Affiliations:** ^1^ Department of Biochemistry University of Cambridge Cambridge UK; ^2^ Present address: Faculty of Science Waikato University Hamilton New Zealand

**Keywords:** apicomplexa, apicoplast, *Plasmodium*, plastid, RNA processing, *Toxoplasma*

## Abstract

The malaria parasite *Plasmodium* and other apicomplexans such as *Toxoplasma* evolved from photosynthetic organisms and contain an essential, remnant plastid termed the apicoplast. Transcription of the apicoplast genome is polycistronic with extensive RNA processing. Yet little is known about the mechanism of apicoplast RNA processing. In plants, chloroplast RNA processing is controlled by multiple pentatricopeptide repeat (PPR) proteins. Here, we identify the single apicoplast PPR protein, PPR1. We show that the protein is essential and that it binds to RNA motifs corresponding with previously characterized processing sites. Additionally, PPR1 shields RNA transcripts from ribonuclease degradation. This is the first characterization of a PPR protein from a nonphotosynthetic plastid.

## 1 INTRODUCTION

The malaria parasite *Plasmodium falciparum* and related apicomplexan parasites such as *Toxoplasma* evolved from photosynthetic organisms. They contain a remnant plastid known as an apicoplast (Gardner, Williamson, & Wilson, 1991; Howe, 1992; McFadden, Reith, Munholland, & Lang‐Unnasch, 1996). Although the ability to photosynthesise has been lost, the apicoplast remains essential for parasite survival. The apicoplast genome encodes 30 proteins, two rRNAs, and 25 tRNAs (Wilson et al., 1996). Primary RNA transcripts are polycistronic, and there is extensive RNA processing to produce individual tRNA, rRNA, and mRNA molecules (Nisbet, Kurniawan, Bowers, & Howe, 2016; Nisbet & McKenzie, 2016). RNA processing must be controlled by nuclear‐encoded proteins that are targeted to the organelle, because no RNA processing proteins are encoded on the apicoplast genome.

In plants, the primary agents through which the nucleus exerts control on organelle gene expression are pentatricopeptide repeat (PPR) proteins. PPR proteins are encoded in the nuclear genome and are targeted to the mitochondrion or plastid (Barkan & Small, 2014). Plants contain many hundreds of PPRs (Lurin et al., 2004). By contrast, genomes of algae and nonphotosynthetic eukaryotes encode relatively few PPR proteins (Manna, 2015; Tourasse, Choquet, & Vallon, 2013). PPR proteins are involved in all aspects of organelle RNA biology, including splicing, editing, transcript stability, and translation. They are sequence‐specific RNA‐binding proteins, containing 2–30 tandem repeats, with each repeat comprising a 35‐amino acid motif that folds into a helix‐turn‐helix structure (Manna, 2015; Prikryl, Rojas, Schuster, & Barkan, 2011). Within each repeat, RNA‐binding specificity is determined by combinations of two specific amino acid positions. This is termed the PPR code (Barkan et al., 2012; Manna, 2015; Yin et al., 2013). Plants with chloroplast PPR mutants show defects in fertility and embryo and seed development (Bryant, Lloyd, Sweeney, Myouga, & Meinke, 2011; Lurin et al., 2004; Prikryl et al., 2011; Sosso et al., 2012; Sosso et al., 2012)

Very little is known about the molecular mechanisms of posttranscriptional processing in the apicoplast. A number of nucleus‐encoded, apicoplast‐targeted proteins have been identified, which may function in RNA processing. Only one RNA‐binding protein (*Plasmodium vivax PVX_084415*) has been partially characterized, although the stability of the heterologously expressed protein was such that it was not possible to carry out functional assays, though it did bind to uridine‐rich RNA (García‐Mauriño et al., 2018). The insoluble nature of both heterologously expressed *Plasmodium* proteins (Mehlin et al., 2006) and PPR proteins (Manna, 2015; Rackham & Filipovska, 2012) has impeded characterization of their structure and function.

Here, we report the identification of a single apicoplast PPR protein. We show that this protein, designated PPR1, is localized within the apicoplast of both *P. falciparum* and *Toxoplasma gondii* and is essential. Biochemical characterisation of the *P. falciparum* PPR protein shows it binds to a specific RNA sequence and protects RNA transcripts from degradation by ribonuclease in vitro. Although the presence of a PPR protein in the apicoplast is not unexpected, the dependence of a plastid on just a single PPR protein is unique. This is the first characterization of a PPR protein from a nonphotosynthetic chloroplast and represents a leap forward in our understanding of essential events in apicoplast RNA biology.

## 2 RESULTS

### 2.1 A single apicoplast PPR protein present in both *Plasmodium* and *Toxoplasma*

Searches of the *P. falciparum* genome for genes encoding PPR proteins identified only two genes, *Pf*PPR1 (PF3D7_1406400 (PF14_0061)) and *Pf*PPR2 (PF3D7_1233300 (PFL1605W)). Both genes encode proteins with 10 PPR motifs, as predicted by TPRpred (Karpenahalli, Lupas, & Söding, 2007; Figure S1A). *Pf*PPR1 belongs to the P‐class of PPR proteins, as the repeats all comprise 35 amino acids, and the final PPR motif is situated at the C‐terminus of the protein. *Pf*PPR2 likely belongs to the PLS class, as its PPR elements are not located at its C‐terminus. Orthologues of both *Pf*PPR1 and *Pf*PPR2 were found in all *Plasmodium* species with no evidence of paralogues created by lineage‐specific gene duplications.

For a protein to be targeted to the apicoplast, it must contain both a signal peptide and a plastid‐targeting sequence. *Pf*PPR1 and *Pf*PPR2 were analysed by PlasmoAP and PlasMit for putative apicoplast and/or mitochondrial localization signals (Bender, van Dooren, Ralph, McFadden, & Schneider, 2003; Foth et al., 2003). *Pf*PPR1 analysis by PlasmoAP resulted in 3/4 positive tests for a signal peptide and 5/5 positive tests for an apicoplast targeting peptide, whereas PlasMit gave a prediction of 99% for not being mitochondrial. PlasmoAP analyses of PPR1 sequences from other *Plasmodium* species similarly predicted that most have an apicoplast localisation (Table S1), the exceptions being those encoded on genomes with a high GC content, where PlasmoAP is less accurate (Foth et al., 2003). This is consistent with an overall strong prediction that *Pf*PPR1 traffics to the apicoplast. Alignments of PPR1 show that the protein is well conserved across *Plasmodium* species with little conservation of amino acids between PPR motifs. *Pf*PPR2 was predicted to lack both a signal peptide and a mitochondrial targeting sequence so its location is unknown, but it is unlikely to be apicoplast targeted. A phylogenetic analysis showed that PPR1 and PPR2 are distinct (Figure S1B).

To test for the presence of PPRs more broadly in the Apicomplexa, we searched for homologues in *Toxoplasma* and *Cryptosporidium*. Analysis of the *Toxoplasma* genome using BLAST and TPRPred identified five PPR proteins. Only one protein (TGGT1_244050, *Tg*PPR1) contained a predicted signal peptide followed by a plastid‐targeting sequence as analysed by SignalP and iPSORT (Bannai, Tamada, Maruyama, Nakai, & Miyano, 2002; Nielsen, 2017). None of the other four proteins was predicted to include a signal peptide. This indicates that there is only one apicoplast‐targeted PPR protein in *Toxoplasma*, as is the case in *Plasmodium* spp. Both *Pf*PPR1 and *Tg*PPR1 contain 10 PPR motifs (Figure S1A). The apicomplexan *Cryptosporidium*, which has lost the apicoplast, did not contain any genes encoding PPR proteins. An alignment is shown in Figure S2.

**Figure 1:**
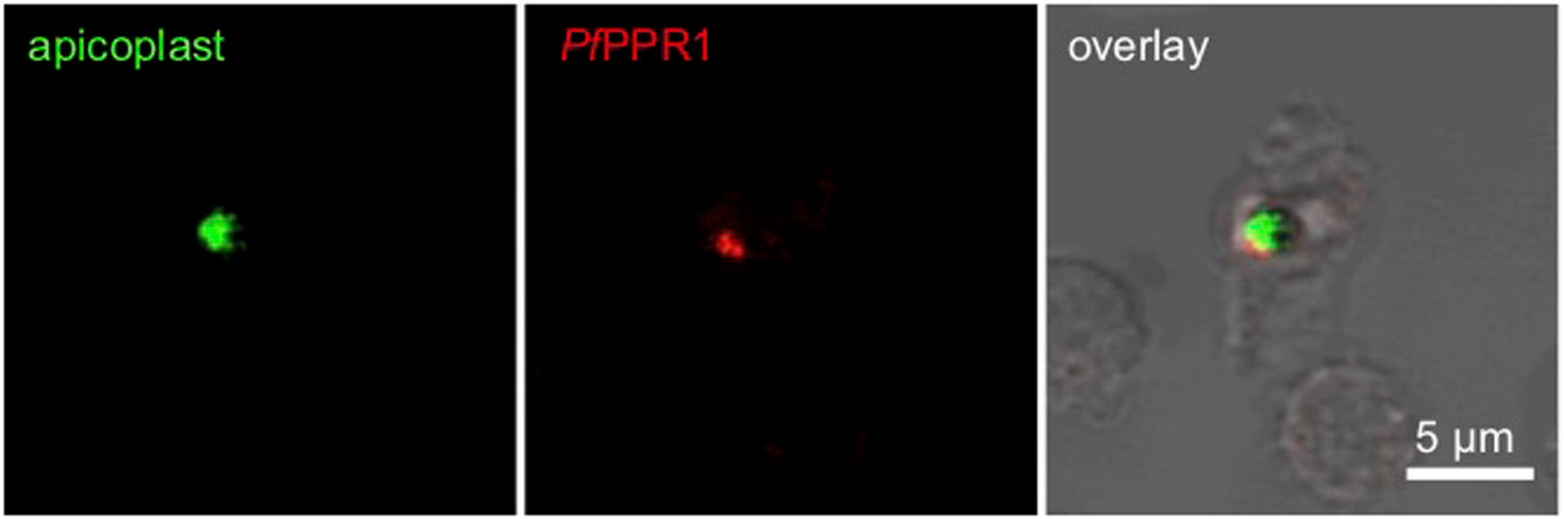
*Pf*PPR1 is localized to the *Plasmodium falciparum* 3D7 apicoplast. Immunofluorescence microscopy using *P. falciparum* D10 ACP_L_‐GFP parasites that target GFP to the apicoplast (left panel) and an antibody specific for *Pf*PPR1 with a secondary AlexaFluor‐568 antibody (middle panel) showed localization of *Pf*PPR1 to the apicoplast (indicated by overlay with bright field image in the right panel)

To test for the localization of the *Pf*PPR1 protein, we expressed recombinant *Pf*PPR1 in *Escherichia coli*, purified by IMAC and gel filtration chromatography (Figure S3) and raised polyclonal antisera in rabbits and used immunofluorescence microscopy to locate the protein. The signal from cells stained with anti‐*Pf*PPR1 colocalized with apicoplast‐located GFP in the *P. falciparum* D10‐ACP_L_ parasite line (Waller, Reed, Cowman, & McFadden, 2000; Figure 1), showing likely apicoplast localization of *Pf*PPR1. Despite extensive optimisation (Table S2), we failed to detect *Pf*PPR1 in Western blots of *P. falciparum* 3D7 lysate probed with the anti‐*Pf*PPR1 and a secondary anti‐rabbit HRP conjugate antibody (Figure S4). Western blots containing total *P. falciparum* protein showed no detectable band, presumably due to low expression of the endogenous protein. We were able to detect the recombinant protein, indicating that the antibody works.

**Figure 2:**
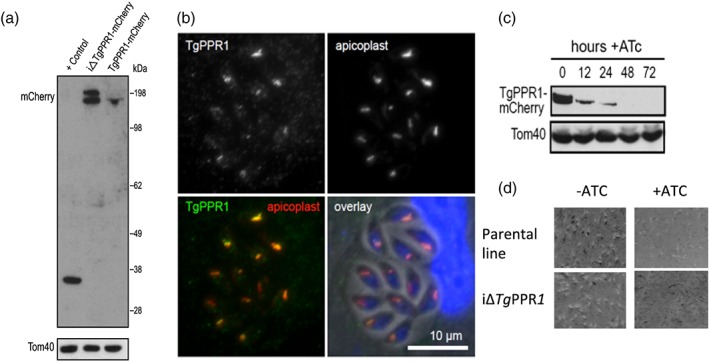
Apicoplast protein *Tg*PPR1 is necessary for parasite growth. (a) Western blot detection of mCherry‐tagged endogenous *Tg*PPR1 using either the t7s4 promoter (iΔ*Tg*PPR1‐mCherry) or the native promoter (*Tg*PPR1‐mCherry). The positive control is a *Toxoplasma gondii* cell line expressing mCherry. Tom40 acts as a loading control. The presence of two bands in the iΔ*Tg*PPR1‐mCherry lane is consistent with a preprocessed PPR targeting intermediate still bearing the predicted apicoplast targeting peptide and mature PPR‐mCherry fusion protein. The position of the 198 kDa standard is shown. (b) Colocation of *Tg*PPR1‐mCherry expressed from the t7s4 promoter with resident apicoplast biotinylated proteins visualised by streptavidin staining. DNA staining in blue, *Tg*PPR1‐mCherry in green, and streptavidin‐stained apicoplast in red. (c) ATc‐induced knock‐down of *Tg*PPR1 assayed over 72 hr. Tom40 acts as a loading control. (d) Eight‐day plaque assay shows normal plaque formation in iΔ*Tg*PPR1 cells without ATc‐induced *Tg*PPR1 depletion, but no plaques with ATc treatment, indicating that PPR is essential for normal growth. A control of the parental cell line is also shown

To test for the localization of the *Toxoplasma Tg*PPR1, a 3′‐PPR1‐mCherry fusion construct was created to tag the endogenous *Tg*PPR gene. A Western blot probed with antibodies to the mCherry reporter protein showed a faint band, corresponding to low expression levels of the mature fusion protein (Figure 2a), but no mCherry signal was apparent by fluorescence microscopy when the cells were examined directly or using an immunofluorescence assay (IFA). When the endogenous PPR1 promoter was replaced by the inducible t7s4 promoter, in cells designated iΔ*Tg*PPR1‐mCherry, a higher expression level of this PPR1‐fusion was seen by Western blot. Furthermore, two bands were present, of apparent sizes consistent with the mature protein and a preprocessed PPR targeting intermediate still bearing the predicted apicoplast targeting peptide (Figure 2a). The presence of these two bands is characteristic for many apicoplast‐targeted proteins (Waller et al., 2000). Given the presence of only the shorter, processed band when PPR1‐mCherry fusion was expressed from the native promoter, this would indicate near‐complete processing under normal expression levels.

To confirm apicoplast localization, we performed an immunofluorescence assay on the iΔPPR1‐mCherry cells (green) co‐stained with streptavidin‐594 (red) that serves as an apicoplast marker due to endogenous biotinylated apicoplast proteins (Chen et al., 2015). Using this cell line, we could detect *Tg*PPR1‐mCherry location and observed it colocating with the apicoplast streptavidin marker (Figure 2b). We conclude that the PPR protein is localized to the apicoplast and is normally expressed at a very low level.

### 2.2 PPR1 is essential for normal growth

As PPR proteins in plants are known to be essential for chloroplast function, we tested if the apicoplast PPR1 was also important for parasite growth. Knock‐down of *Tg*PPR1 in the iΔ*Tg*PPR1‐mCherry line is induced by the addition of anhydrotetracycline (ATc), which represses the t7s4 promoter required for PPR1 expression. ATc treatment of iΔ*Tg*PPR1‐mCherry showed rapid depletion of *Tg*PPR1‐mCherry with the preprocessed protein undetectable within 12 hr of treatment, and no protein detected after 48 hr by Western blot (Figure 2c). To test for a growth phenotype with PPR1 depletion, we used an iΔ*Tg*PPR1 cell line (i.e., t7s4 promoter and no mCherry fusion). Without ATc‐induced depletion, these cells showed normal growth by an 8‐day plaque assay. However, no plaques were observed if cells were treated with ATc. This indicates a strong growth inhibition phenotype in cells depleted of *Tg*PPR1 (Figure 2d). This same growth inhibition phenotype was also seen for iΔ*Tg*PPR‐mCherry with ATc treatment (not shown).

These results are consistent with *Tg*PPR1 gene disruption being reported to have a negative growth phenotype in a genome‐wide CRISPR knockout screen (Sidik et al., 2016). This is also the case for genetic screens of both *P. falciparum* and *Plasmodium berghei*, which show that PPR1 (http://plasmodb.org/plasmo/app/record/gene/PBANKA_1035800 in *P. berghei*) is essential to blood‐stage growth (Bushell et al., 2017; Zhang et al., 2018). Together, these data suggest that the apicoplast PPR1 is broadly essential to apicomplexan parasites.

### 2.3 *Pf*PPR1 binds in vitro transcribed apicoplast transcripts

We then tested if *Pf*PPR1 would bind apicoplast RNA transcripts. Recombinant *Pf*PPR1 was assessed for folding by circular dichroism and analytical ultracentrifugation (AUC), which revealed a folded, alpha helical protein consistent with the alpha helical nature of PPR proteins (Figures S5 and S6). The protein eluted as a dimer from a gel filtration column (see below), and this dimerisation was confirmed by AUC following cleavage of the TRX‐His_6_ tag by HRV 3C protease (Figure S6). The observed folding and dimerisation is consistent with other reported plant PPR proteins, though dimerisation may not occur in vivo (Barkan et al., 2012; Gully et al., 2015; Ke et al., 2013). A SWISS_MODEL homology modelling was carried out (Waterhouse et al., 2018) to compare both *Pf*PPR1 and *Tg*PPR1 with *Arabidopsis thaliana* PPR10, which has a solved structure. Both proteins gave rise to very low GMQU and QMEAN scores, suggesting PPR1 is too divergent to model accurately (Figure S7).

**Figure 3:**
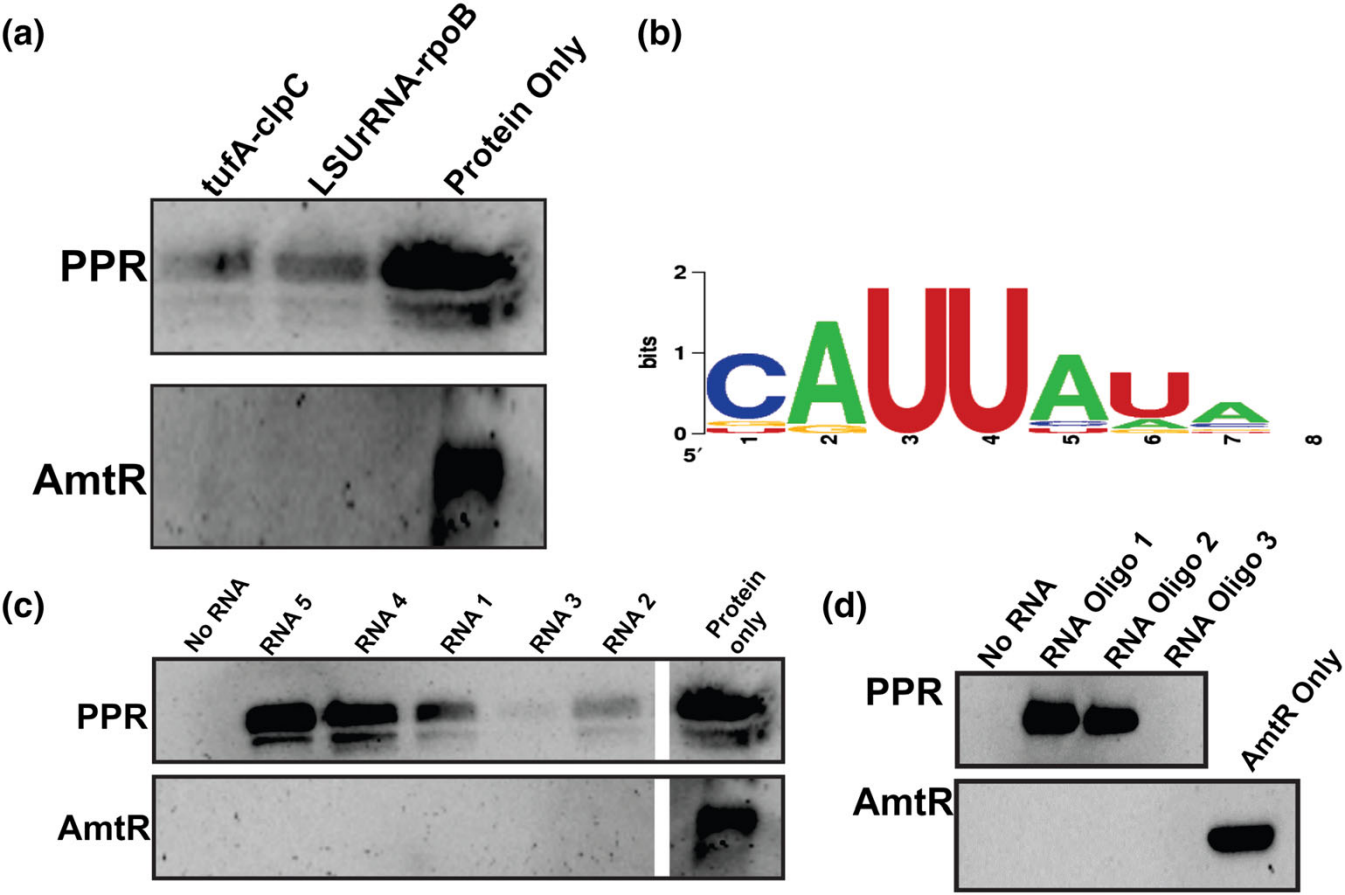
*Pf*PPR1 binds RNA. (a) Pull‐down assay showing that in vitro transcribed apicoplast RNA transcripts (*tufA‐clpC* and *LSUrRNA‐rpoB*) binds to *Pf*PPR1, as shown by Western blot analysis*.* (b) Weblogo of sequences enriched by SELEX, the height of the letter corresponds to the frequency of that nucleotide at that position. (c) RNA transcribed from five clones identified from the final SELEX round were used in a pull‐down experiment. Each 150‐nt RNA molecule contained a constant 125‐nt region (not shown) and a variable 25‐nt region (shown below). Consensus sequences and sequences with slight variation from the consensus are underlined. As a control, RNA pull‐downs were also performed with AmtR. Loading controls are shown to the right of the gel. (d) *Pf*PPR1 pull‐downs with biotinylated RNA oligonucleotides (sequence shown below) each containing the consensus sequence followed by either a U (RNA oligo 1, underlined) or an A (RNA oligo 2, underlined) and a randomly generated six nucleotide sequence (underlined in RNA oligo 3) using an anti‐His antibody demonstrated specificity of PPR for the consensus sequence. The control protein AmtR did not interact with any of the RNA oligonucleotides tested. A repeat of this experiment showed the same results (Figure S8)** RNA1** UUAAACUUGAUGCCCGGCGUUUCAG** RNA2** UUACCGCGCGUAACACCGGGCCUGU** RNA3** UUCGGCGACGGAAAGAGUGAAUCCG **RNA4** UUGUAUUUAUUUAAAAAAUUAUGU** RNA5** UUAUAACUCGCCUAGACGGGAUUAU **RNA oligo 1** ACGACAUUAUAUGGUCGGA** RNA oligo 2** ACGACAUUAUAAGGUCGGA** RNA oligo 3** ACGACAUGACGAGGUCGGA

Based on the results of Nisbet et al. (2016), we transcribed apicoplast RNA *in vitro*. One transcript spanned the *tufA* to *clpC* region of the apicoplast genome as this shows two clearly defined processing sites (at the tRNA‐Phe and tRNA‐Trp genes), and a second transcript spanned the *LSUrRNA* to *rpoB* region, which includes a known processing site at tRNA‐Thr. To test for *Pf*PPR1 binding, we biotinylated the 3′ end of each transcript and performed pull‐down experiments against the *Pf*PPR1 protein. *Pf*PPR1 was observed to bind to both transcripts (Figure 3a). As a control, PPR protein was replaced by a DNA‐binding protein from *Mycobacterium smegmatis* (AmtR), which had been expressed and isolated using the same procedure as *Pf*PPR1 (Petridis et al., 2016). No binding to AmtR was seen. As these experiments were carried out with *Pf*PPR‐TRX fusion protein, we repeated the experiment with the TRX tag removed, with the same results (Figure S8A).

We next carried out gel‐shift assays, and these showed that there was a shift in the migration of apicoplast RNAs when bound to *Pf*PPR1 in a 1:1 molar concentration. There was no shift when *Pf*PPR1 was incubated with RNA transcribed in vitro from either a *Plasmodium* nuclear gene or a *Plasmodium* nuclear gene codon optimised for *E. coli* (Figure S9).

### 2.4 *Pf*PPR1 shows RNA sequence‐specific binding

We next sought to determine if *Pf*PPR1 had a sequence‐specific preference for RNA binding. Analysis of the *Pf*PPR1 amino acid sequence using programs designed for the prediction of plant PPR RNA‐binding sequences (Barkan et al., 2012; Takenaka, Zehrmann, Brennicke, & Graichen, 2013) did not result in any sequence predictions, presumably due to low sequence identity between plant and apicoplast PPR proteins. We therefore performed systematic evolution of ligands by exponential enrichment (SELEX) to determine the sequence specificity of *Pf*PPR1 (Manley, 2013). We constructed a SELEX library with a random 25 nucleotide sequence (N25) in the middle of a 150 nucleotide RNA sequence (Manley, 2013). After four rounds of selection using recombinant His_6_‐TRX‐*Pf*PPR1, sequences containing the motif UUAU were identified in 20 of the 50 final round clones (Figure 3b), with little sequence similarity amongst the other 30 final round clones. This suggests a UUAU binding motif is preferred by *Pf*PPR1. This sequence motif is the same as the UUAU apicoplast RNA processing site previously identified (Nisbet et al., 2016).

To confirm that *Pf*PPR1 binds the RNA molecules containing the identified sequence motif, we performed PPR pull‐down assays using a range of biotinylated 150 nucleotide RNA molecules as “bait” for protein binding. The RNAs were obtained by transcription *in vitro* of five clones isolated in the final round of the SELEX experiment above. RNAs 1, 4, and 5 contained either one or two predicted PPR binding sites, RNA 2 contained a variation of the binding site, and RNA 3 lacked the binding site, as shown in Figure 3c. Full details of sequence are given in the figure legend. Biotin‐labelled transcripts (RNA 1‐5) were bound to streptavidin magnetic resin and incubated with recombinant His_6_‐TRX‐*Pf*PPR1 in a 1:1 protein: RNA molar ratio. Western blots were used to detect PPR protein bound to the “bait” RNA. RNAs 4 and 5 showed strongest bound *Pf*PPR1. These both contain two copies of the consensus binding motifs (Figure 3c). RNAs 1 and 2 showed less bound *Pf*PPR1. These transcripts contain variations of the consensus sites, UUAA and UUAC. Very little protein was detected in the pull‐down with RNA 3, which does not contain the consensus site. This suggests very weak binding to *Pf*PPR1 (Figure 3c). Thus, *Pf*PPR1 binding correlated with presence and copy number of the consensus binding motif.

As a control, the PPR protein was replaced by the *M. smegmatis* DNA‐binding protein (AmtR) that had been expressed and purified in the same manner as *Pf*PPR (Petridis et al., 2016). This showed no binding to the RNA transcripts, demonstrating that pull‐down of the *Pf*PPR1 is specific and is not an artefact of the experiment (Figure 3c). We also repeated the experiment with the *Pf*PPR1 protein minus the His_6_‐TRX tag and with an MBP tag instead of TRX to ensure that the tag on the PPR protein did not interfere with RNA binding, and no difference in the results was seen (Figure S8).

To test further the specificity of *Pf*PPR1 for the UUAU consensus motif, and not for sequences elsewhere on the RNA molecules, we synthesized three 19 nucleotide RNA oligonucleotides with identical flanking sequences but differing at the potential binding motif. RNA oligos 1 and 2 contained the consensus binding sequence, followed by either an AA or an AU, whereas oligo 3 did not contain the binding sequence (RNA oligo 1 *UUAUAA*, RNA oligo 2 *UUAUAU*, RNA oligo 3, *UGACGA*). Each RNA oligonucleotide was biotinylated at the 3′ end and used to pull‐down *Pf*PPR1 or *M. smegmatis* AmtR (control), as above. Western blot analysis showed that *Pf*PPR1 was recovered from RNA oligos 1 and 2 binding assays but not using RNA oligo 3. None of the RNA oligos bound to AmtR (Figure 3d). The results were consistent when repeated twice more (Figure S8B,E). Together, these results demonstrate *Pf*PPR1 shows a strong preference for binding RNA at UUAU.

### 2.5 Confirmation of *Pf*PPR1 RNA binding by gel filtration chromatography

**Figure 4:**
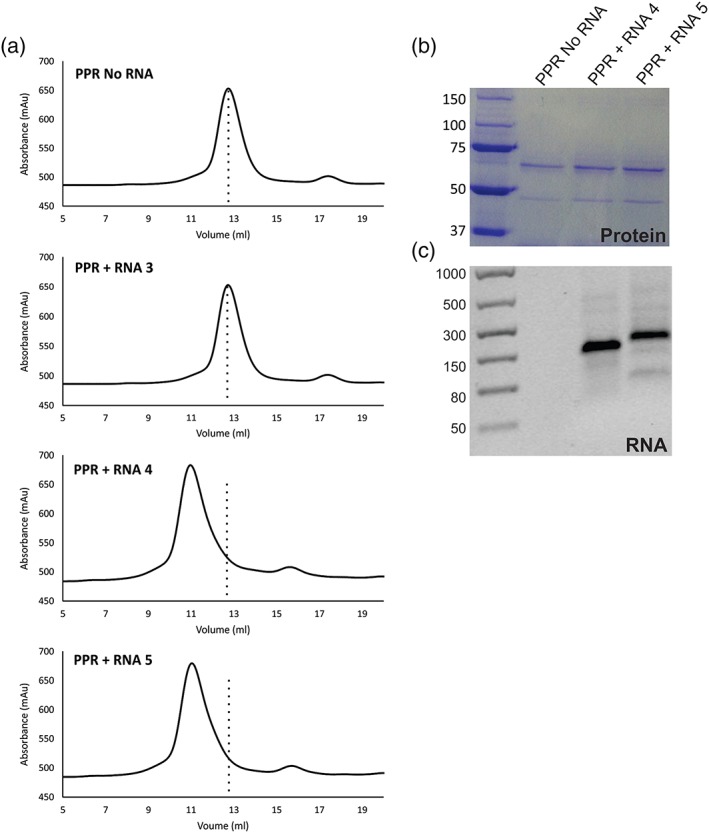
Gel filtration shows a change in elution profile when *Pf*PPR1 is bound to RNA. (a) The elution profiles following gel filtration chromatography of *Pf*PPR1 incubated with RNAs 3, 4, and 5, together with a no RNA control. In each case, a dotted line is shown to indicate where the no RNA control peak is found. (b) Elution peaks for *Pf*PPR1 + no RNA, *Pf*PPR1 + RNA 4, and *Pf*PPR1 + RNA 5 were analysed using SDS‐PAGE. The expected molecular weight of His_6_‐TRX‐*Pf*PPR1 is 72 kDa (ladder sizes in kDa). (c) Agarose gel electrophoresis of RNA extractions from elution peaks *Pf*PPR1 + no RNA, *Pf*PPR1 + RNA 4, and *Pf*PPR1 + RNA5 (ladder in nt). Note that the same results were obtained when the experiment was repeated with *Pf*PPR1 only (i.e., following cleavage of TRX‐His_6_), showing that the result is due to binding to PPR1 and not to TRX‐His_6_), as shown in Figure S10

Many plant PPR proteins were originally thought to bind RNA as a dimer. However, more recent analyses show that, in vivo, the protein instead binds RNA as a monomer (Gully et al., 2015; Ke et al., 2013). We sought to confirm our pull down experiments and determine the stoichiometry of *Pf*PPR1 RNA binding. Analysis by gel filtration chromatography showed the *Pf*PPR1 protein eluted from an analytical gel filtration column at 12.73 ml with a predicted molecular weight of 144 kDa corresponding to a PPR dimer in the absence of any RNA (Figure 4a) and consistent with AUC analysis (Figure S6). To test if *Pf*PPR1 forms a monomer upon RNA binding, *Pf*PPR1 was incubated with each of three 150‐nt RNA baits used above: RNA 4 and RNA 5 both containing two binding motifs and RNA 3 that lacks the motif. Incubations were performed in a 1:1 molar ratio at room temperature for 15 min. Compared with the no RNA control, RNA 4‐ and RNA 5‐incubated *Pf*PPR1 eluted earlier from the column at 10.97 ml consistent with RNA bound to the *Pf*PPR1 dimer (Figure 4a). (If *Pf*PPR1 bound RNA as a monomer, we would expect the elution volume to be greater than 12.73 ml.) The presence of the *Pf*PPR1 protein in the elution fraction was confirmed by SDS‐PAGE (Figure 4b). The protein fraction was also treated with Proteinase K and RNA extracted by phenol/chloroform treatment. When analysed via agarose gel electrophoresis, RNA was visible (Figure 4c). These data confirm binding of *Pf*PPR1 to the consensus RNA motif and that, at least in vitro, this binding occurs as a PPR dimer.

### 2.6 *Pf*PPR1 protects transcripts from ribonuclease activity

**Figure 5:**
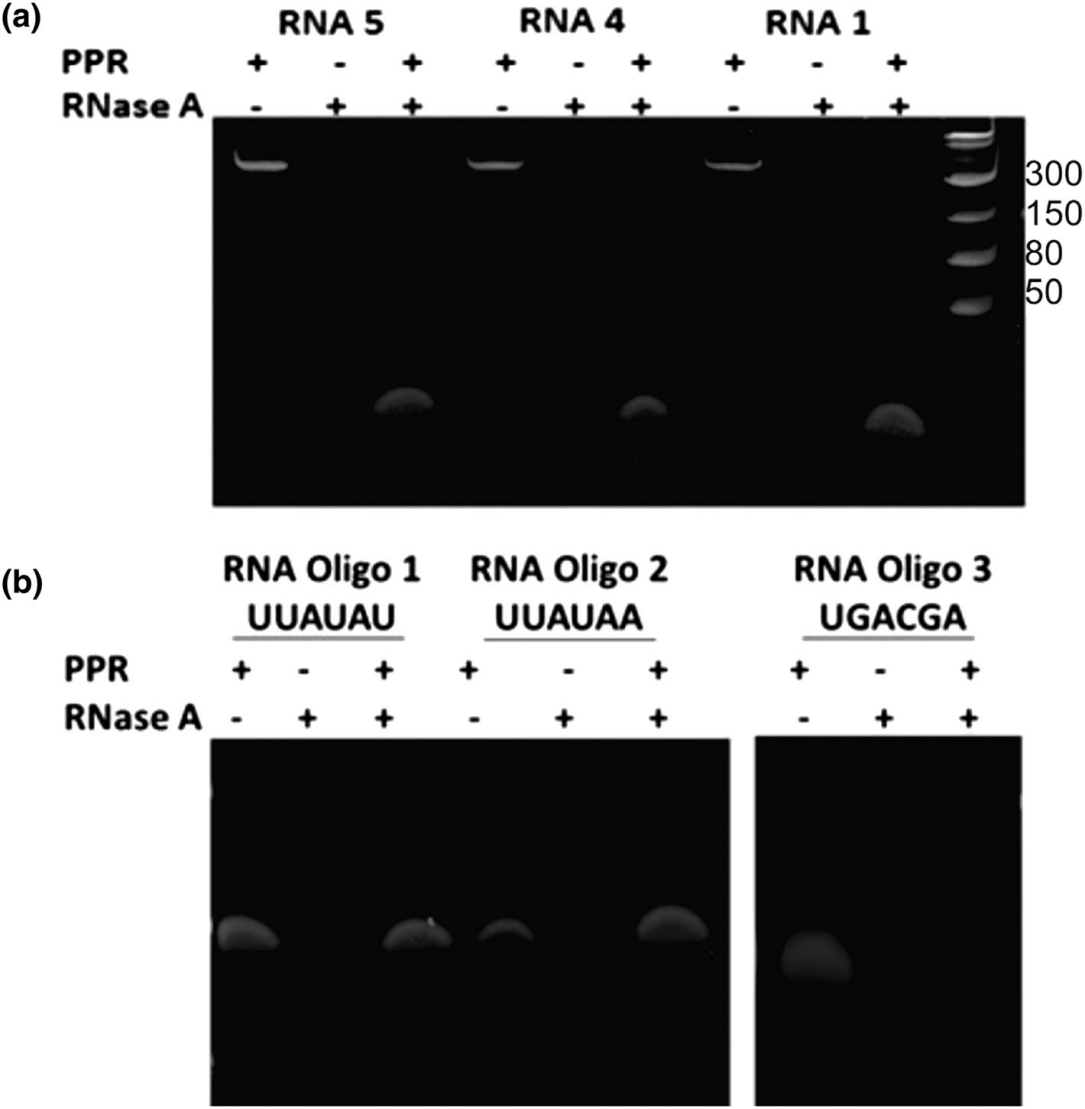
Ribonuclease A protection assays. (a) RNA transcripts 1, 4, and 5 and (b) RNA oligonucleotides 1, 2, and 3 were incubated in a 1:1 molar ratio with *Pf*PPR1 prior to treatment with RNase A. Samples were analysed using a native acrylamide gel (ladder in nt). Positive controls with no *Pf*PPR1 bound to RNA showed complete degradation by RNase A. A repeat of the experiment is show in Figure S11

As the RNA consensus motif is also associated with known transcript cleavage sites, the binding of *Pf*PPR1 is likely to protect RNA from degradation by ribonucleases. We therefore performed RNase protection assays to test for *Pf*PPR1 protected footprints. Three 150‐nt RNA molecules with either one or two consensus binding sites (RNAs 1, 4, and 5, as before) were preincubated with *Pf*PPR1 protein and then incubated with the RNA endonuclease RNase A. In the absence of *Pf*PPR1, the transcripts were completely degraded by RNase A. However, with preincubation with *Pf*PPR1, a small RNA fragment (less than 50 nucleotides) remained after RNase A treatment (Figure 5a). We similarly tested for protection of three 19 nucleotide RNA oligonucleotides (RNA oligos 1, 2, and 3, as before). No degradation was evident when the RNA oligonucleotides 1 and 2 were incubated with *Pf*PPR (Figure 5b). In contrast, RNA oligonucleotide 3, which does not contain the binding sequence, was completely degraded by RNase A in the presence of *Pf*PPR1 (Figure 5b). These data show that *Pf*PPR1 protects RNA from RNAse A activity if it contains the consensus motif.

## 3 DISCUSSION

The discovery of a genome of plastid origin in the malaria parasite *Plasmodium* was a great surprise. Further investigation revealed the presence of a small, but essential organelle subsequently called the apicoplast. We now know a considerable amount about the biochemistry and evolutionary history of this organelle. However, very little is known about how the apicoplast genome itself is transcribed or how posttranscriptional processing is regulated. Here, we present the characterization of the single apicoplast pentatricopeptide repeat (PPR1) protein, increasing our understanding of essential events in apicoplast RNA biology.

PPR1 is a nucleus‐encoded RNA‐binding protein. We show that it is targeted to the apicoplast in both *Plasmodium* and *Toxoplasma*. It is essential for normal growth in *Toxoplasma* and is highly likely also to be essential in *Plasmodium* (Bushell et al., 2017; Zhang et al., 2018). PPR1 is a P‐class PPR protein and has RNA‐binding ability but no catalytic function. *Pf*PPR1 protein is predominantly alpha helical in structure, containing 10 PPR motifs. We show that *Pf*PPR1 binds RNA at a UUAU‐type motif. This motif is found at known cleavage sites for *Plasmodium* apicoplast transcripts, which are often immediately adjacent to tRNA molecules (Nisbet et al., 2016). We show that the protein binds in vitro to apicoplast RNA transcripts containing known cleavage sites and can protect RNA from degradation by RNase A. We have previously shown that the primary transcripts in the *Plasmodium* apicoplast are long and polycistronic (Nisbet et al., 2016). These transcripts are processed into individual mRNA, tRNA, and rRNA molecules often involving cleavage at the same UUAU motif. This motif is often found immediately adjacent to tRNA molecules (Nisbet et al., 2016; Nisbet & McKenzie, 2016).

Given that *Pf*PPR1 lacks any catalytic domain, apicoplast PPR1 binding might protect and define mature transcript ends from RNA exonucleases, facilitating their maturation. Alternatively, it could be that *Pf*PPR1 is involved in recruiting further proteins with endonuclease activity to cleave RNA in order to produce functional transcripts.

The apicomplexan PPR1 is the first plastid PPR protein to be characterized outside the green chloroplast lineage. Unlike other plant and algal taxa, there is only a single plastid PPR protein in apicomplexan plastids. Apicoplast PPR1, like PPR proteins in plants and green algae, binds at specific RNA sites, known to be the sites of RNA processing. Characterization of a single and essential apicoplast PPR protein in *Plasmodium* represents an important advance in our knowledge of apicoplast transcript processing.

## 4 EXPERIMENTAL PROCEDURES

### 4.1 PPR alignment

PPR sequences from *Plasmodium* species were obtained by pBLAST analysis. The PF14_0061 protein alignment was generated in Geneious using a ClustalW algorithm with a BLOSUM cost matrix, a gap open cost of 10 and a gap extend cost of 0.1.

### 4.2 *P. falciparum* culture

Blood stage *P. falciparum* D10 ACP‐GFP (MRA‐568) was cultured according to (Tarr, Nisbet, & Howe, 2011). All work was carried out in accordance with the UK Human Tissue Act 2004.

### 4.3 Expression of recombinant *Pf*PPR1

PF14_0061 (*Pf*PPR1) was codon optimised for *E. coli* and synthesized by GeneArt and cloned into the pOPIN vector system using InFusion (Takara Biotech; Berrow et al., 2007). We cloned *Pf*PPR1 minus the bipartite leader for expression with either an N‐terminal TRX‐His_6_ tag (cloned into pOPINTRX) or an MBP‐His_6_ tag (cloned into pOPINM). As there is very little homology amongst leader sequences between *Plasmodium* species (Parsons, Karnataki, Feagin, & DeRocher, 2007), we took the start of mature *Pf*PPR1 to correspond to the region of homology with other *Plasmodium* PPR1 proteins. We further tested the start of the mature protein by analysing *Pf*PPR1 with and without the predicted 120 amino acid bipartite leader using TPRPred to ensure we were not removing or truncating any PPR motifs. Expression was carried out in BL21(DE3) pLysS in ZY‐5052 autoinduction medium (100 μg ml^−1^ of ampicillin and 34 μg ml^−1^ of chloramphenicol). PPR1 was purified via HisTrap column on an AKTA FPLC (GE Healthcare). The protein was eluted from the column using an imid‐azole gradient. Fractions containing His_6_‐TRX‐*Pf*PPR1 were pooled and concentrated using a Vivaspin concentrator (10,000 molecular weight cut‐off) before gel filtration chromatography using a S200 10/300 analytical size exclusion column. Fractions containing protein were analysed by SDS‐PAGE and confirmed by MALDI‐TOF MS analysis. The His_6_‐TRX tag was cleaved from *Pf*PPR1 by incubation of the His_6_‐TRX‐*Pf*PPR1 fusion protein with 1% (v/v) recombinant HRV 3C protease followed by gel filtration chromatography. Full details are given in the Supporting Information.

### 4.4 *Pf*PPR1 antibody production and purification

Recombinant *Pf*PPR1 minus the His_6_‐TRX tag was used to generate *Pf*PPR1 antibodies in two rabbits by Pacific Immunology. Preimmune serum was taken followed by injection of recombinant *Pf*PPR1 plus adjuvant. Four production bleeds were taken at 2‐week intervals followed by a final bleed after 3 months.

To purify the anti‐*Pf*PPR1 antibody, 150 μl of His affinity resin was washed with 1 ml water and three times with 1 ml of 1 × TBS pH 7.6. Two hundred and fifty microlitres of His_6_‐TRX‐*Pf*PPR1 (1.7 mg ml^−1^) were added and incubated at room temperature for 15 min with agitation. The supernatant was removed, and the resin washed three times in 1 × TBS. One millilitre of antibody serum was added and incubated for 1 hr at room temperature with gentle mixing. The supernatant was removed, and the resin washed four times in 1 × TBS. The bound anti‐*Pf*PPR1 antibody was eluted by addition of 200 μl 0.1 M glycine pH 2.5. The supernatant was neutralised by the addition of 20 μl 1 M Tris.HCl pH 8.5 to produce purified antibody.

### 4.5 *Pf*PPR1 Western blot against *P. falciparum* 3D7 lysate

Asynchronous *P. falciparum* 3D7 parasites were grown in an 8 ml culture of washed human red blood cells until a total parasitaemia of 10% was reached. The culture was spun at 600 × *g* for 20 min to pellet cells. The supernatant was removed, and 1 ml 0.05% saponin in 1 × phosphate‐buffered saline (PBS) was added to lyse red blood cells. The lysed cells were then spun at 1,000 × *g* for 20 min to pellet parasites. The supernatant was removed and parasites resuspended in 1 ml 1 × PBS and transferred to a 1.5‐ml tube. The parasites were then pelleted again by spinning at 3,000 × *g* for 10 min, supernatant removed, and washed again with 1 ml 1 × PBS. The parasites were spun again at 3,000 × *g* for 10 min, supernatant removed, and the parasite pellet resuspended in 30 μl 50 mM Tris pH 8.0, 200 mM NaCl, and 30 μl 4 × SDS loading dye. Samples were then heated to 100°C in a water bath for 15 min to lyse parasites.

Twenty microlitres of lysed parasites from above and 50 ng of recombinant *Pf*PPR1 protein (positive control) were loaded onto 10% SDS‐PAGE gel alongside 5 μl of Hyperladder I. SDS‐PAGE gels were run at 100 V until sample dye reached the bottom. Proteins from SDS‐PAGE gels were transferred to polyvinylidene difluoride membrane via wet blot in transfer buffer (25 mM Tris, 192 mM glycine, 20% methanol, and 0.01% SDS) at 100 V for 1 hr at room temperature. The membrane was then removed from the transfer apparatus and stained with Ponceau to check for complete transfer. The membrane was then blocked in 10% *w/v* low‐fat milk powder in 1 × TBS‐T (Tris‐buffered saline +0.01% Tween 20) at 4°C overnight. Purified *Pf*PPR1 polyclonal antibody was prepared at a 1:1000 dilution in blocking solution, and the membrane placed face down in this solution in a humidity chamber for 1 hr at room temperature. The membrane then underwent five 5‐min washes in 1 × TBS‐T. A 1:2000 goat anti‐rabbit HRP conjugate antibody solution was prepared in 1 ml of blocking solution. The membrane was placed face down in this solution in a humidity chamber for 1 hr at room temperature. The membrane then underwent five 5‐min washes in 1 × TBS‐T. The Western blot was developed using the Thermo Supersignal Femtomole Chemiluminescent substrate in a 1:1 ratio. Blots were immediately visualised using a Genebox with varying exposure times.

### 4.6 Immunofluorescence microscopy for localization of *Pf*PPR1

Asynchronous *P. falciparum* D10 ACP_L_‐GFP cultures were used for immunofluorescence microscopy experiments, essentially following (Tonkin et al., 2004). Purified anti‐*Pf*PPR1 antibody was diluted 1:1000 with blocking solution, and AlexaFluor‐568 Donkey anti‐rabbit IgG was diluted 1:2000 with blocking solution. Slides were visualised using an Olympus IX81 confocal microscope at 60× magnification. Two channels, one to detect GFP fluorescence (eGFP) and the other to detect AlexaFluor‐568 fluorescence (Cy3) plus bright field were used to image slides. Images were overlaid using Fluoview version 5.0 microscopy software.

### 4.7 *T. gondii* cell culture and generation of cell lines

*T. gondii* RH Δku80/TATi tachyzoites were grown by inoculation in confluent human foreskin fibroblast (HFF) cells as previously described (Striepen & Soldati, 2007). Endogenous promoter replacement with the t7s4 promoter was induced by Cas9‐mediated cleavage at the 5′ end of the *ppr* locus. Plasmid pCRISPR/Cas9‐GFP_PPR‐sgRNA (see the Supporting Information) was assembled using the Golden Gate assembly method (Engler et al., 2014). A linear donor molecule including the t7s4 promoter and DHFR resistance gene was amplified from plasmid pPR2‐HA3 (Katris et al., 2014) with primers KDPPR‐Fwd and KDPPR‐Rev that included flanking sequences directed to the 5′ end of the *ppr* locus on either side of the Cas9 cleavage site. pCRISPR/Cas9‐GFP_PPR‐sgRNA and this linear donor were cotransfected into *T. gondii* TATiDku80 parasites (a kind gift from Lilach Sheiner and Boris Striepen, U. Georgia; Sheiner et al., 2011) and transformants selected on pyrimethamine and cloned by limiting dilution (Katris et al., 2014; Striepen & Soldati, 2007). Successful promoter replacement was verified by PCR from genomic DNA. Endogenous in‐frame 5′ tagging of the *ppr* locus with reporter protein gene *mCherry* was achieved using plasmid pPPR‐mCherry_CAT (see the Supporting Information) assembled using the Golden Gate method. Prior to transfection of parasites this plasmid was linearised with *Bam*HI, and transformants were selected with chloramphenicol and cloned by limiting dilutions (Striepen & Soldati, 2007).

### 4.8 Toxoplasma PPR assays

Western blot detection of SDS‐PAGE resolved whole cell lysates was performed using a rabbit anti‐mCherry (1/1000 dilution; Abcam) and anti‐TOM40 as a control (Katris et al., 2014). Immunofluorescence microscopy was performed on intracellular tachyzoites using anti‐mCherry (1/1000) with secondary antibody AlexaFluor‐488 Goat anti‐rabbit IgG (Life Technology). Apicoplasts were co‐stained with AlexaFluor‐594 anti‐Steptavidine (Life Technology; Chen et al., 2015). Samples were mounted with ProLong Diamond antifade mountant with DAPI (Invitrogen) and sealed with nail polish. Cells were imaged using an Inverted Nikon Eclipse Ti microscope, a Nikon objective lens (Plan APO, 100×/1.45 oil), and a Hamamatsu C11440, ORCA Flash 4.0 camera.

PPR knock‐down was induced by addition of ATc (0.5 μg ml^−1^) to the growth medium upon parasite inoculation of HFF cells. For plaque assays, extracellular parasites were filtered, counted by haemocytometer, and 500 parasites added to 25‐cm^2^ tissue culture flasks containing a confluent monolayer of HFF cells. Cultures were incubated for 8 days. To visualise plaque sizes in the presence or absence of ATc, flasks were aspirated, fixed with 5 ml 100% ethanol (5 min), stained with 5 ml of crystal violet solution (15 min) then washed once with PBS, and dried before imaging (Jacot, Meissner, Sheiner, Soldati‐Favre, & Striepen, 2014).

### 4.9 SELEX for determination of *Pf*PPR1 RNA sequence specificity

A SELEX library was constructed as in (Manley, 2013). SELEX using recombinant His_6_‐TRX‐*Pf*PPR1 was carried out as per the protocol in (Manley, 2013). Four rounds of selection were performed, following which PCR products were cloned into pGEM‐T easy (Promega) and transformed into chemically competent *E. coli* DH5α and plated onto LB agar (100 μg ml^−1^ of ampicillin, 0.1 mM IPTG, and 40 μg ml^−1^ of X‐gal). Plasmids were extracted from 50 clones and sequenced. In addition, 10 input clones were sequenced from each round to determine enrichment.

### 4.10 In vitro RNA transcription and 3′ end biotinylation

Apicoplast *P. falciparum* apicoplast PCR products were obtained using the following primers: LSUrRNA Fwd/rpoB Rev and tufA Fwd. clpC Rev. T7 promoter sequences (TAATACGACTCACTATAG) were added in a further round of PCR with the T7 promoter sequence appended to the 5′ end of the forward primer. Additionally, PCR products were obtained from SELEX clones (above) using Phusion Polymerase (NEB) according to the manufacturer's instructions and primers pGEM FWD and pGEM REV (for primer sequences see Table S3) designed to encompass the T7 promoter. The Ambion T7 MEGAScript Kit was used for *in* *vitro* transcription according to manufacturer's instructions. The Ambion T7 MEGAScript Kit was used for *in vitro* transcription. For 3′ end biotinylation, 50 pmol of RNA transcript was heated at 85°C for 3–5 min. Once on ice, 3 μl 10 × T4 RNA ligase buffer (NEB), 1 μl of rRNasin (Promega), 50 pmol of RNA, 1 μl of pCp Biotin, 2 μl of T4 RNA Ligase (NEB), water to 15 μl, and 15 μl 30% PEG was added. Ligation reactions were incubated overnight at 16°C. Seventy microlitres of water was added followed by 100 μl of chloroform:isoamyl alcohol (49:1). Reactions were centrifuged at 13,000 × *g* for 3 min and the upper phase (aqueous layer) removed and transferred to a new tube; 10 μl 3 M sodium acetate pH 5.2 and 250 μl 100% ethanol were added to precipitate RNA and stored at −20°C. The RNA was pelleted by centrifugation at 13,000 × *g*, 4°C for 20 min, washed with 70% ethanol, and centrifuged again. The pellet was resuspended in 20 μl of water, and RNA was quantified with a Nanodrop‐1000.

### 4.11 Biotinylated RNA‐*Pf*PPR1 pull‐downs

Twenty‐five microlitres of resuspended streptavidin magnetic beads (Thermo Scientific) were washed in 1 ml of water and then three times in binding buffer (10 mM HEPES pH 7.5, 20 mM KCl, 1 mM MgCl_2_, and 1 mM DTT) and resuspended in 20 μl of binding buffer. Four hundred nanomolar biotinylated RNA transcript was added and incubated at 4°C for 15 min. The beads were washed three times in 1 ml of binding buffer and resuspended in 20 μl of binding buffer; 800 nM His_6_‐TRX‐*Pf*PPR1 was added followed by incubation at room temperature for 15 min with gentle agitation. The beads were washed three times with 1 ml of binding buffer and resuspended in 25 μl 4 × SDS loading dye, heated to 100°C for 5 min and loaded onto a 4–15% SDS‐PAGE gel (BioRad). Following PAGE, the samples were transferred via wet blot to polyvinylidene difluoride membrane for Western blot analysis with a mouse‐anti‐His antibody and a secondary goat anti‐mouse antibody conjugated with HRP. Western blots were visualised using Western Bright Quantum (Advasnsta) chemiluminiscent substrate and exposed using a CCD camera (Genebox).

### 4.12 Gel filtration chromatography

His_6_‐TRX‐*Pf*PPR1 was incubated with RNA transcripts in a 1:1 molar ratio in 1 × binding buffer (10 mM HEPES pH 7.5, 20 mM KCl, 1 mM MgCl_2_, and 1 mM DTT) for 15 min at room temperature. A control without RNA was included in these reactions. Samples were analysed using a S200 10/300 analytical gel filtration column preequilibrated in 50 mM Tris pH 8.0, 200 mM NaCl, 5 mM DTT.

### 4.13 RNase A protection assays

Ten micromolar RNA, 1 × binding buffer (10 mM HEPES pH 7.5, 20 mM KCl, 1 mM MgCl_2_, and 1 mM DTT) and 10 μM *Pf*PPR1 in a volume of 20 μl were incubated for 15 min at room temperature, followed by addition of 0.01% (v/v) 20 mg ml^−1^ of RNase A and incubated at 37°C with shaking for 30 min. Reactions were stopped by the addition of 10 μl 2 × formamide loading dye (95% formamide, 0.025% w/v bromophenol blue, 0.025% xylene cyanol FF, 5 mM EDTA) and heating to 70°C for 5 min. Assay reactions were analysed by 12% urea‐denaturing PAGE gel and visualised with SYBR Safe nucleic acid stain. Three controls (no *Pf*PPR1, no RNA, and no RNase) were carried out, where the reagent was replaced by water.

## AUTHOR CONTRIBUTIONS

J.L.H., I.L., E.C., M.E., A.V., and R.E.R.N. performed the experiments. R.F.W. provided expertise in *Toxoplasma*. J.L.H., R.F.W., C.J.H., and R.E.R.N. drafted the manuscript. All authors read and approved the final manuscript.

## Supporting information


**Figure S1.** PPR proteins in *Plasmodium* and *Toxoplasma.* A. PPR predictions for *Plasmodium falciparum* and *Toxoplasma gondii* PPR1. B. PPR1 and PPR2 proteins across the *Plasmodium* species*.* Mature (i.e. without targeting sequence) PPR1 (apicoplast) and PPR2 (mitochondrial) were aligned across selected *Plasmodium* species using ClustalW, and a phylogenetic tree inferred using PhyML.

Table S1. Predicted apicoplast localization.
Table S2. Conditions trialled for detection of PfPPR1 *in P. falciparum 3D7* lysate by Western Blot
Table S3. Primers used in this study
Figure S2. Alignment of PPR1 proteins from *Plasmodium*, *Toxoplasma* and PPR10 from *Arabidopsis thaliana*. *Plasmodium* species as in Supplemental Figure 1.

**Figure S3.** **Gel filtration chromatography of His**_**6**_ **‐TRX‐PfPPR1 and corresponding SDS‐PAGE gel**. Blue line represents absorbance at 280 nm, red line absorbance at 260 nm and brown line conductivity. The trace for absorbance at 280 nm (blue line) shows four major peaks eluted from the S200 10/300 column. Based on calibration of the S200 10/300 column, the second peak corresponds to a molecular weight of approximately 135 kDa, and the estimated molecular weight of the His_6_‐TRX‐*Pf*PPR1 dimer is 141.6 kDa. Spontaneous cleavage of the TRX‐Histag is observed in the second peak, corresponding to PPR protein without the His_6_‐TRX tag (estimated molecular weight of 56.8 kDa) with a smaller amount in the third peak. The fourth peak corresponds to the His_6_‐TRX tag only (14 kDa).

**Figure S4.** **Western blot of purified *Pf*PPR1 protein and *P. falciparum* 3D7 lysate using the purified polyclonal anti‐*Pf*PPR1 antibody.** Purified PfPPR1 protein was run on 10% SDS‐PAGE gel along with *P. falciparum 3D7* cell lysate. After incubation with the purified *Pf*PPR1 polyclonal rabbit antibody and a secondary goat anti‐rabbit antibody conjugated to HRP, no *Pf*PPR1 protein could be detected in the *P. falciparum* 3D7 lysate (Panel A).. The positive control (recombinant *Pf*PPR1) shows a band of the correct size (*Pf*PPR1 + TRX His_6_ ~ 72.8 kDa) .The ponceau stained gel (Panel B) shows good transfer of proteins of all molecular weights. Size markers are shown in kDa.

**Figure S5.** **Circular Dichroism (CD) spectrum of purified *Pf*PPR1.** *Pf*PPR1 minus His‐TRX tag at 25°C in 10 mM potassium phosphate pH 8.0, 50 mM Na fluoride. Spectrum is typical of that for an alpha‐helical protein.

**Figure S6.** **Analytical ultracentrifugation (AUC) sedimentation velocity data for *Pf*PPR1 minus His**_**6**_ **‐TRX tag**. The residuals are from the fit with the continuous c(s) distribution model. Component sedimentation coefficient distribution for PPR at 1.8 mg/mL showing populations of dimeric (fitted mass of 109 kDa) and higher‐order species, fitting to a uniform frictional ratio of Fk,w = 1.378. The r.m.s.d. was 0.016.

**Figure S7**. **SWISS‐MODEL** analysis of PPR1 from both *Toxoplasma* and *Plasmodium.*

**Figure S8.** ***Pf*PPR1 – RNA pull down assays.** (A) Five 150 nt RNA molecules (RNAs 1‐5, sequences as shown in Figure 3) and apicoplast RNA transcripts (*LSU rRNA – rpoB* and *tufA – clpC*) were used in a pull‐down experiment. Biotinylated RNA was bound to streptavidin beads and used as ‘bait' to pull down *Pf*PPR1 protein (with the TRX‐His_6_ tag removed). Bound *Pf*PPR1 was protein detected using a purified polyclonal anti‐*Pf*PPR1 antibody from rabbit. Loading controls (PPR1 only) and a no RNA control reaction showed no non‐specific binding in the absence of RNA. (B) The same pull down experiment using RNA oligonucleotides 1 – 3. The same result was obtained when the experiment was repeated (E). Details of RNA sequences are given in the legend to Figure 3, and specific sequences are shown (C and D).

**Figure S9.** ***Pf*PPR1 shows specific binding to apicoplast RNA transcripts.** *Pf*PPR1 binding to apicoplast RNA transcripts was tested in a gel shift experiment. Recombinant *Pf*PPR1 (minus TRX‐His_6_) causes a shift in the migration of *in vitro* transcribed apicoplast RNA molecules following incubation for one hour (Panel A). No shift is seen in the RNA if it is not bound to PPR1. Panel B shows that no shift is seen when PPR1 is incubated with *in vitro* transcribed RNA from a nuclear encoded *P. falciparum* gene (PF11_0264, or from an *E. coli* codon‐optomized *P. falciparum* gene (PF14_0061) and a *P. falciparum 3D7* nuclear gene (PF11_0264).

**Figure S10** **Gel filtration shows a change in elution profile when *Pf*PPR1 is bound to RNA.** This is a repeat of the data shown in Figure 4, showing that the change in mobility following gel filtration is reproducible.

**Figure S11.** **Ribonuclease A protection assays.** RNA transcripts 1, 4 and 5 and RNA oligos 1,2 and 3 were incubated in a 1:1 molar ratio with *Pf*PPR1 prior to treatment with RNase A. Samples were analyzed using a native acrylamide gel (ladder in nt). Experiments with no *Pf*PPR1 bound to RNA showed complete degradation by RNase A

Data S1: Supporting information

## References

[cmi13108-bib-0001] Bannai, H. , Tamada, Y. , Maruyama, O. , Nakai, K. , & Miyano, S. (2002). Extensive feature detection of N‐terminal protein sorting signals. Bioinformatics, 18(2), 298–305. 10.1093/bioinformatics/18.2.298 11847077

[cmi13108-bib-0002] Barkan, A. , Rojas, M. , Fujii, S. , Yap, A. , Chong, Y. S. , Bond, C. S. , & Small, I. (2012). A combinatorial amino acid code for RNA recognition by pentatricopeptide repeat proteins. PLoS Genetics, 8(8), e1002910 10.1371/journal.pgen.1002910 22916040 PMC3420917

[cmi13108-bib-0003] Barkan, A. , & Small, I. (2014). Pentatricopeptide repeat proteins in plants. Annual Review of Plant Biology, 65(1), 415–442. 10.1146/annurev-arplant-050213-040159 24471833

[cmi13108-bib-0004] Bender, A. , van Dooren, G. G. , Ralph, S. A. , McFadden, G. I. , & Schneider, G. (2003). Properties and prediction of mitochondrial transit peptides from *Plasmodium falciparum* . Molecular and Biochemical Parasitology, 132(2), 59–66. 10.1016/j.molbiopara.2003.07.001 14599665

[cmi13108-bib-0005] Berrow, N. S. , Alderton, D. , Sainsbury, S. , Nettleship, J. , Assenberg, R. , Rahman, N. , … Owens, R. J. (2007). A versatile ligation‐independent cloning method suitable for high‐throughput expression screening applications. Nucleic Acids Research, 35(6), e45–e45. 10.1093/nar/gkm047 17317681 PMC1874605

[cmi13108-bib-0007] Bryant, N. , Lloyd, J. , Sweeney, C. , Myouga, F. , & Meinke, D. (2011). Identification of nuclear genes encoding chloroplast‐localized proteins required for embryo development in *Arabidopsis* . Plant Physiology, 155(4), 1678–1689. 10.1104/pp.110.168120 21139083 PMC3091104

[cmi13108-bib-0008] Bushell, E. , Gomes, A. R. , Sanderson, T. , Anar, B. , Girling, G. , Herd, C. , … Billker, O. (2017). Functional profiling of a *Plasmodium* genome reveals an abundance of essential genes. Cell, 170(2), 260–272. e268. 10.1016/j.cell.2017.06.030 28708996 PMC5509546

[cmi13108-bib-0009] Chen, A. L. , Kim, E. W. , Toh, J. Y. , Vashisht, A. A. , Rashoff, A. Q. , Van, C. , … Bradley, P. J. (2015). Novel components of the *Toxoplasma* inner membrane complex revealed by BioID. MBio, 6(1), e02357‐14 10.1128/mBio.02357-14 25691595 PMC4337574

[cmi13108-bib-0010] Engler, C. , Youles, M. , Gruetzner, R. , Ehnert, T.‐M. , Werner, S. , Jones, J. D. G. , … Marillonnet, S. (2014). A golden gate modular cloning toolbox for plants. ACS Synthetic Biology, 3(11), 839–843. 10.1021/sb4001504 24933124

[cmi13108-bib-0011] Foth, B. J. , Ralph, S. A. , Tonkin, C. J. , Struck, N. S. , Fraunholz, M. , Roos, D. S. , … McFadden, G. I. (2003). Dissecting apicoplast targeting in the malaria parasite *Plasmodium falciparum* . Science, 299(5607), 705–708. 10.1126/science.1078599 12560551

[cmi13108-bib-0012] García‐Mauriño, S. M. , Díaz‐Quintana, A. , Rivero‐Rodríguez, F. , Cruz‐Gallardo, I. , Grüttner, C. , Hernández‐Vellisca, M. , & Díaz‐Moreno, I. (2018). A putative RNA binding protein from *Plasmodium vivax* apicoplast. FEBS Open Bio, 8(2), 177–188. 10.1002/2211-5463.12351 PMC5794462 29435408

[cmi13108-bib-0013] Gardner, M. J. , Williamson, D. H. , & Wilson, R. J. M. (1991). A circular DNA in malaria parasites encodes an RNA polymerase like that of prokaryotes and chloroplasts. Molecular and Biochemical Parasitology, 44(1), 115–123. 10.1016/0166-6851(91)90227-W 2011147

[cmi13108-bib-0014] Gully, B. S. , Cowieson, N. , Stanley, W. A. , Shearston, K. , Small, I. D. , Barkan, A. , & Bond, C. S. (2015). The solution structure of the pentatricopeptide repeat protein PPR10 upon binding *atpH* RNA. Nucleic Acids Research, 43(3), 1918–1926. 10.1093/nar/gkv027 25609698 PMC4330388

[cmi13108-bib-0015] Howe, C. J. (1992). Plastid origin of an extrachromosomal DNA molecule from *Plasmodium*, the causative agent of malaria. Journal of Theoretical Biology, 158, 199–205. 10.1016/S0022-5193(05)80718-0 1474844

[cmi13108-bib-0016] Jacot, D. , Meissner, M. , Sheiner, L. , Soldati‐Favre, D. , & Striepen, B. (2014). Chapter 17—Genetic manipulation of *Toxoplasma gondii* In Weiss L. M., & Kim K. (Eds.), Toxoplasma Gondii (Second ed.) (pp. 577–611). Boston: Academic Press 10.1016/B978-0-12-396481-6.00017-9

[cmi13108-bib-0017] Karpenahalli, M. R. , Lupas, A. N. , & Söding, J. (2007). TPRpred: a tool for prediction of TPR‐, PPR‐ and SEL1‐like repeats from protein sequences. BMC Bioinformatics, 8, 2–2. 10.1186/1471-2105-8-2 17199898 PMC1774580

[cmi13108-bib-0018] Katris, N. J. , van Dooren, G. G. , McMillan, P. J. , Hanssen, E. , Tilley, L. , & Waller, R. F. (2014). The apical complex provides a regulated gateway for secretion of invasion factors in *Toxoplasma* . PLoS Pathogens, 10(4), e1004074 10.1371/journal.ppat.1004074 24743791 PMC3990729

[cmi13108-bib-0019] Ke, J. , Chen, R.‐Z. , Ban, T. , Zhou, X. E. , Gu, X. , Tan, M. H. E. , … Xu, H. E. (2013). Structural basis for RNA recognition by a dimeric PPR‐protein complex. Nature Structural & Molecular Biology, 20, 1377–1382. 10.1038/nsmb.2710 24186060

[cmi13108-bib-0021] Lurin, C. , Andrés, C. , Aubourg, S. , Bellaoui, M. , Bitton, F. , Bruyère, C. , … Small, I. (2004). Genome‐wide analysis of *Arabidopsis* pentatricopeptide repeat proteins reveals their essential role in organelle biogenesis. The Plant Cell, 16(8), 2089–2103. 10.1105/tpc.104.022236 15269332 PMC519200

[cmi13108-bib-0022] Manley, J. L. (2013). SELEX to identify protein‐binding sites on RNA. Cold Spring Harbor Protocols, 2013(2), 156–163. 23378656 10.1101/pdb.prot072934 PMC3703746

[cmi13108-bib-0023] Manna, S. (2015). An overview of pentatricopeptide repeat proteins and their applications. Biochimie, 113, 93–99. 10.1016/j.biochi.2015.04.004 25882680

[cmi13108-bib-0024] McFadden, G. I. , Reith, M. E. , Munholland, J. , & Lang‐Unnasch, N. (1996). Plastid in human parasites. Nature, 381, 482 10.1038/381482a0 8632819

[cmi13108-bib-0025] Mehlin, C. , Boni, E. , Buckner, F. S. , Engel, L. , Feist, T. , Gelb, M. H. , … Hol, W. G. J. (2006). Heterologous expression of proteins from *Plasmodium falciparum*: Results from 1000 genes. Molecular and Biochemical Parasitology, 148(2), 144–160. 10.1016/j.molbiopara.2006.03.011 16644028

[cmi13108-bib-0027] Nielsen, H. (2017). Predicting secretory proteins with SignalP In Kihara D. (Ed.), Protein Function Prediction: Methods and Protocols (pp. 59–73). New York, NY: Springer New York 10.1007/978-1-4939-7015-5_6 28451972

[cmi13108-bib-0028] Nisbet, R. E. R. , Kurniawan, D. P. , Bowers, H. D. , & Howe, C. J. (2016). Transcripts in the *Plasmodium* apicoplast undergo cleavage at tRNAs and editing, and include antisense sequences. Protist, 167, 377–388. 10.1016/j.protis.2016.06.003 27458998 PMC4995348

[cmi13108-bib-0029] Nisbet, R. E. R. , & McKenzie, J. L. (2016). Transcription of the apicoplast genome. Molecular and Biochemical Parasitology, 210(1‐2), 5–9. 10.1016/j.molbiopara.2016.07.004 27485555 PMC5404108

[cmi13108-bib-0030] Parsons, M. , Karnataki, A. , Feagin, J. E. , & DeRocher, A. (2007). Protein trafficking to the apicoplast: Deciphering the apicomplexan solution to secondary endosymbiosis. Eukaryotic Cell, 6(7), 1081–1088. 10.1128/ec.00102-07 17513565 PMC1951102

[cmi13108-bib-0031] Petridis, M. , Vickers, C. , Robson, J. , McKenzie, J. L. , Bereza, M. , Sharrock, A. , … Cook, G. M. (2016). Structure and function of AmtR in *Mycobacterium smegmatis*: Implications for post‐transcriptional regulation of urea metabolism through a small antisense RNA. Journal of Molecular Biology, 428(21), 4315–4329. 10.1016/j.jmb.2016.09.009 27640309

[cmi13108-bib-0033] Prikryl, J. , Rojas, M. , Schuster, G. , & Barkan, A. (2011). Mechanism of RNA stabilization and translational activation by a pentatricopeptide repeat protein. Proceedings of the National Academy of Sciences, 108(1), 415–420. 10.1073/pnas.1012076108 PMC3017144 21173259

[cmi13108-bib-0034] Rackham, O. , & Filipovska, A. (2012). The role of mammalian PPR domain proteins in the regulation of mitochondrial gene expression. Biochimica et Biophysica Acta, 1819(9‐10), 1008–1016. 10.1016/j.bbagrm.2011.10.007 22051507

[cmi13108-bib-0035] Sheiner, L. , Demerly, J. L. , Poulsen, N. , Beatty, W. L. , Lucas, O. , Behnke, M. S. , … Striepen, B. (2011). A systematic screen to discover and analyze apicoplast proteins identifies a conserved and essential protein import factor. PLoS Pathogens, 7(12), e1002392 10.1371/journal.ppat.1002392 22144892 PMC3228799

[cmi13108-bib-0036] Sidik, S. M. , Huet, D. , Ganesan, S. M. , Huynh, M.‐H. , Wang, T. , Nasamu, A. S. , … Lourido, S. (2016). A genome‐wide CRISPR screen in *Toxoplasma* identifies essential apicomplexan genes. Cell, 166(6), 1423–1435.e1412. 10.1016/j.cell.2016.08.019 27594426 PMC5017925

[cmi13108-bib-0037] Sosso, D. , Canut, M. , Gendrot, G. , Dedieu, A. , Chambrier, P. , Barkan, A. , … M. Rogowsky, P. (2012). PPR8522 encodes a chloroplast‐targeted pentatricopeptide repeat protein necessary for maize embryogenesis and vegetative development. Journal of Experimental Botany, 63(16), 5843–5857. 10.1093/jxb/ers232 22945943 PMC3467297

[cmi13108-bib-0038] Sosso, D. , Mbelo, S. , Vernoud, V. , Gendrot, G. , Dedieu, A. , Chambrier, P. , … Rogowsky, P. M. (2012). PPR2263, a DYW‐subgroup pentatricopeptide repeat protein, is required for mitochondrial transcript editing, mitochondrion biogenesis, and maize growth. The Plant Cell, 24(2), 676–691. 10.1105/tpc.111.091074 22319053 PMC3315240

[cmi13108-bib-0039] Striepen, B. , & Soldati, D. (2007). Genetic manipulation of *Toxoplasma gondi*i In Weiss L. M., & Kim K. (Eds.), Toxoplasma gondii. The Model Apicomplexan‐Perspectives and Methods (pp. 391–415). London: Elsevier 10.1016/B978-012369542-0/50017-9

[cmi13108-bib-0040] Takenaka, M. , Zehrmann, A. , Brennicke, A. , & Graichen, K. (2013). Improved computational target site prediction for pentatricopeptide repeat RNA editing factors. PLoS ONE, 8(6), e65343 10.1371/journal.pone.0065343 23762347 PMC3675099

[cmi13108-bib-0041] Tarr, S. J. , Nisbet, R. E. R. , & Howe, C. J. (2011). Transcript‐level responses of *Plasmodium falciparum* to thiostrepton. Molecular and Biochemical Parasitology, 179(1), 37–41. 10.1016/j.molbiopara.2011.05.004 21620902

[cmi13108-bib-0042] Tonkin, C. J. , van Dooren, G. G. , Spurck, T. P. , Struck, N. S. , Good, R. T. , Handman, E. , … McFadden, G. I. (2004). Localization of organellar proteins in *Plasmodium falciparum* using a novel set of transfection vectors and a new immunofluorescence fixation method. Molecular and Biochemical Parasitology, 137(1), 13–21. 10.1016/j.molbiopara.2004.05.009 15279947

[cmi13108-bib-0043] Tourasse, N. J. , Choquet, Y. , & Vallon, O. (2013). PPR proteins of green algae. RNA Biology, 10(9), 1526–1542. 10.4161/rna.26127 24021981 PMC3858436

[cmi13108-bib-0044] Waller, R. F. , Reed, M. B. , Cowman, A. F. , & McFadden, G. I. (2000). Protein trafficking to the plastid of Plasmodium falciparum is via the secretory pathway. The EMBO Journal, 19(8), 1794–1802. 10.1093/emboj/19.8.1794 10775264 PMC302007

[cmi13108-bib-5648] Waterhouse, A. , Bertoni, M. , Bienert, S. , Studer, G. , Tauriello, G. , Gumienny, R. , & Lepore, R. (2018). SWISS‐MODEL: homology modelling of protein structures and complexes. Nucleic acids research, 46 W1, W296–W303. 10.1093/nar/gky427 PMC6030848 29788355

[cmi13108-bib-0045] Wilson, R. J. M. , Denny, P. W. , Preiser, P. R. , Rangachari, K. , Roberts, K. , Roy, A. , … Williamson, D. H. (1996). Complete gene map of the plastid‐like DNA of the malaria parasite *Plasmodium falciparum* . Journal of Molecular Biology, 261(2), 155–172. 10.1006/jmbi.1996.0449 8757284

[cmi13108-bib-0046] Yin, P. , Li, Q. , Yan, C. , Liu, Y. , Liu, J. , Yu, F. , … Yan, N. (2013). Structural basis for the modular recognition of single‐stranded RNA by PPR proteins. Nature, 504, 168–171. 10.1038/nature12651 24162847

[cmi13108-bib-0047] Zhang, M. , Wang, C. , Otto, T. D. , Oberstaller, J. , Liao, X. , Adapa, S. R. , … Adams, J. H. (2018). Uncovering the essential genes of the human malaria parasite *Plasmodium falciparum* by saturation mutagenesis. Science, 360(6388), eaap7847 10.1126/science.aap7847 29724925 PMC6360947

