# Supplementary figures and images for: An essential pentatricopeptide repeat protein in the apicomplexan remnant chloroplast
Source: Cell Microbiol. 2019 Sep 16;21(12):e13108. doi: 10.1111/cmi.13108 (PMC6899631; doi:10.1111/cmi.13108)

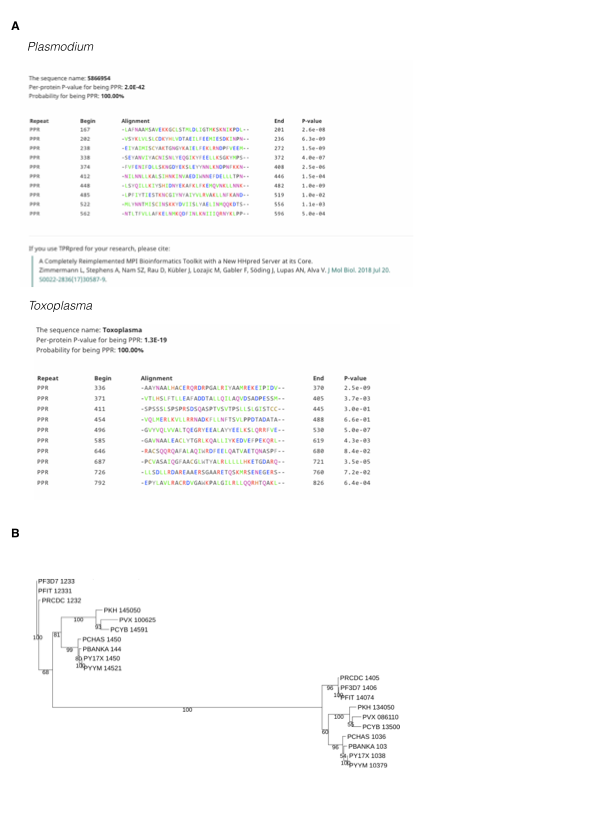

Supplement: Supplementary file 1 — Figure S1. PPR proteins in Plasmodium and Toxoplasma. A. PPR predictions for Plasmodium falciparum and Toxoplasma gondii PPR1. B. PPR1 and PPR2 proteins across the Plasmodium species. Mature (i.e. without targeting sequence) PPR1 (apicoplast) and PPR2 (mitochondrial) were aligned across selected Plasmodium species using ClustalW, and a phylogenetic tree inferred using PhyML. [file CMI-21-na-s001.tiff]

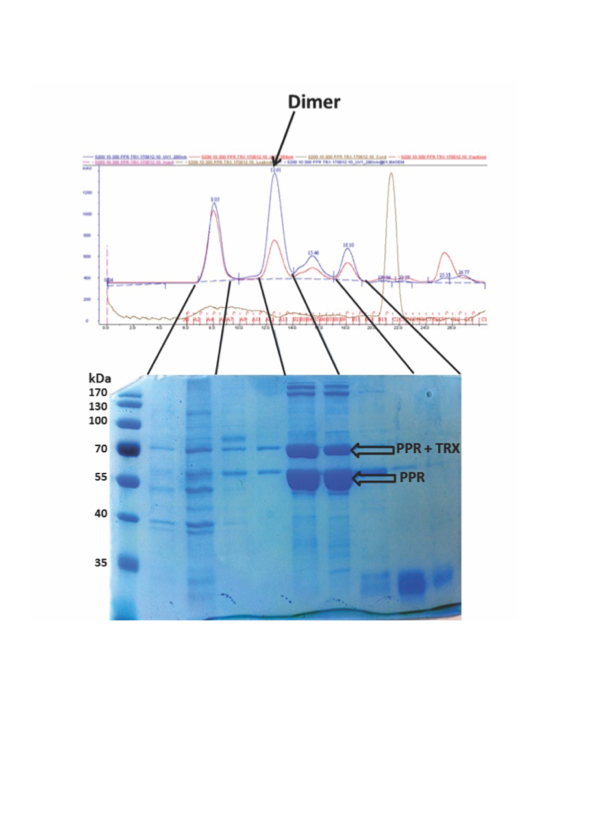

Supplement: Supplementary file 3 — Figure S3. Gel filtration chromatography of His 6 ‐TRX‐PfPPR1 and corresponding SDS‐PAGE gel. Blue line represents absorbance at 280 nm, red line absorbance at 260 nm and brown line conductivity. The trace for absorbance at 280 nm (blue line) shows four major peaks eluted from the S200 10/300 column. Based on calibration of the S200 10/300 column, the second peak corresponds to a molecular weight of approximately 135 kDa, and the estimated molecular weight of the His6‐TRX‐PfPPR1 dimer is 141.6 kDa. Spontaneous cleavage of the TRX‐Histag is observed in the second peak, corresponding to PPR protein without the His6‐TRX tag (estimated molecular weight of 56.8 kDa) with a smaller amount in the third peak. The fourth peak corresponds to the His6‐TRX tag only (14 kDa). [file CMI-21-na-s006.tiff]

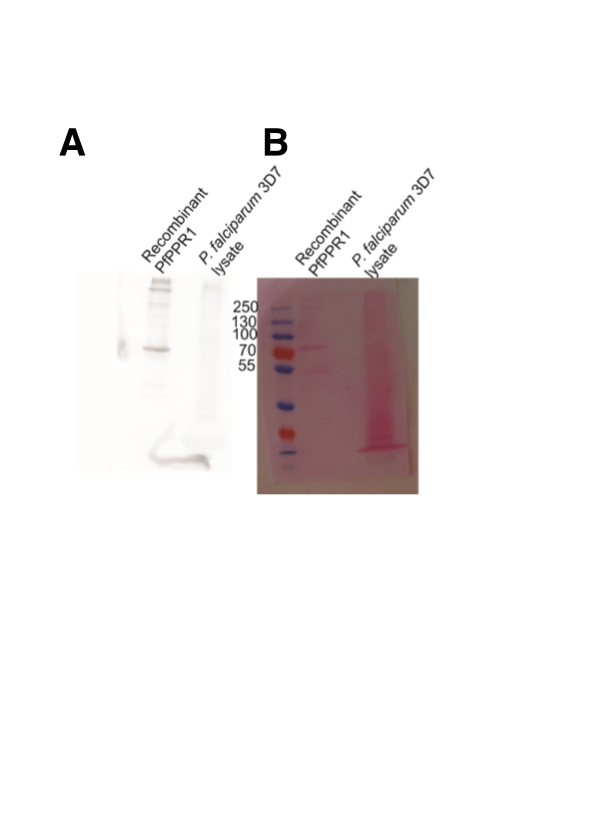

Supplement: Supplementary file 4 — Figure S4. Western blot of purified PfPPR1 protein and P. falciparum 3D7 lysate using the purified polyclonal anti‐PfPPR1 antibody. Purified PfPPR1 protein was run on 10% SDS‐PAGE gel along with P. falciparum 3D7 cell lysate. After incubation with the purified PfPPR1 polyclonal rabbit antibody and a secondary goat anti‐rabbit antibody conjugated to HRP, no PfPPR1 protein could be detected in the P. falciparum 3D7 lysate (Panel A).. The positive control (recombinant PfPPR1) shows a band of the correct size (PfPPR1 + TRX His6 ~ 72.8 kDa) .The ponceau stained gel (Panel B) shows good transfer of proteins of all molecular weights. Size markers are shown in kDa. [file CMI-21-na-s007.tiff]

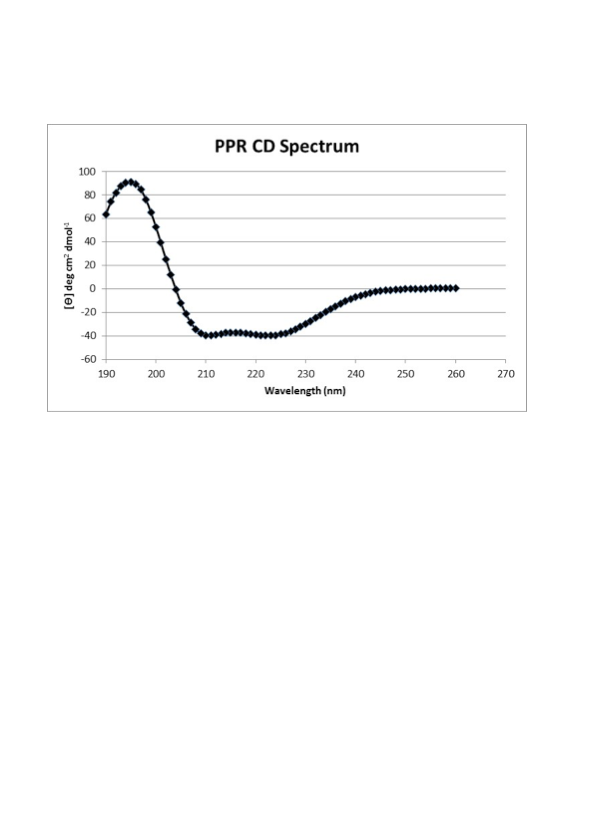

Supplement: Supplementary file 5 — Figure S5. Circular Dichroism (CD) spectrum of purified PfPPR1. PfPPR1 minus His‐TRX tag at 25°C in 10 mM potassium phosphate pH 8.0, 50 mM Na fluoride. Spectrum is typical of that for an alpha‐helical protein. [file CMI-21-na-s008.tiff]

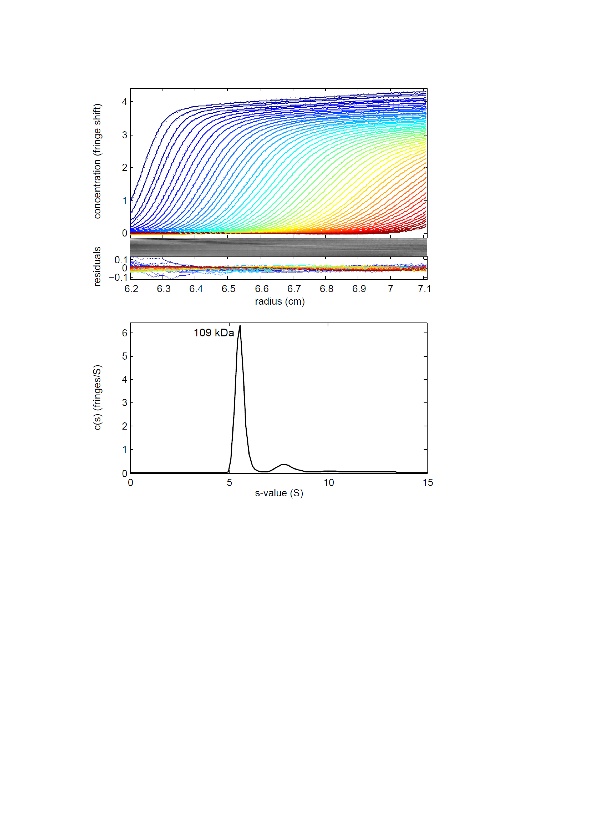

Supplement: Supplementary file 6 — Figure S6. Analytical ultracentrifugation (AUC) sedimentation velocity data for PfPPR1 minus His 6 ‐TRX tag. The residuals are from the fit with the continuous c(s) distribution model. Component sedimentation coefficient distribution for PPR at 1.8 mg/mL showing populations of dimeric (fitted mass of 109 kDa) and higher‐order species, fitting to a uniform frictional ratio of Fk,w = 1.378. The r.m.s.d. was 0.016. [file CMI-21-na-s009.tiff]

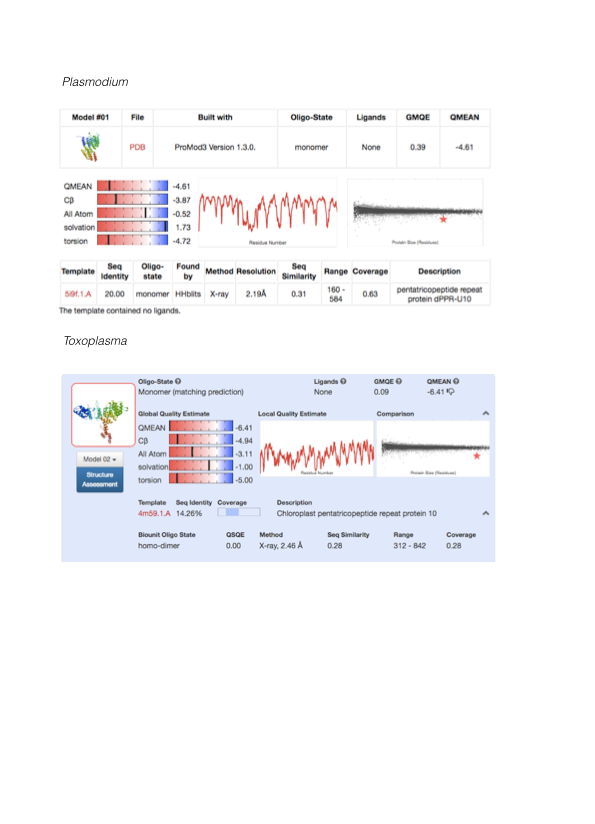

Supplement: Supplementary file 7 — Figure S7. SWISS‐MODEL analysis of PPR1 from both Toxoplasma and Plasmodium. [file CMI-21-na-s010.tiff]

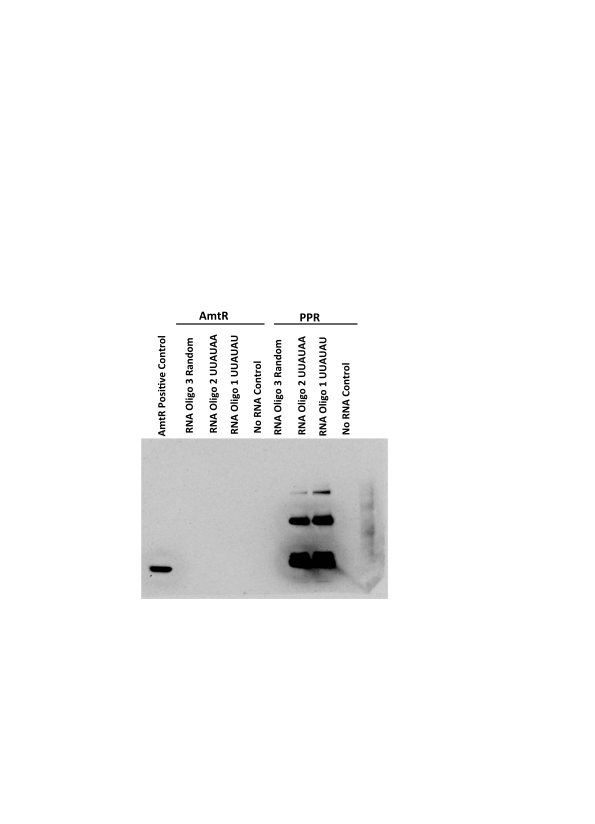

Supplement: Supplementary file 8 — Figure S8. PfPPR1 – RNA pull down assays. (A) Five 150 nt RNA molecules (RNAs 1‐5, sequences as shown in Figure 3) and apicoplast RNA transcripts (LSU rRNA – rpoB and tufA – clpC) were used in a pull‐down experiment. Biotinylated RNA was bound to streptavidin beads and used as ‘bait' to pull down PfPPR1 protein (with the TRX‐His6 tag removed). Bound PfPPR1 was protein detected using a purified polyclonal anti‐PfPPR1 antibody from rabbit. Loading controls (PPR1 only) and a no RNA control reaction showed no non‐specific binding in the absence of RNA. (B) The same pull down experiment using RNA oligonucleotides 1 – 3. The same result was obtained when the experiment was repeated (E). Details of RNA sequences are given in the legend to Figure 3, and specific sequences are shown (C and D). [file CMI-21-na-s011.tiff]

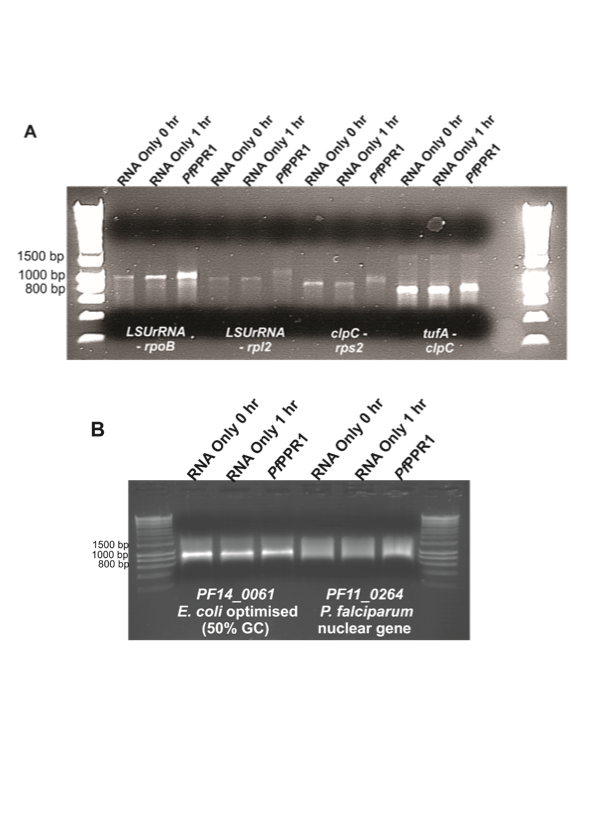

Supplement: Supplementary file 9 — Figure S9. PfPPR1 shows specific binding to apicoplast RNA transcripts. PfPPR1 binding to apicoplast RNA transcripts was tested in a gel shift experiment. Recombinant PfPPR1 (minus TRX‐His6) causes a shift in the migration of in vitro transcribed apicoplast RNA molecules following incubation for one hour (Panel A). No shift is seen in the RNA if it is not bound to PPR1. Panel B shows that no shift is seen when PPR1 is incubated with in vitro transcribed RNA from a nuclear encoded P. falciparum gene (PF11_0264, or from an E. coli codon‐optomized P. falciparum gene (PF14_0061) and a P. falciparum 3D7 nuclear gene (PF11_0264). [file CMI-21-na-s012.tiff]

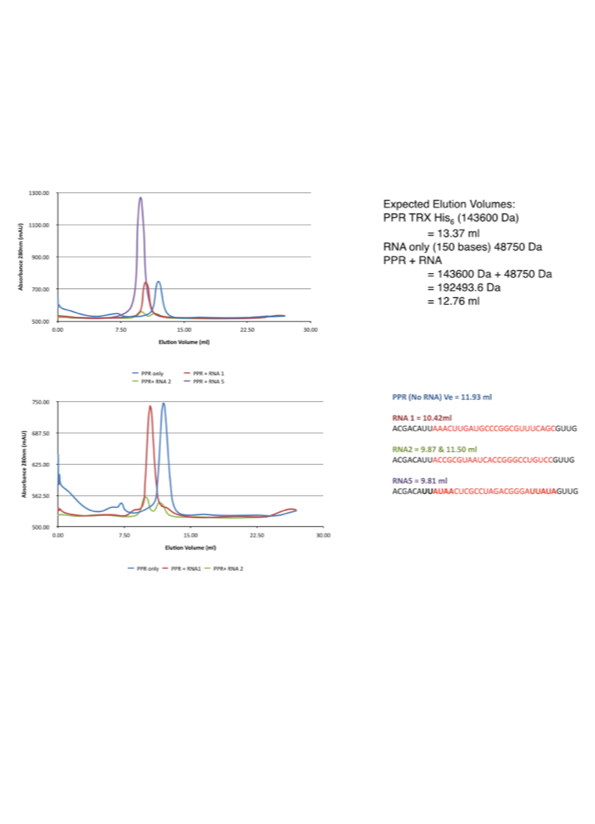

Supplement: Supplementary file 10 — Figure S10 Gel filtration shows a change in elution profile when PfPPR1 is bound to RNA. This is a repeat of the data shown in Figure 4, showing that the change in mobility following gel filtration is reproducible. [file CMI-21-na-s002.tiff]

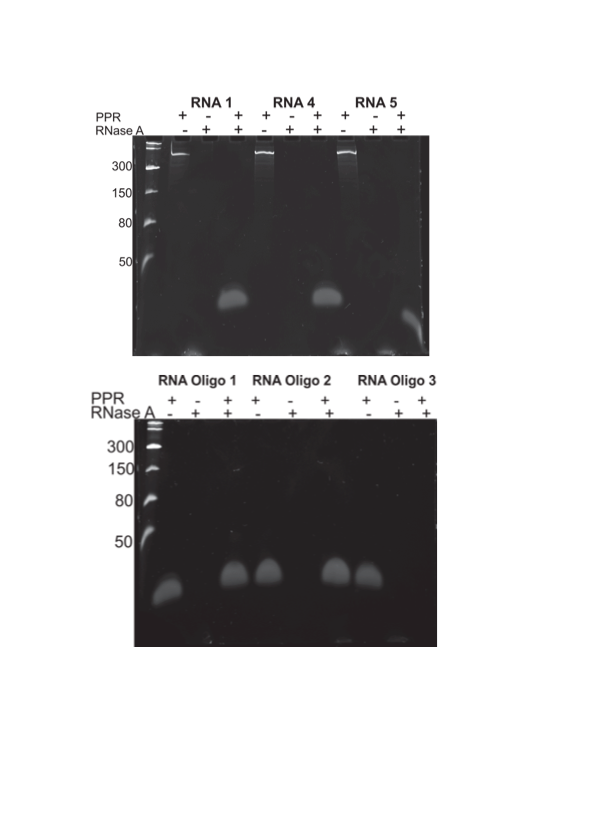

Supplement: Supplementary file 11 — Figure S11. Ribonuclease A protection assays. RNA transcripts 1, 4 and 5 and RNA oligos 1,2 and 3 were incubated in a 1:1 molar ratio with PfPPR1 prior to treatment with RNase A. Samples were analyzed using a native acrylamide gel (ladder in nt). Experiments with no PfPPR1 bound to RNA showed complete degradation by RNase A [file CMI-21-na-s003.tiff]
