# Supplementary material for: An essential pentatricopeptide repeat protein in the apicomplexan remnant chloroplast
Source: Cell Microbiol. 2019 Sep 16;21(12):e13108. doi: 10.1111/cmi.13108 (PMC6899631; doi:10.1111/cmi.13108)
Supplement: Supplementary file 2 — Table S1. Predicted apicoplast localization. Table S2. Conditions trialled for detection of PfPPR1 in P. falciparum 3D7 lysate by Western Blot Table S3. Primers used in this study Figure S2. Alignment of PPR1 proteins from Plasmodium, Toxoplasma and PPR10 from Arabidopsis thaliana. Plasmodium species as in Supplemental Figure 1. [file CMI-21-na-s005.docx]

**Supplemental Table S1. Predicted apicoplast localization.**

|  |  | **PlasmoAP Prediction** | | | | | **PlasMit Prediction** | |
| --- | --- | --- | --- | --- | --- | --- | --- | --- |
| **Protein ID** | **Species** | **Signal Peptide Value** | **Decision** | **Apicoplast Targeting Peptide Value** | **Decision** | **Targeted to the Apicoplast** | **Jury** | **Strict** |
| PF14_  0061 | *Plasmodium falciparum 3D7* | 3/4 | + | 5/5 | ++ | YES | 99% non -mito | n/a |
| PFTANZ_05018 | *Plasmodium falciparum Tanzania* | 3/4 | + | 5/5 | ++ | YES | 99% non -mito | n/a |
| PFFVO_  04677 | *Plasmodium falciparum Vietnam Oak-Knoll* | 3/4 | + | 5/5 | ++ | YES | 99% non -mito | n/a |
| PRCDC_1405700 | *Plasmodium reichenowi* | 2/4 | 0 | 5/5 | ++ | NO? | 99% non -mito | n/a |
| PKH_  13050 | *Plasmodium knowlesi strain H* | 4/4 | ++ | 5/5 | ++ | YES | 99% non -mito | n/a |
| PVX_  086110 | *Plasmodium vivax Sal1* | 3/4 | + | 3/5 | - | NO? | mito 91% | failed |
| C922_  03803 | *Plasmodium inui San Antonio I* | 0/4 | - | 2/5 | - | NO | 99% non-mito | n/a |
| PYO3222 | *Plasmodium yoelii 17XNL* | 3/4 | + | 5/5 | ++ | YES | 99% non-mito | n/a |
| PY17X_  1038200 | *Plasmodium yoelii 17X* | 3/4 | + | 5/5 | ++ | YES | 99% non-mito | n/a |
| YYE_  02192 | *Plasmodium vinckei* | 4/4 | ++ | 5/5 | ++ | YES | 99% non-mito | n/a |
| PBANKA_103580 | *Plasmodium berghei ANKA* | 4/4 | ++ | 5/5 | ++ | YES | 99% non-mito | n/a |
| PCHAS_103660 | *Plasmodium chaubadi* | 4/4 | ++ | 5/5 | ++ | YES | 99% non-mito | n/a |
| PCYB_  135000 | *Plasmodium cynmolgi Strain B* | 0/4 | - | 0/5 | - | NO | 99% non-mito | n/a |
| PFIT_  1407400 | *Plasmodium falciparum IT* | 3/4 | + | 5/5 | ++ | YES | 99% non-mito | n/a |

**Supplemental Table S2. Conditions trialled for detection of PfPPR1 *in P. falciparum 3D7* lysate by Western Blot**

| **Problem** | **Conditions trialled** |
| --- | --- |
| Transfer efficiency | iBLOT (dry transfer), wet transfer, temperature of wet transfer. Checked transfer efficiency by staining the membrane with Ponceau and by staining the gel post-transfer with coomassie blue. Wet transfer at room temperature best. |
| Background in western blot images | Tested preimmune and postimmune serum, background also present in preimmune serum |
| Blocking solution to decrease background | 1, 2 and 5% (w/v) BSA and 5 and 10% (w/v) non-fat milk powder tested. 10% skim milk powder lowest background. |
| Increase specificity | Included 0.5 M NaCl in 1xTBS + tween washes. Did not make any difference. |
| Primary antibody detection | Purified primary antibody. Compared different dilutions of purified and non-purified primary antibody (1:200, 1:500, 1:1000, 1:2000). Decided upon 1:1000 dilution |
| Secondary antibody detection | Tested for specificity of anti-rabbit AlexaFluor-568 antibody by performing a western blot on purified PPR and plasmodium lysate with no primary antibody. No non-specific binding observed. |

**Supplemental Table S3. Primers used in this study**

| **Primer** | **Sequence** |
| --- | --- |
| clpC FWD | CCTTTATATGGAGCTCGT |
| clpC FWD + T7 | GCCATAATACGACTCACTATAGCCTTTATATGGAGCTCGT |
| clpC REV | CTGATATTTCTTTTAATTTTATTC |
| *KDPPR-Fwd* | GTCTGACAGTCTGTCTCTATGGTGCGCCCGTTGTCGAGACAACTCTGTC |
| *KDPPR-Rev* | GGAAAGAGAAGAGAGAAGGACGGCGCATGCTAGCAGATCTGGTTGAAGAC |
| LSU rRNA + T7 FWD | GCCATAATACGACTCACTATAG GTGTATAATTCCTAATAAGTTGA |
| LSU rRNA FWD | GTGTATAATTCCTAATAAGTTGA |
| LSUrRNA + T7 FWD | GCCATAATACGACTCACTATAGGTGTATAATTCCTAATAAGTTGA |
| LSUrRNA FWD | GTGTATAATTCCTAATAAGTTGA |
| PF11_0264 minus leader + T7 FWD | GCCATAATACGACTCACTATAGAAGGACACACATATTATTGAT |
| PF11_0264 minus leader FWD | AAGGACACACATATTATTGATAAAAAG |
| PF11_0264 REV | TCAGCTAAAAAAGTAAAGACTATCC |
| PF14_0061 minus leader +T7 FWD | GCCATAATACGACTCACTATAGGACAACTTTCTGAACG |
| PF14_0061 minus leader FWD | GACAACTTTCTGAACG |
| PF14_0061 REV | ATAGTTTTCGGTTTCGG |
| PF14_0061_Nterm Fwd | AAGTTCTGTTTCAGGGCCCGGACAACTTTCTGAACG |
| PF14_0061_Nterm  Rev | ATGGTCTAGAAAGCTTTAATAGTTTTCGGTTTCGG |
| pGEM FWD | TGCAAGGCGATTAAGTTGGGT |
| pGEM REV | TGTGGAATTGTGAGCGGATAAC |
| rpl2 REV | TCCACCTCCTTTATTATAAATAG |
| rpoB REV | GAATACATGTTTTATATAATCC |
| rps2 REV | TATGAACCAATTATTTTAGGTA |
| tufA + T7 FWD | GCCATAATACGACTCACTATAGAATGTACAAAAAGTAGCTATACCT |
| tufA FWD | AATGTACAAAAAGTAGCTATACCT |

**Supplemental Figure S1.** PPR proteins in *Plasmodium* and *Toxoplasma.* A. PPR predictions for *Plasmodium falciparum*  and *Toxoplasma*  *gondii* PPR1. B. PPR1 and PPR2 proteins across the *Plasmodium* species*.* Mature (i.e. without targeting sequence) PPR1 (apicoplast) and PPR2 (mitochondrial) were aligned across selected *Plasmodium* species using ClustalW, and a phylogenetic tree inferred using PhyML.

Key:

PF3D7: *P. falciparum 3D7* (PF14_0061 and PF3D7_1233300),

PFIT: *P. falciparum* IT (PFIT_1407400 and [PfIT_120038300](http://plasmodb.org/plasmo/app/record/gene/PfIT_120038300)),

PRCDC: *P. reichenowi*, ([PRCDC_1232600](http://plasmodb.org/plasmo/app/record/gene/PRCDC_1232600) and [PRCDC_1405700](http://plasmodb.org/plasmo/app/record/gene/PRCDC_1405700)),

PKH: *P. knowlesi* strain H (PKH_13050 and [PKNH_1452800](http://plasmodb.org/plasmo/app/record/gene/PKNH_1452800)),

PVH: *P. vivax (*PVX_086110 and [PVX_100625](http://plasmodb.org/plasmo/app/record/gene/PVX_100625))*,*

PCYB: *P. cynmolgi* Strain B (PCYB_135000 and [PCYB_145910](http://plasmodb.org/plasmo/app/record/gene/PCYB_145910)),

PBANKA: *P.berghei* ANKA (PBANKA_103580 and [PBANKA_1448000](http://plasmodb.org/plasmo/app/record/gene/PBANKA_1448000)),

PY17X *P. yoelii yoelii* 17X (PY17X_1038200 and [PY17X_1450500](http://plasmodb.org/plasmo/app/record/gene/PY17X_1450500)),

PYYM: *P. yoelii yoelii* YM (PYYM_1037900­ and [PYYM_1452100](http://plasmodb.org/plasmo/app/record/gene/PYYM_1452100)).

**Supplemental Figure 2.** Alignment of PPR1 proteins from *Plasmodium*, *Toxoplasma* and PPR10 from *Arabidopsis* *thaliana*. *Plasmodium* species as in Supplemental Figure 1.

AthPPR10 ------------------------------------------------------------ 0

Toxoplasma HFAFASFEKAKGSEKCEPAKTPRPTVETADGEPLRRDPADPSSGSARVCGVHTSGAPLAT 60

PVX_086110 ------------------------------------------------------------ 0

PKH_134050 ------------------------------------------------------------ 0

C922_03803 ------------------------------------------------------------ 0

PCYB_135000 ------------------------------------------------------------ 0

PRCDC_1405700 ------------------------------------------------------------ 0

PF3D7_1406400 ------------------------------------------------------------ 0

PFFVO_05018 ------------------------------------------------------------ 0

PFIT_1407400 ------------------------------------------------------------ 0

PFTANZ_05018 ------------------------------------------------------------ 0

PCHAS_103660 ------------------------------------------------------------ 0

PY17X_1038200 ------------------------------------------------------------ 0

PYO3222 ------------------------------------------------------------ 0

PYYM_1037900 ------------------------------------------------------------ 0

PBANKA_103580 ------------------------------------------------------------ 0

AthPPR10 ------------------------------------------------------------ 0

Toxoplasma VPKANNESCEGKRHFRFRTNDEEGNHGNELEGRFEEPEEPEEPDRAAEGQEKRRRENSGK 120

PVX_086110 ------------------------------------------------------------ 0

PKH_134050 ------------------------------------------------------------ 0

C922_03803 ------------------------------------------------------------ 0

PCYB_135000 ------------------------------------------------------------ 0

PRCDC_1405700 ------------------------------------------------------------ 0

PF3D7_1406400 ------------------------------------------------------------ 0

PFFVO_05018 ------------------------------------------------------------ 0

PFIT_1407400 ------------------------------------------------------------ 0

PFTANZ_05018 ------------------------------------------------------------ 0

PCHAS_103660 ------------------------------------------------------------ 0

PY17X_1038200 ------------------------------------------------------------ 0

PYO3222 ------------------------------------------------------------ 0

PYYM_1037900 ------------------------------------------------------------ 0

PBANKA_103580 ------------------------------------------------------------ 0

AthPPR10 ------------------------------------------------------------ 0

Toxoplasma TPGRATGKRKSLNGYHASWLASAFNRHQASHIHSLRVSSFRSPLSSFLSSSHSSSLALCS 180

PVX_086110 ------------------------------------------------------------ 0

PKH_134050 ------------------------------------------------------------ 0

C922_03803 ------------------------------------------------------------ 0

PCYB_135000 ------------------------------------------------------------ 0

PRCDC_1405700 ------------------------------------------------------------ 0

PF3D7_1406400 ------------------------------------------------------------ 0

PFFVO_05018 ------------------------------------------------------------ 0

PFIT_1407400 ------------------------------------------------------------ 0

PFTANZ_05018 ------------------------------------------------------------ 0

PCHAS_103660 ------------------------------------------------------------ 0

PY17X_1038200 ------------------------------------------------------------ 0

PYO3222 ------------------------------------------------------------ 0

PYYM_1037900 ------------------------------------------------------------ 0

PBANKA_103580 ------------------------------------------------------------ 0

AthPPR10 --------------------------------------------------ASRRDELLRA 10

Toxoplasma SSFSPFASSLLSPSPLFLNPASSASGRRVSLSNFLSSPSSSSPSVSASSSCSSSSSLLRA 240

PVX_086110 ------------------------------------------------------------ 0

PKH_134050 ------------------------------------------------------------ 0

C922_03803 ------------------------------------------------------------ 0

PCYB_135000 ------------------------------------------------------------ 0

PRCDC_1405700 ------------------------------------------------------------ 0

PF3D7_1406400 ------------------------------------------------------------ 0

PFFVO_05018 ------------------------------------------------------------ 0

PFIT_1407400 ------------------------------------------------------------ 0

PFTANZ_05018 ------------------------------------------------------------ 0

PCHAS_103660 ------------------------------------------------------------ 0

PY17X_1038200 ------------------------------------------------------------ 0

PYO3222 ------------------------------------------------------------ 0

PYYM_1037900 ------------------------------------------------------------ 0

PBANKA_103580 ------------------------------------------------------------ 0

AthPPR10 D----------------------------------------ITSLLKALELSGHWEWALA 30

Toxoplasma LVACHSSPQTASVSLSPPRGSSFSSLSPSVLLRRSETRTQAPTLQAPLLSVRGS------ 294

PVX_086110 -----------------------------------------------DSPPDGD------ 7

PKH_134050 -----------------------------------------------AAIHDED------ 7

C922_03803 -----------------------------------------------TATPDED------ 7

PCYB_135000 -----------------------------------------------GATPDED------ 7

PRCDC_1405700 -----------------------------------------------DNFLNEN------ 7

PF3D7_1406400 -----------------------------------------------DNFLNEN------ 7

PFFVO_05018 -----------------------------------------------DNFLNEN------ 7

PFIT_1407400 -----------------------------------------------DNFLNEN------ 7

PFTANZ_05018 -----------------------------------------------DNFLNEN------ 7

PCHAS_103660 ------------------------------------------------NQNDDS------ 6

PY17X_1038200 ------------------------------------------------NKNDDN------ 6

PYO3222 ------------------------------------------------NKNDDN------ 6

PYYM_1037900 ------------------------------------------------NKNDDN------ 6

PBANKA_103580 ------------------------------------------------NRNDD------- 5

AthPPR10 LLRWAGKEGAADASALEMVVRALGREGQHDAVCALLDETPLPPGSRLDVRAYTTVLHALS 90

Toxoplasma ---WPPDAGDDRET--EKRLSPLRI--DYRQAFE-LLENADPTGIKALTAAYNAALHACE 346

PVX_086110 ---ILKRKGAEKEV--EKSVVPLNM--DWVKVMN-LIYAS--RDVDATTLAFNAAMSAVE 57

PKH_134050 ---ILRKKGAQKEV--EKSVVPLNM--DWVKVMN-LIYAS--RDVDATTLAFNAAMSAVE 57

C922_03803 ---ILKKKGAQKEV--EQSVVPLNM--DWVKVMN-LIYAS--RDVDATTLAFNAAMSAVE 57

PCYB_135000 ---ILKKKGAQKEV--EKSVVPLNM--DWVKVMN-LIYAS--RDVDATTLAFNAAMSAVE 57

PRCDC_1405700 ---ILKKKSSEKEI--EQSLTPLNM--DWVKVMN-LIYSS--NDIDATTLAFNAAMSAVE 57

PF3D7_1406400 ---ILKKKSSEKEI--EQSLTPLNM--DWVKVMN-LIYSS--NDIDATTLAFNAAMSAVE 57

PFFVO_05018 ---ILKKKSSEKEI--EQSLTPLNM--DWVKVMN-LIYSS--NDIDATTLAFNAAMSAVE 57

PFIT_1407400 ---ILKKKSSEKEI--EQSLTPLNM--DWVKVMN-LIYSS--NDIDATTLAFNAAMSAVE 57

PFTANZ_05018 ---ILKKKSSEKEI--EQSLTPLNM--DWVKVMN-LIYSS--NDIDATTLAFNAAMSAVE 57

PCHAS_103660 ---ILKKKHLEKEI--EQSVIPLNM--DWVKVMN-LISSS--SDVNTTTLAFNAALSAVE 56

PY17X_1038200 ---ILKKKHSEKQI--EQSIIPLNI--DWIKVMN-LISSS--NDVNTTTLAFNAALSAVE 56

PYO3222 ---ILKKKHSEKQI--EQSIIPLNI--DWIKVMN-LISSS--NDVNTTTLAFNAALSAVE 56

PYYM_1037900 ---ILKKKHSEKQI--EQSIIPLNI--DWIKVMN-LISSS--NDVNTTTLAFNAALSAVE 56

PBANKA_103580 ---ILKKKYSEKEI--EQSIIPLNM--DWVKVMN-LISAS--NDVNTTTLAFNAALSAVE 55

* : * : . * . . *:.:.: * .

AthPPR10 RAGRYERALELFAELRRQGVAPTLVTYNVVLDVYGRMGRSWPRIVALLDEMR-AAGVEPD 149

Toxoplasma RQRDRPGALRIYAAMREKEIPIDVVTLHSLFTLLEAFADDTA----LLQILAQVDSADPE 402

PVX_086110 KKGCLKSMLEVFEIMKKKNIKPDLVSYKLLLRLCANYHLGEHAEI-LFDEMVETDKLTPT 116

PKH_134050 KKGCLKSMLELFEVMKKKNIKPDLVSYKLLLRLCANYHLGDHAEI-LFDEMVETDKLTPT 116

C922_03803 KKGCLKSMLELFEIMKKKNIKPDLVSYKLLLRLCANYHLGEHAEI-LFDEMVETEKLTPT 116

PCYB_135000 KKGCLKSMLELFEIMKKKNIKPDLVSYKLLLRLCANYHLGEHAEI-LFDEMVETDKLTPT 116

PRCDC_1405700 KKGCLTTMLDLIGTMKSKNIKPDLVSYKLVLSLCDKYHLVDTAEI-LFEEMIESDKINPN 116

PF3D7_1406400 KKGCLSTMLDLIGTMKSKNIKPDLVSYKLVLSLCDKYHLVDTAEI-LFEEMIESDKINPN 116

PFFVO_05018 KKGCLSTMLDLIGTMKSKNIKPDLVSYKLVLSLCDKYHLVDTAEI-LFEEMIESDKINPN 116

PFIT_1407400 KKGCLSTMLDLIGTMKSKNIKPDLVSYKLVLSLCDKYHLVDTAEI-LFEEMIESDKINPN 116

PFTANZ_05018 KKGCLSTMLDLIGTMKSKNIKPDLVSYKLVLSLCDKYHLVDTAEI-LFEEMIESDKINPN 116

PCHAS_103660 KKGCLTSIIELFEIMKKKNIKPDLISYKLILTLCDKYHLAEYAEI-LFDEMTESDNIRPN 115

PY17X_1038200 KKGCLTSIIELFEIMKKKNIKPDLISYKLILSLCDKYHLAEYAEI-LFDEMVESDNIRPS 115

PYO3222 KKGCLTSIIELFEIMKKKNIKPDLISYKLILSLCDKYHLAEYAEI-LFDEMVESDNIRPS 115

PYYM_1037900 KKGCLTSIIELFEIMKKKNIKPDLISYKLILSLCDKYHLAEYAEI-LFDEMVESDNIRPS 115

PBANKA_103580 KKRCLTSIIELFEIMKKKNIKPDLISYKLILTLCDKYHLADYAEI-LFNEMVESDNIRPN 114

: : : :: : : ::: : :: : *:: : *

AthPPR10 -------------------------------GFTASTVIAACCRDGLVDEAVAFFEDLKA 178

Toxoplasma SSMAFLSRSPSSSLSPSPRSDSQASPTVSVTPSLLSLGISTCCRAGNATAAVQLMERLKV 462

PVX_086110 -------------------------------YEIYALMISCFAKVGDGHRAVEFVEKLRS 145

PKH_134050 -------------------------------YEIYALMINCFAKVGDGHKAVEFLEKLRS 145

C922_03803 -------------------------------YEIYALMISCFAKVGDGHKAVEFLEKLRS 145

PCYB_135000 -------------------------------YEIYALMISCFAKVGDGHKAVEFVEKLRS 145

PRCDC_1405700 -------------------------------YEIYAIMISCYAKTGNGYKAIELFEKLRN 145

PF3D7_1406400 -------------------------------YEIYAIMISCYAKTGNGYKAIELFEKLRN 145

PFFVO_05018 -------------------------------YEIYAIMISCYAKTGNGYKAIELFEKLRN 145

PFIT_1407400 -------------------------------YEIYAIMISCYAKTGNGYKAIELFEKLRN 145

PFTANZ_05018 -------------------------------YEIYAIMISCYAKTGNGYKAIELFEKLRN 145

PCHAS_103660 -------------------------------YEIYSIMISCFSKVGDGHKAIEFLEKLRN 144

PY17X_1038200 -------------------------------YEIYSIMISCFSKVGDGHKAIEFLEKLRN 144

PYO3222 -------------------------------YEIYSIMISCFSKVGDGHKAIEFLEKLRN 144

PYYM_1037900 -------------------------------YEIYSIMISCFSKVGDGHKAIEFLEKLRN 144

PBANKA_103580 -------------------------------YEIYSIMISCFSKVGDGYKASEFLEKLRN 143

: * .: * * :.* *:

AthPPR10 RG---HAPCVVTYNALLQVFGKAGNYTEALRV--LGEMEQNG------------------ 215

Toxoplasma LLRRNADKFLLNFTSVLPPDTADA------------------------------------ 486

PVX_086110 D------PLVGEVNNWG-EAGG---------------------VSGSGSADD-------- 169

PKH_134050 D------PLVEGVNNWEVDGSGDGNGSAGRNVSNDGNVSNDGNVSDDGNVSDDGNVNRGE 199

C922_03803 D------PLVEEVNNWG-DRSGD--------------------TADRGDTAD-----RGD 173

PCYB_135000 D------PLVEEVNNWGGDSSGVGDSSGG------GDSSGGGDRSGAGDSSGDG---DGS 190

PRCDC_1405700 D------PLVEEMRSLNITNSNDN-------------------KENSNDLET-------- 172

PF3D7_1406400 D------PFVEEMRSLNITNTNDN-------------------KENSNDLQT-------- 172

PFFVO_05018 D------PFVEEMRSLNITNTNDN-------------------KENSNDLQT-------- 172

PFIT_1407400 D------PFVEEMRSLNITNTNDN-------------------KENSNDLQT-------- 172

PFTANZ_05018 D------PFVEEMRSLNITNTNDN-------------------KENSNDLQT-------- 172

PCHAS_103660 D------PFVENFKEL---TSKYD-------------------KEKSNRWED-------- 168

PY17X_1038200 D------PFVENIKEL---DSKYD-------------------KEKSNRWEN-------- 168

PYO3222 D------PFVENIKEL---DSKYD-------------------KEKSNRWEN-------- 168

PYYM_1037900 D------PFVENIKEL---DSKYD-------------------KEKSNRWEN-------- 168

PBANKA_103580 D------PFVENIKEL---NSKYD-------------------KEKSNRWDN-------- 167

:

AthPPR10 -------------------------------------------CQPDAVTYNELAGTYA- 231

Toxoplasma ------------------------------------------------------------ 486

PVX_086110 RQHPAWRDSFAEEATTEEGAEAEGSTYTNQFKEITKKIKHIERGSS-KIQYSEYANVIFA 228

PKH_134050 RGNYLWKDHFDEQAATEEDAKAEGNAYTNQFKDITKKIKHIEKGSS-KIQYSEYTNVIFA 258

C922_03803 GGNPAWKDAFAEEAPTEEGAKAEGKTYTNEFKEITKKIKQIEKGSS-KIQYSEYTNVIFA 232

PCYB_135000 GENRVWKDAFAEEAATEEGAKEEGNNYTNQFKEITKKIKHIEKGSS-KIQFSEYTNVIFA 249

PRCDC_1405700 --SIIHNNIEDNNNN-NNNN---NNIYDDKFKHISNKIKNVENYSG-KIQYSEYANVIYA 225

PF3D7_1406400 --SIIHNNMEDNNNN-NNNN--DNNIYDDKFKHISNKIKNVENCSG-KIQYSEYANVIYA 226

PFFVO_05018 --SIIHNNMEDNNNN-NNNNNNDNNIYDDKFKHISNKIKNVENCSG-KIQYSEYANVIYA 228

PFIT_1407400 --SIIHNNMEDNNNN-NNNNNNDNNIYDDKFKHISNKIKNVENCSG-KIQYSEYANVIYA 228

PFTANZ_05018 --SIIHNNMEDNNNN-NNNNN-DNNIYDDKFKHISNKIKNVENCSG-KIQYSEYANVIYA 227

PCHAS_103660 --TFAQVNS-------NNGEDNTPNYYNAQFKELTEKIKNVENNNN-KIQYSEYANVIFA 218

PY17X_1038200 --TFAQVNSKNDDNKINNGEDDSPNYYNTQFKELTEKIKNVENNNNNKIQYSEYANVIFA 226

PYO3222 --TFAQVNSKNDDNKINNGEDDSPNYYNTQFKELTEKIKNVENNNNNKIQYSEYANVIFA 226

PYYM_1037900 --TFAQVNSKNDDNKINNGEDDSPNYYNTQFKELTEKIKNVENNNNNKIQYSEYANVIFA 226

PBANKA_103580 --TFAQVKSKSEDNKINHGEDDSPNYYNTQFKELTEKIKNVENNN--KIQYSEYANVIFA 223

AthPPR10 --RAGFFEEAARCLD-TMASKGLLPNAFTYNTVMTAYGNVGKVDEALALFDQMKKTGFVP 288

Toxoplasma ------------------TAAVAHPPTGVYVQLVVALTQEGRYEEALAYYEELKSLQRRF 528

PVX_086110 CNMSNLPEQGIKYFEELLNTGKYMPSTLLLESIFDLLAKNGNYEKCLDYYNKLKDDPNFK 288

PKH_134050 CNMSNLPEQGIKYFEELLNTGKYMPSTLLLESIFDLLAKNGKYEKCLDYYNKLKDDPNFK 318

C922_03803 CNMSNLPEQGIKYFEELLNTGKYMPSTLLLESIFDLLAKNGNYEKCLEYYNKLKDDPNFK 292

PCYB_135000 CNMSNLPEQGIKYFEELLNTGKYMPSTLLLESIFDLLAKNGKYEKSLEYYNKLKDDPNFK 309

PRCDC_1405700 CNISNLYEQGIKYFEELLKSGKYMPSIFVFENIFDLLSKNGDYEKSLEYYNNLKNDPNFK 285

PF3D7_1406400 CNISNLYEQGIKYFEELLKSGKYMPSIFVFENIFDLLSKNGDYEKSLEYYNNLKNDPNFK 286

PFFVO_05018 CNISNLYEQGIKYFEELLKSGKYMPSIFVFENIFDLLSKNGDYEKSLEYYNNLKNDPNFK 288

PFIT_1407400 CNISNLYEQGIKYFEELLKSGKYMPSIFVFENIFDLLSKNGDYEKSLEYYNNLKNDPNFK 288

PFTANZ_05018 CNISNLYEQGIKYFEELLKSGKYMPSIFVFENIFDLLSKNGDYEKSLEYYNNLKNDPNFK 287

PCHAS_103660 CNISNLHEQGIKYFEELLNSTKYIPSTFIFENIFDLLGKNGNYEKALDYYNKIKDDPNFK 278

PY17X_1038200 CNMSNLHEHGIKYFEELLNSTKYIPSTFIFENIFNLLGKNGNYEKALDYYNKIKDDPNFK 286

PYO3222 CNMSNLHEHGIKYFEELLNSTKYIPSTFIFENIFNLLGKNGNYEKALDYYNKIKDDPNFK 286

PYYM_1037900 CNMSNLHEHGIKYFEELLNSTKYIPSTFIFENIFNLLGKNGNYEKALDYYNKIKDDPNFK 286

PBANKA_103580 CNISNLHEHGIKYFEELLNSTKYIPSTFIFENIFDLLGKNGNYEKALDYYNKIKDDPNFK 283

: * :. : * ::.* ::::*.

AthPPR10 NVNTYNLVLGMLGKKSRF----------TVMLEMLGEMSRSGCTPNRVTWNTMLAVCGKR 338

Toxoplasma VEK-QQLLEREVARRAELVERELKNAPEQERKKLLGDIERQV------------------ 569

PVX_086110 -KA-I------------------------------------------------------- 291

PKH_134050 -KY-I------------------------------------------------------- 321

C922_03803 -KY-I------------------------------------------------------- 295

PCYB_135000 -KY-I------------------------------------------------------- 312

PRCDC_1405700 -KN-I------------------------------------------------------- 288

PF3D7_1406400 -KN-I------------------------------------------------------- 289

PFFVO_05018 -KN-I------------------------------------------------------- 291

PFIT_1407400 -KN-I------------------------------------------------------- 291

PFTANZ_05018 -KN-I------------------------------------------------------- 290

PCHAS_103660 -KY-I------------------------------------------------------- 281

PY17X_1038200 -KY-I------------------------------------------------------- 289

PYO3222 -KY-I------------------------------------------------------- 289

PYYM_1037900 -KY-I------------------------------------------------------- 289

PBANKA_103580 -KY-I------------------------------------------------------- 286

AthPPR10 GMEDYVTRVLEGMRSCGVELSRDTYNTLIAAYGRCGSRTNAFKMYNEMTSAGFTPCITTY 398

Toxoplasma ------------------------------------------VEEQELLLEDYQPPIGAV 587

PVX_086110 -------------------------------------------------------NVNIL 296

PKH_134050 -------------------------------------------------------NVNIL 326

C922_03803 -------------------------------------------------------NVNIL 300

PCYB_135000 -------------------------------------------------------NVNIL 317

PRCDC_1405700 -------------------------------------------------------NVNIL 293

PF3D7_1406400 -------------------------------------------------------NVNIL 294

PFFVO_05018 -------------------------------------------------------NVNIL 296

PFIT_1407400 -------------------------------------------------------NVNIL 296

PFTANZ_05018 -------------------------------------------------------NVNIL 295

PCHAS_103660 -------------------------------------------------------NVNIL 286

PY17X_1038200 -------------------------------------------------------NVNIL 294

PYO3222 -------------------------------------------------------NVNIL 294

PYYM_1037900 -------------------------------------------------------NVNIL 294

PBANKA_103580 -------------------------------------------------------NVNIL 291

:

AthPPR10 NALLNVLSRQGDWSTAQSIVSKMRTKGFKPNEQSYSLLLQCYAKGGNVAGIAAIENEVYG 458

Toxoplasma NAALEACLYTGRLKQALLIYK--------------------------------------- 608

PVX_086110 NNILKALSVQGKINIIEDLWK--------------------------------------- 317

PKH_134050 NNILKALCVHGKINIIEQVWK--------------------------------------- 347

C922_03803 NNILKALSVHAKSNIIEDVWK--------------------------------------- 321

PCYB_135000 NNILKALTVHGKINIIEQVWK--------------------------------------- 338

PRCDC_1405700 NNLLKTLSIHNKINVAEDIWN--------------------------------------- 314

PF3D7_1406400 NNLLKALSIHNKINVAEDIWN--------------------------------------- 315

PFFVO_05018 NNLLKALSIHNKINVAEDIWN--------------------------------------- 317

PFIT_1407400 NNLLKALSIHNKINVAEDIWN--------------------------------------- 317

PFTANZ_05018 NNLLKALSIHNKINVAEDIWN--------------------------------------- 316

PCHAS_103660 NNLLKSLSLSNKINIIENIWN--------------------------------------- 307

PY17X_1038200 NNILKSLSLSNKINIIENIWN--------------------------------------- 315

PYO3222 NNILKSLSLSNKINIIENIWN--------------------------------------- 315

PYYM_1037900 NNILKSLSLSNKINIIENIWN--------------------------------------- 315

PBANKA_103580 NNILKSLSLSNKINIIENIWN--------------------------------------- 312

* *: . : .

AthPPR10 SGAVFPSWVILRTLVIANFKCRRLDGMETAFQEVKARGYNPDLVIFNSMLSIYAKNGMYS 518

Toxoplasma EDVEFPEKQRL------------------------------------------------R 620

PVX_086110 N----------------------------------------------------------- 318

PKH_134050 N----------------------------------------------------------- 348

C922_03803 N----------------------------------------------------------- 322

PCYB_135000 N----------------------------------------------------------- 339

PRCDC_1405700 N----------------------------------------------------------- 315

PF3D7_1406400 N----------------------------------------------------------- 316

PFFVO_05018 N----------------------------------------------------------- 318

PFIT_1407400 N----------------------------------------------------------- 318

PFTANZ_05018 N----------------------------------------------------------- 317

PCHAS_103660 N----------------------------------------------------------- 308

PY17X_1038200 N----------------------------------------------------------- 316

PYO3222 N----------------------------------------------------------- 316

PYYM_1037900 N----------------------------------------------------------- 316

PBANKA_103580 N----------------------------------------------------------- 313

.

AthPPR10 KATEVFDSIKRSGLSPDLITYNSLMDMYAKCSESWEAEKILNQLKCSQTMK----PDVVS 574

Toxoplasma AGEALDQDLAPSKAAPTLRTFELLLRACSQQRQAFALAQIWRDFEELQAT-VAETQNASP 679

PVX_086110 -------EFDELMLTPNEISYGYMLKVYSIVDD---YEKAFKLFKEMQMKKMLSNKNIIP 368

PKH_134050 -------EFDELMLTPNAISYAYMLNAYSIVDD---YEKAFKLFKEMQMKKMLNNKNIIP 398

C922_03803 -------EFDDLMLTPNAISYAYMLNVYSIMDD---YEKAFKLFKEMQMKKLLNNKNIIP 372

PCYB_135000 -------EFDDLMLTPNAISYGFMLNAYSIVDD---YEKAFKLFKEMHMKKLLNKKNIIP 389

PRCDC_1405700 -------EFDELLLTPNNLSYQILLKIYSHIDN---YEKAFKLFKEMQINKLLNNKNILP 365

PF3D7_1406400 -------EFDELLLTPNNLSYQILLKIYSHIDN---YEKAFKLFKEMQVNKLLNNKNILP 366

PFFVO_05018 -------EFDELLLTPNNLSYQILLKIYSHIDN---YEKAFKLFKEMQVNKLLNNKNILP 368

PFIT_1407400 -------EFDELLLTPNNLSYQILLKIYSHIDN---YEKAFKLFKEMQVNKLLNNKNILP 368

PFTANZ_05018 -------EFDELLLTPNNLSYQILLKIYSHIDN---YEKAFKLFKEMQVNKLLNNKNILP 367

PCHAS_103660 -------EYDELLLTHNSVSYQIMLNVYGNIDD---YEKAFKLFKEMQIKKMLNKKNILP 358

PY17X_1038200 -------EYDELLLVQNSVSYQIMLNVYSNIDD---YEKAFKLFKEMQMKKMLNKKNILP 366

PYO3222 -------EYDELLLVQNSVSYQIMLNVYSNIDD---YEKAFKLFKEMQMKKMLNKKNILP 366

PYYM_1037900 -------EYDELLLVQNSVSYQIMLNVYSNIDD---YEKAFKLFKEMQMKKMLNKKNILP 366

PBANKA_103580 -------EYDELLLTQNSVSYQIMLNVYSNIDD---YEKSFKLFKEMQMKKMLNKKNILP 363

. :: :: . : : . :: : :

AthPPR10 Y---------NTVINGFCKQGLVKEAQRVLS------------------EMVADGMAPCA 607

Toxoplasma FRLPSAAPCVASAIQGFAACGLWTYALRLLLLLHKETGDARQRHCELLSDLLRDAREAAE 739

PVX_086110 F---------VYTINAFKNCGIYNYAIYVLRVAKLLGV-SSED----LLKLYNDAMVACV 414

PKH_134050 F---------VYTIHAFKNCGIYNYAIYVLRVAKLLNV-SSED----LLKLYNDAMVACI 444

C922_03803 F---------VYTINAFKNCGIYNYAIYVLRVAKLLNV-SSED----LLKLYNDAMIACI 418

PCYB_135000 F---------VYTINAFKNCGIYNYAIYVLRVAKLLDV-FSED----LLKLYNNAMIACV 435

PRCDC_1405700 F---------IYTIESTKNCGIYNYAIYVLRVAKLLNF-KAND----LLMLYNNTMISCI 411

PF3D7_1406400 F---------IYTIESTKNCGIYNYAIYVLRVAKLLNF-KAND----LLMLYNNTMISCI 412

PFFVO_05018 F---------IYTIESTKNCGIYNYAIYVLRVAKLLNF-KAND----LLMLYNNTMISCI 414

PFIT_1407400 F---------IYTIESTKNCGIYNYAIYVLRVAKLLNF-KAND----LLMLYNNTMISCI 414

PFTANZ_05018 F---------IYTIESTKNCGIYNYAIYVLRVAKLLNF-KAND----LLMLYNNTMISCI 413

PCHAS_103660 F---------VYVLNSFKNCGIYSYSIYVLRIAKLIGI-VGKD----LLFLYNNAMITCV 404

PY17X_1038200 F---------VYTINSFKNCGIYNYSIYVLRIAKLIGI-VGKD----LLFLYNNAMISCI 412

PYO3222 F---------VYTINSFKNCGIYNYSIYVLRIAKLIGI-VGKD----LLFLYNNAMISCI 412

PYYM_1037900 F---------VYTINSFKNCGIYNYSIYVLRIAKLIGI-VGKD----LLFLYNNAMISCI 412

PBANKA_103580 F---------VYTINSFKNCGIYNYSIYVLRIAKLIGI-VGKN----ILFLYNNAMIACI 409

: .:.. *: . : :* : : .

AthPPR10 V----------------------------------------------------TYHTLVG 615

Toxoplasma RSGAARETQSKMRSENEGERSSEQKDGDARAAASESGDWERCLEGRFVDLGVEPYLAVLR 799

PVX_086110 NAK---KYDVVI--SLYAELITMQEKGAPS-----------------LEISISTLGFVLL 452

PKH_134050 NSK---KYEVVI--SLYAELVTMQEKGAPS-----------------LEINISTLGFVLL 482

C922_03803 SCK---KYDVVI--SLYAELITMQEKGAPS-----------------LEINISTLGFVLL 456

PCYB_135000 NCK---KYDVVI--SLYAELITMQEKGAPS-----------------LEINISTLGFVLL 473

PRCDC_1405700 NSK---KYDVII--SLYAELINMQQK-DTS-----------------FQININTLTFVLL 448

PF3D7_1406400 NSK---KYDVII--SLYAELINMQQK-DTS-----------------FQININTLTFVLL 449

PFFVO_05018 NSK---KYDVII--SLYAELINMQQK-DTS-----------------FQININTLTFVLL 451

PFIT_1407400 NSK---KYDVII--SLYAELINMQQK-DTS-----------------FQININTLTFVLL 451

PFTANZ_05018 NSK---KYDVII--SLYAELINMQQK-DTS-----------------FQININTLTFVLL 450

PCHAS_103660 NAK---KYDVII--SLYTELIALQEK-DTS-----------------LTININTLSFVLL 441

PY17X_1038200 NAK---KYDVII--SLYTELIALQEK-DTS-----------------LTININTLSFVLL 449

PYO3222 NAK---KYDVII--SLYTELIALQEK-DTS-----------------LTININTLSFVLL 449

PYYM_1037900 NAK---KYDVII--SLYTELIALQEK-DTS-----------------LTININTLSFVLL 449

PBANKA_103580 NAK---KYDVII--SLYTELIALQEK-DTS-----------------LIININTLSFVLL 446

::

AthPPR10 GYSSLEMFSEAREVIGYMVQHGLKPMELTYRRVV--------------------ESYCRA 655

Toxoplasma ACRDVGAWKPALGILRLLQQRHTQAKLLHALGAARERREAERREQFQQRVRALRRRLSEA 859

PVX_086110 AFRELDMREDFTNLKNLIIQKNYKLTPL-------------------------------- 480

PKH_134050 AFKELNMRDDFINLKNLIIQKNYKLTPL-------------------------------- 510

C922_03803 AFKELNMREDFTNLKNLIIQKNYKLTPL-------------------------------- 484

PCYB_135000 AFKELNMREDFTNLKNLIIQKNYKLTPL-------------------------------- 501

PRCDC_1405700 AFKELNMKQDFINLKNIIIQRNYKLPPL-------------------------------- 476

PF3D7_1406400 AFKELNMKQDFINLKNIIIQRNYKLPPL-------------------------------- 477

PFFVO_05018 AFKELNMKQDFINLKNIIIQRNYKLPPL-------------------------------- 479

PFIT_1407400 AFKELNMKQDFINLKNIIIQRNYKLPPL-------------------------------- 479

PFTANZ_05018 AFKELNMKQDFINLKNIIIQRNYKLPPL-------------------------------- 478

PCHAS_103660 AFKELKMKEDFSNLKNLILQKNYKLTPL-------------------------------- 469

PY17X_1038200 AFKELKMKEDFLNLKNIILQKNYKLTPL-------------------------------- 477

PYO3222 AFKELKMKEDFLNLKNIILQKNYKLTPL-------------------------------- 477

PYYM_1037900 AFKELKMKEDFLNLKNIILQKNYKLTPL-------------------------------- 477

PBANKA_103580 AFKELKMKEDFSNLKNLILQKNYKLTPL-------------------------------- 474

. .: . : : *: : *

AthPPR10 KRFEEARGFLSEVSETDLD-----------FDKKALEAYIE----DAQFGR--------- 691

Toxoplasma KRKTNGADCVGEAAEQQRPTSASPTGDSDPLLSRDMERFLGSLHNSVAVGRTPLLPEFPY 919

PVX_086110 --------CGQLVSEQPH------------------------------------------ 490

PKH_134050 --------CGKLVNEQQND----------------------------------------- 521

C922_03803 --------CGKLVNEEPEW----------------------------------------- 495

PCYB_135000 --------CAKLVSEESDS----------------------------------------- 512

PRCDC_1405700 --------CSKIFSETENY*---------------------------------------- 487

PF3D7_1406400 --------CSKIFSETENY----------------------------------------- 488

PFFVO_05018 --------CSKIFSETENY----------------------------------------- 490

PFIT_1407400 --------CSKIFSETENY*---------------------------------------- 490

PFTANZ_05018 --------CSKIFSETENY----------------------------------------- 489

PCHAS_103660 --------CSKIINEPEDQ*---------------------------------------- 480

PY17X_1038200 --------CSKVINEQES*----------------------------------------- 487

PYO3222 --------CSKVINEQES------------------------------------------ 487

PYYM_1037900 --------CSKVINEQES------------------------------------------ 487

PBANKA_103580 --------CSKVINEP-------------------------------------------- 482

*

AthPPR10 ------------------------------------------------------------ 691

Toxoplasma EAYALTLGTMAAARAWDRVLAVSSEFFSRRDSGACGSASLGADGPAGEANEERKPARRAV 979

PVX_086110 ------------------------------------------------------------ 490

PKH_134050 ------------------------------------------------------------ 521

C922_03803 ------------------------------------------------------------ 495

PCYB_135000 ------------------------------------------------------------ 512

PRCDC_1405700 ------------------------------------------------------------ 487

PF3D7_1406400 ------------------------------------------------------------ 488

PFFVO_05018 ------------------------------------------------------------ 490

PFIT_1407400 ------------------------------------------------------------ 490

PFTANZ_05018 ------------------------------------------------------------ 489

PCHAS_103660 ------------------------------------------------------------ 480

PY17X_1038200 ------------------------------------------------------------ 487

PYO3222 ------------------------------------------------------------ 487

PYYM_1037900 ------------------------------------------------------------ 487

PBANKA_103580 ------------------------------------------------------------ 482

AthPPR10 ------------------------------------------------------------ 691

Toxoplasma GFGGERDEGGQPERGAEGERRDAPLEIRKSVHAYRLMALMHLGRHEEVEAERRSLIRLTE 1039

PVX_086110 ------------------------------------------------------------ 490

PKH_134050 ------------------------------------------------------------ 521

C922_03803 ------------------------------------------------------------ 495

PCYB_135000 ------------------------------------------------------------ 512

PRCDC_1405700 ------------------------------------------------------------ 487

PF3D7_1406400 ------------------------------------------------------------ 488

PFFVO_05018 ------------------------------------------------------------ 490

PFIT_1407400 ------------------------------------------------------------ 490

PFTANZ_05018 ------------------------------------------------------------ 489

PCHAS_103660 ------------------------------------------------------------ 480

PY17X_1038200 ------------------------------------------------------------ 487

PYO3222 ------------------------------------------------------------ 487

PYYM_1037900 ------------------------------------------------------------ 487

PBANKA_103580 ------------------------------------------------------------ 482

AthPPR10 ------------------------------------------------------------ 691

Toxoplasma MERRTRERRDARQREREGGEEKREEGEGSEEAFLPKWREETEHEEGEGGEDEGSHGIATL 1099

PVX_086110 ------------------------------------------------------------ 490

PKH_134050 ------------------------------------------------------------ 521

C922_03803 ------------------------------------------------------------ 495

PCYB_135000 ------------------------------------------------------------ 512

PRCDC_1405700 ------------------------------------------------------------ 487

PF3D7_1406400 ------------------------------------------------------------ 488

PFFVO_05018 ------------------------------------------------------------ 490

PFIT_1407400 ------------------------------------------------------------ 490

PFTANZ_05018 ------------------------------------------------------------ 489

PCHAS_103660 ------------------------------------------------------------ 480

PY17X_1038200 ------------------------------------------------------------ 487

PYO3222 ------------------------------------------------------------ 487

PYYM_1037900 ------------------------------------------------------------ 487

PBANKA_103580 ------------------------------------------------------------ 482

AthPPR10 ------------------------------------------------------------ 691

Toxoplasma VLEEAETWLMRGVTRERRTQGKDNIYPTPSIHETTPHTYEATHHSFPSPSKSSSASSSAS 1159

PVX_086110 ------------------------------------------------------------ 490

PKH_134050 ------------------------------------------------------------ 521

C922_03803 ------------------------------------------------------------ 495

PCYB_135000 ------------------------------------------------------------ 512

PRCDC_1405700 ------------------------------------------------------------ 487

PF3D7_1406400 ------------------------------------------------------------ 488

PFFVO_05018 ------------------------------------------------------------ 490

PFIT_1407400 ------------------------------------------------------------ 490

PFTANZ_05018 ------------------------------------------------------------ 489

PCHAS_103660 ------------------------------------------------------------ 480

PY17X_1038200 ------------------------------------------------------------ 487

PYO3222 ------------------------------------------------------------ 487

PYYM_1037900 ------------------------------------------------------------ 487

PBANKA_103580 ------------------------------------------------------------ 482

AthPPR10 ------------------------------------------------------------ 691

Toxoplasma SSASSSASSSASSSSFSSASSSSFSSSPLQPSFIAGSGGGRSMSLRDVRHLEGVSAMSEG 1219

PVX_086110 ------------------------------------------------------------ 490

PKH_134050 ------------------------------------------------------------ 521

C922_03803 ------------------------------------------------------------ 495

PCYB_135000 ------------------------------------------------------------ 512

PRCDC_1405700 ------------------------------------------------------------ 487

PF3D7_1406400 ------------------------------------------------------------ 488

PFFVO_05018 ------------------------------------------------------------ 490

PFIT_1407400 ------------------------------------------------------------ 490

PFTANZ_05018 ------------------------------------------------------------ 489

PCHAS_103660 ------------------------------------------------------------ 480

PY17X_1038200 ------------------------------------------------------------ 487

PYO3222 ------------------------------------------------------------ 487

PYYM_1037900 ------------------------------------------------------------ 487

PBANKA_103580 ------------------------------------------------------------ 482

AthPPR10 ------------------------------------------------------------ 691

Toxoplasma LKEGRGEETSVARRAEFLIGGGTKRKVCVQEEDRGDAVAEGEANHKSVCRDETGEEHSGR 1279

PVX_086110 ------------------------------------------------------------ 490

PKH_134050 ------------------------------------------------------------ 521

C922_03803 ------------------------------------------------------------ 495

PCYB_135000 ------------------------------------------------------------ 512

PRCDC_1405700 ------------------------------------------------------------ 487

PF3D7_1406400 ------------------------------------------------------------ 488

PFFVO_05018 ------------------------------------------------------------ 490

PFIT_1407400 ------------------------------------------------------------ 490

PFTANZ_05018 ------------------------------------------------------------ 489

PCHAS_103660 ------------------------------------------------------------ 480

PY17X_1038200 ------------------------------------------------------------ 487

PYO3222 ------------------------------------------------------------ 487

PYYM_1037900 ------------------------------------------------------------ 487

PBANKA_103580 ------------------------------------------------------------ 482

AthPPR10 ------------------------------------------------------------ 691

Toxoplasma SLDRFFAGLEPVSEREARAVAEAVERRRTEEHWRQEKMQSGVKRATGDREGMQPKRSADP 1339

PVX_086110 ------------------------------------------------------------ 490

PKH_134050 ------------------------------------------------------------ 521

C922_03803 ------------------------------------------------------------ 495

PCYB_135000 ------------------------------------------------------------ 512

PRCDC_1405700 ------------------------------------------------------------ 487

PF3D7_1406400 ------------------------------------------------------------ 488

PFFVO_05018 ------------------------------------------------------------ 490

PFIT_1407400 ------------------------------------------------------------ 490

PFTANZ_05018 ------------------------------------------------------------ 489

PCHAS_103660 ------------------------------------------------------------ 480

PY17X_1038200 ------------------------------------------------------------ 487

PYO3222 ------------------------------------------------------------ 487

PYYM_1037900 ------------------------------------------------------------ 487

PBANKA_103580 ------------------------------------------------------------ 482

AthPPR10 ------------------------------------------------------------ 691

Toxoplasma EGDKNDPGCQTAGNDPGGETGLRRREGERDGVNEEAGGREKGASGQIEREREGEHDTEEA 1399

PVX_086110 ------------------------------------------------------------ 490

PKH_134050 ------------------------------------------------------------ 521

C922_03803 ------------------------------------------------------------ 495

PCYB_135000 ------------------------------------------------------------ 512

PRCDC_1405700 ------------------------------------------------------------ 487

PF3D7_1406400 ------------------------------------------------------------ 488

PFFVO_05018 ------------------------------------------------------------ 490

PFIT_1407400 ------------------------------------------------------------ 490

PFTANZ_05018 ------------------------------------------------------------ 489

PCHAS_103660 ------------------------------------------------------------ 480

PY17X_1038200 ------------------------------------------------------------ 487

PYO3222 ------------------------------------------------------------ 487

PYYM_1037900 ------------------------------------------------------------ 487

PBANKA_103580 ------------------------------------------------------------ 482

AthPPR10 ------------------------------------------------------------ 691

Toxoplasma RERLEEKEEEREKENSEREGRAIRAVSGERGLSVSAFLSSVLGPRRAVLESMREEPVLED 1459

PVX_086110 ------------------------------------------------------------ 490

PKH_134050 ------------------------------------------------------------ 521

C922_03803 ------------------------------------------------------------ 495

PCYB_135000 ------------------------------------------------------------ 512

PRCDC_1405700 ------------------------------------------------------------ 487

PF3D7_1406400 ------------------------------------------------------------ 488

PFFVO_05018 ------------------------------------------------------------ 490

PFIT_1407400 ------------------------------------------------------------ 490

PFTANZ_05018 ------------------------------------------------------------ 489

PCHAS_103660 ------------------------------------------------------------ 480

PY17X_1038200 ------------------------------------------------------------ 487

PYO3222 ------------------------------------------------------------ 487

PYYM_1037900 ------------------------------------------------------------ 487

PBANKA_103580 ------------------------------------------------------------ 482

AthPPR10 ------------------------------------------------------------ 691

Toxoplasma TVTEEPESLELHADAEAQLEAKQREGESRDRSGENTEASTERRNSPDREEEKQDPVTTAT 1519

PVX_086110 ------------------------------------------------------------ 490

PKH_134050 ------------------------------------------------------------ 521

C922_03803 ------------------------------------------------------------ 495

PCYB_135000 ------------------------------------------------------------ 512

PRCDC_1405700 ------------------------------------------------------------ 487

PF3D7_1406400 ------------------------------------------------------------ 488

PFFVO_05018 ------------------------------------------------------------ 490

PFIT_1407400 ------------------------------------------------------------ 490

PFTANZ_05018 ------------------------------------------------------------ 489

PCHAS_103660 ------------------------------------------------------------ 480

PY17X_1038200 ------------------------------------------------------------ 487

PYO3222 ------------------------------------------------------------ 487

PYYM_1037900 ------------------------------------------------------------ 487

PBANKA_103580 ------------------------------------------------------------ 482

AthPPR10 ------------------------------------------------------------ 691

Toxoplasma QETDANTGGTLSQAGGRKSEAASRSESVETSLDLLLRLKHKTPAETQQLEDGPPRWLARR 1579

PVX_086110 ------------------------------------------------------------ 490

PKH_134050 ------------------------------------------------------------ 521

C922_03803 ------------------------------------------------------------ 495

PCYB_135000 ------------------------------------------------------------ 512

PRCDC_1405700 ------------------------------------------------------------ 487

PF3D7_1406400 ------------------------------------------------------------ 488

PFFVO_05018 ------------------------------------------------------------ 490

PFIT_1407400 ------------------------------------------------------------ 490

PFTANZ_05018 ------------------------------------------------------------ 489

PCHAS_103660 ------------------------------------------------------------ 480

PY17X_1038200 ------------------------------------------------------------ 487

PYO3222 ------------------------------------------------------------ 487

PYYM_1037900 ------------------------------------------------------------ 487

PBANKA_103580 ------------------------------------------------------------ 482

AthPPR10 ------------------------------------------------------------ 691

Toxoplasma SGTGEPGAEAVAGSRRGVHPPDGRAGVGIPGGGAGSQRQRQETRGQSAFLFAGDHGWGWP 1639

PVX_086110 ------------------------------------------------------------ 490

PKH_134050 ------------------------------------------------------------ 521

C922_03803 ------------------------------------------------------------ 495

PCYB_135000 ------------------------------------------------------------ 512

PRCDC_1405700 ------------------------------------------------------------ 487

PF3D7_1406400 ------------------------------------------------------------ 488

PFFVO_05018 ------------------------------------------------------------ 490

PFIT_1407400 ------------------------------------------------------------ 490

PFTANZ_05018 ------------------------------------------------------------ 489

PCHAS_103660 ------------------------------------------------------------ 480

PY17X_1038200 ------------------------------------------------------------ 487

PYO3222 ------------------------------------------------------------ 487

PYYM_1037900 ------------------------------------------------------------ 487

PBANKA_103580 ------------------------------------------------------------ 482

AthPPR10 --------------------------------------- 691

Toxoplasma PHWEEKLDKARARLQRTNDESGETEENAKTETPWGMRPT 1678

PVX_086110 --------------------------------------- 490

PKH_134050 --------------------------------------- 521

C922_03803 --------------------------------------- 495

PCYB_135000 --------------------------------------- 512

PRCDC_1405700 --------------------------------------- 487

PF3D7_1406400 --------------------------------------- 488

PFFVO_05018 --------------------------------------- 490

PFIT_1407400 --------------------------------------- 490

PFTANZ_05018 --------------------------------------- 489

PCHAS_103660 --------------------------------------- 480

PY17X_1038200 --------------------------------------- 487

PYO3222 --------------------------------------- 487

PYYM_1037900 --------------------------------------- 487

PBANKA_103580 --------------------------------------- 482

**Supplemental Figure S3. Gel filtration chromatography of His_6_-TRX-PfPPR1 and corresponding SDS-PAGE gel**. Blue line represents absorbance at 280 nm, red line absorbance at 260 nm and brown line conductivity. The trace for absorbance at 280 nm (blue line) shows four major peaks eluted from the S200 10/300 column. Based on calibration of the S200 10/300 column, the second peak corresponds to a molecular weight of approximately 135 kDa, and the estimated molecular weight of the His_6_-TRX-*Pf*PPR1 dimer is 141.6 kDa. Spontaneous cleavage of the TRX-Histag is observed in the second peak, corresponding to PPR protein without the His_6_-TRX tag (estimated molecular weight of 56.8 kDa) with a smaller amount in the third peak. The fourth peak corresponds to the His_6_-TRX tag only (14 kDa).

**Supplemental Figure S4. Western blot of purified *Pf*PPR1 protein and *P. falciparum* 3D7 lysate using the purified polyclonal anti-*Pf*PPR1 antibody.** Purified PfPPR1 protein was run on 10% SDS-PAGE gel along with *P. falciparum 3D7* cell lysate. After incubation with the purified *Pf*PPR1 polyclonal rabbit antibody and a secondary goat anti-rabbit antibody conjugated to HRP, no *Pf*PPR1 protein could be detected in the *P. falciparum* 3D7 lysate (Panel A).. The positive control (recombinant *Pf*PPR1) shows a band of the correct size (*Pf*PPR1 + TRX His_6_ ~ 72.8 kDa) .The ponceau stained gel (Panel B) shows good transfer of proteins of all molecular weights. Size markers are shown in kDa.

**Supplemental Figure S5. Circular Dichroism (CD) spectrum of purified *Pf*PPR1.** *Pf*PPR1 minus His-TRX tag at 25°C in 10 mM potassium phosphate pH 8.0, 50 mM Na fluoride. Spectrum is typical of that for an alpha-helical protein.

**Supplemental Figure S6. Analytical ultracentrifugation (AUC) sedimentation velocity data for *Pf*PPR1 minus His_6_-TRX tag**. The residuals are from the fit with the continuous c(s) distribution model. Component sedimentation coefficient distribution for PPR at 1.8 mg/mL showing populations of dimeric (fitted mass of 109 kDa) and higher-order species, fitting to a uniform frictional ratio of Fk,w = 1.378. The r.m.s.d. was 0.016.

**Supplementary Figure S7**. **SWISS-MODEL** analysis of PPR1 from both *Toxoplasma* and *Plasmodium.*

**Supplemental Figure S8. *Pf*PPR1 – RNA pull down assays.** (A) Five 150 nt RNA molecules (RNAs 1- 5, sequences as shown in Figure 3) and apicoplast RNA transcripts (*LSU rRNA – rpoB* and *tufA – clpC*) were used in a pull-down experiment. Biotinylated RNA was bound to streptavidin beads and used as ‘bait’ to pull down *Pf*PPR1 protein (with the TRX-His_6_ tag removed). Bound *Pf*PPR1 was protein detected using a purified polyclonal anti-*Pf*PPR1 antibody from rabbit. Loading controls (PPR1 only) and a no RNA control reaction showed no non-specific binding in the absence of RNA. (B) The same pull down experiment using RNA oligonucleotides 1 – 3. The same result was obtained when the experiment was repeated (E). Details of RNA sequences are given in the legend to Figure 3, and specific sequences are shown (C and D).

**Supplemental Figure S9. *Pf*PPR1 shows specific binding to apicoplast RNA transcripts.** *Pf*PPR1 binding to apicoplast RNA transcripts was tested in a gel shift experiment. Recombinant *Pf*PPR1 (minus TRX-His_6_) causes a shift in the migration of *in vitro* transcribed apicoplast RNA molecules following incubation for one hour (Panel A). No shift is seen in the RNA if it is not bound to PPR1. Panel B shows that no shift is seen when PPR1 is incubated with *in vitro* transcribed RNA from a nuclear encoded *P. falciparum* gene (PF11_0264, or from an *E. coli*  codon-optomized *P. falciparum* gene (PF14_0061) and a *P. falciparum 3D7* nuclear gene (PF11_0264).

**Supplemental Figure S10 Gel filtration shows a change in elution profile when *Pf*PPR1 is bound to RNA.** This is a repeat of the data shown in Figure 4, showing that the change in mobility following gel filtration is reproducible.

**Supplemental Figure S11. Ribonuclease A protection assays.** RNA transcripts 1, 4 and 5 and RNA oligos 1,2 and 3 were incubated in a 1:1 molar ratio with *Pf*PPR1 prior to treatment with RNase A. Samples were analyzed using a native acrylamide gel (ladder in nt). Experiments with no *Pf*PPR1 bound to RNA showed complete degradation by RNase A

**Plasmid sequences used for *T. gondii* transformation**.

**pCRISPR/Cas9-GFP_PPR-sgRNA**

*392-1148 SAG1 Promoter*

*1149-5022 Cas9-GFP coding sequence*

*6178-6490 SAG1 Terminator*

*6508-7053 U6 Promoter*

*7054-7170 sgRNA (7060-7079 PPR protospacer)*

*7420-8087 Col E1 origin*

*8235-9095 AMP*

1 ctaaattgta agcgttaata ttttgttaaa attcgcgtta aatttttgtt aaatcagctc

61 attttttaac caataggccg aaatcggcaa aatcccttat aaatcaaaag aatagaccga

121 gatagggttg agtggccgct acagggcgct cccattcgcc attcaggctg cgcaactgtt

181 gggaagggcg tttcggtgcg ggcctcttcg ctattacgcc agctggcgaa agggggatgt

241 gctgcaaggc gattaagttg ggtaacgcca gggttttccc agtcacgacg ttgtaaaacg

301 acggccagtg agcgcgacgt aatacgactc actatagggc gaattggcgg aaggccgtca

361 aggcctaggc gcgccaggtc tcatgccgga gaagctttta catccgttgc cttttccacg

421 gtccgtgatt tcatgtgcgt gcagcttcaa agactggtcg ttgcgactaa taagactgca

481 gtgacaggtc gaatggtggg caccttgctg atgactatct actgcaaagt ctgagacaac

541 gaacgaaact tcccacacga ggcatttgaa actgacggtg tctaggtaat atgcactgca

601 agacacggta ctggggcctc gctgaattag gggccgatct cgttgcccta tcagtgctca

661 cagtgccgca acgtaacacc agggcaggtt cttgacagtg gcaacaatgt gcgacgggcg

721 tgtgaacgtt tcgtagtcat agcgctagca cgtacctagc cacatggtcg tgaggagctt

781 taccatgcgt ctagaaggtg gatgcgggac acgccttcct ggcctttggc tcccgagacg

841 cgtgttctaa ccacaaacct tgagacgcgt gttccaacca cgcaccctga cacgcgtgtt

901 ccaaccacgc accctgagac gcgtgttcta accacgcacc ctgagacgcg tgttctaacc

961 acgcaccctg agacgcgtgt tctgccgcac aatgtgcacc tgtaggaagc tgtagtcact

1021 gctgattctc actgttctcg gcaagggccg acgaccggag tacagttttt gtgggcagag

1081 ccgttgtgca gctttccgtt cttctcggtt gtgtcacatg tgtcattgtc gtgtaaacac

1141 acggttgtat gtcggtttcg ctgcaccact tcattatttc ttctggtttt ttgacgagta

1201 tgcatctaga caaaatggac aagaagtaca gcatcggcct ggacatcggc accaactctg

1261 tgggctgggc cgtgatcacc gacgagtaca aggtgcccag caagaaattc aaggtgctgg

1321 gcaacaccga ccggcacagc atcaagaaga acctgatcgg cgccctgctg ttcgacagcg

1381 gagaaacagc cgaggccacc cggctgaaga gaaccgccag aagaagatac accagacgga

1441 agaaccggat ctgctatctg caagagatct tcagcaacga gatggccaag gtggacgaca

1501 gcttcttcca cagactggaa gagtccttcc tggtggaaga ggataagaag cacgagcggc

1561 accccatctt cggcaacatc gtggacgagg tggcctacca cgagaagtac cccaccatct

1621 accacctgag aaagaaactg gtggacagca ccgacaaggc cgacctgcgg ctgatctatc

1681 tggccctggc ccacatgatc aagttccggg gccacttcct gatcgagggc gacctgaacc

1741 ccgacaacag cgacgtggac aagctgttca tccagctggt gcagacctac aaccagctgt

1801 tcgaggaaaa ccccatcaac gccagcggcg tggacgccaa ggccatcctg tctgccagac

1861 tgagcaagag cagacggctg gaaaatctga tcgcccagct gcccggcgag aagaagaatg

1921 gcctgttcgg caacctgatt gccctgagcc tgggcctgac ccccaacttc aagagcaact

1981 tcgacctggc cgaggatgcc aaactgcagc tgagcaagga cacctacgac gacgacctgg

2041 acaacctgct ggcccagatc ggcgaccagt acgccgacct gtttctggcc gccaagaacc

2101 tgtccgacgc catcctgctg agcgacatcc tgagagtgaa caccgagatc accaaggccc

2161 ccctgagcgc ctctatgatc aagagatacg acgagcacca ccaggacctg accctgctga

2221 aagctctcgt gcggcagcag ctgcctgaga agtacaaaga gattttcttc gaccagagca

2281 agaacggcta cgccggctac atcgatggcg gagccagcca ggaagagttc tacaagttca

2341 tcaagcccat cctggaaaag atggacggca ccgaggaact gctcgtgaag ctgaacagag

2401 aggacctgct gcggaagcag cggaccttcg acaacggcag catcccccac cagatccacc

2461 tgggagagct gcacgccatt ctgcggcggc aggaagattt ttacccattc ctgaaggaca

2521 accgggaaaa gatcgagaag atcctgacct tccgcatccc ctactacgtg ggccctctgg

2581 ccaggggaaa cagcagattc gcctggatga ccagaaagag cgaggaaacc atcaccccct

2641 ggaacttcga ggaagtggtg gacaagggcg ccagcgccca gagcttcatc gagcggatga

2701 ccaacttcga taagaacctg cccaacgaga aggtgctgcc caagcacagc ctgctgtacg

2761 agtacttcac cgtgtacaac gagctgacca aagtgaaata cgtgaccgag ggaatgagaa

2821 agcccgcctt cctgagcggc gagcagaaaa aagccatcgt ggacctgctg ttcaagacca

2881 accggaaagt gaccgtgaag cagctgaaag aggactactt caagaaaatc gagtgcttcg

2941 actccgtgga aatctccggc gtggaagatc ggttcaacgc ctccctgggc acataccacg

3001 atctgctgaa aattatcaag gacaaggact tcctggacaa tgaggaaaac gaggacattc

3061 tggaagatat cgtgctgacc ctgacactgt ttgaggacag agagatgatc gaggaacggc

3121 tgaaaaccta tgcccacctg ttcgacgaca aagtgatgaa gcagctgaag cggcggagat

3181 acaccggctg gggcaggctg agccggaagc tgatcaacgg catccgggac aagcagtccg

3241 gcaagacaat cctggatttc ctgaagtccg acggcttcgc caacagaaac ttcatgcagc

3301 tgatccacga cgacagcctg acctttaaag aggacatcca gaaagcccag gtgtccggcc

3361 agggcgatag cctgcacgag cacattgcca atctggccgg cagccccgcc attaagaagg

3421 gcatcctgca gacagtgaag gtggtggacg agctcgtgaa agtgatgggc cggcacaagc

3481 ccgagaacat cgtgatcgaa atggccagag agaaccagac cacccagaag ggacagaaga

3541 acagccgcga gagaatgaag cggatcgaag agggcatcaa agagctgggc agccagatcc

3601 tgaaagaaca ccccgtggaa aacacccagc tgcagaacga gaagctgtac ctgtactacc

3661 tgcagaatgg gcgggatatg tacgtggacc aggaactgga catcaaccgg ctgtccgact

3721 acgatgtgga ccatatcgtg cctcagagct ttctgaagga cgactccatc gataacaaag

3781 tgctgactcg gagcgacaag aaccggggca agagcgacaa cgtgccctcc gaagaggtcg

3841 tgaagaagat gaagaactac tggcgccagc tgctgaatgc caagctgatt acccagagga

3901 agttcgacaa tctgaccaag gccgagagag gcggcctgag cgaactggat aaggccggct

3961 tcatcaagag acagctggtg gaaacccggc agatcacaaa gcacgtggca cagatcctgg

4021 actcccggat gaacactaag tacgacgaga acgacaaact gatccgggaa gtgaaagtga

4081 tcaccctgaa gtccaagctg gtgtccgatt tccggaagga tttccagttt tacaaagtgc

4141 gcgagatcaa caactaccac cacgcccacg acgcctacct gaacgccgtc gtgggaaccg

4201 ccctgatcaa aaagtaccct aagctggaaa gcgagttcgt gtacggcgac tacaaggtgt

4261 acgacgtgcg gaagatgatc gccaagagcg agcaggaaat cggcaaggct accgccaagt

4321 acttcttcta cagcaacatc atgaactttt tcaagaccga gattaccctg gccaacggcg

4381 agatccggaa gcggcctctg atcgagacaa acggcgaaac aggcgagatc gtgtgggata

4441 agggccggga ctttgccacc gtgcggaaag tgctgtctat gccccaagtg aatatcgtga

4501 aaaagaccga ggtgcagaca ggcggcttca gcaaagagtc tatcctgccc aagaggaaca

4561 gcgacaagct gatcgccaga aagaaggact gggaccctaa gaagtacggc ggcttcgaca

4621 gccccaccgt ggcctattct gtgctggtgg tggccaaagt ggaaaagggc aagtccaaga

4681 aactgaagag tgtgaaagag ctgctgggga tcaccatcat ggaaagaagc agcttcgaga

4741 agaatcccat cgactttctg gaagccaagg gctacaaaga agtgaaaaag gacctgatca

4801 tcaagctgcc taagtactcc ctgttcgagc tggaaaacgg ccggaagaga atgctggcct

4861 ctgccggcga actgcagaag ggaaacgaac tggccctgcc ctccaaatat gtgaacttcc

4921 tgtacctggc cagccactat gagaagctga agggctcccc cgaggataat gagcagaaac

4981 agctgtttgt ggaacagcac aaacactacc tggacgagat catcgagcag atcagcgagt

5041 tctccaagag agtgatcctg gccgacgcta atctggacaa ggtgctgagc gcctacaaca

5101 agcacagaga caagcctatc agagagcagg ccgagaatat catccacctg tttaccctga

5161 ccaatctggg agcccctgcc gccttcaagt actttgacac caccatcgac cggaagaggt

5221 acaccagcac caaagaggtg ctggacgcca ccctgatcca ccagagcatc accggcctgt

5281 acgagacacg gatcgacctg tctcagctgg gaggcgacgc ctatccctat gacgtgcccg

5341 attatgccag cctgggcagc ggctccccca agaaaaaacg caaggtggaa gatcctaaga

5401 aaaagcggaa agtggacggc attggtagtg ggagcaacgg cagcagcgga tccgtgagca

5461 agggcgagga gctgttcacc ggggtggtgc ccatcctggt cgagctggac ggcgacgtaa

5521 acggccacaa gttcagcgtg cgcggcgagg gcgagggcga tgccaccaac ggcaagctga

5581 ccctgaagtt catctgcacc accggcaagc tgcccgtgcc ctggcccacc ctcgtgacca

5641 ccctgaccta cggcgtgcag tgcttcagcc gctaccccga ccacatgaag cagcacgact

5701 tcttcaagtc cgccatgccc gaaggctacg tccaggagcg caccatctcc ttcaaggacg

5761 acggcaccta caagacccgc gccgaggtga agttcgaggg cgacaccctg gtgaaccgca

5821 tcgagctgaa gggcatcgac ttcaaggagg acggcaacat cctggggcac aagctggagt

5881 acaacttcaa cagccacaac gtctatatca cggccgacaa gcagaagaac ggcatcaagg

5941 cgaacttcaa gatccgccac aacgtcgagg acggcagcgt gcagctcgcc gaccactacc

6001 agcagaacac ccccatcggc gacggccccg tgctgctgcc cgacaaccac tacctgagca

6061 cccagtccaa gctgagcaaa gaccccaacg agaagcgcga tcacatggtc ctgctggagt

6121 tcgtgaccgc cgccgggatc actctcggca tggacgagct gtacaagtag ttaattaatc

6181 accgttgtgc tcacttctca aatcgacaaa ggaaacacac ttcgtgcagc atgtgcccca

6241 ttataaagaa actgagttgt tccgctgtgg cttgcaggtg tcacatccac aaaaaccggc

6301 cgactctaaa taggagtgtt tcgcagcaag cagcgaaagt ttatgactgg gtccgaatct

6361 ctgaacggat gtgtggcgga cctggctgat gttgatcgcc gtcgacacac gcgccacatg

6421 ggtcaataca caagacagct atcagttgtt ttagtcgaac cggttaacac aattcttgcc

6481 cccccgaggg cgctgcaagg agtctcagga gcaagtaagc agaagcacgc tgtatttccg

6541 ggagggtgcg atgagacaaa gtgcgcgagt tgaaatcgtc gtggggacga tttcaccgcg

6601 gccacatgtt ggagacactg agggcacacg ggaaacgcga aagatttcaa attaacgtac

6661 ccaaacgcga aagcttgcgc agcatacact cgaagcgaac atcccgaacc atcgagaggc

6721 ggagagcgat aagtctttca cgctgcgaag tgttgcgacg gctgcgccgc tgcactgtga

6781 attgggcgcc aatattgcat cctaggcctg acgcgcctcc tgcagaacgc gagacactgg

6841 gatatgtaga gccaaggggg aaaccttcga actctcgaat gtattctctg acaagaatca

6901 tatttccatc agttctgtca gattttcaaa tggcgacctg cagaggcctg cttcctccct

6961 gtgcgctctt cgaaggggct ttctgtcgcg cagggtcacc tcgtccccga agggggtgtt

7021 tgccttctgg taaatgggga tgtcaagtta gttgagttgt tgagcgagag acagctcagg

7081 ttttagagct agaaatagca agttaaaata aggctagtcc gttatcaact tgaaaaagtg

7141 gcaccgagtc ggtgcttttt tgagctccag cttttgttcc ctttagtgag ggttaattgc

7201 gcgcttggcg tcgctactag aggatgcaca tgtgaccgag ggaattaatt aactggcctc

7261 atgggccttc cgctcactgc ccgctttcca gtcgggaaac ctgtcgtgcc agctgcatta

7321 acatggtcat agctgtttcc ttgcgtattg ggcgctctcc gcttcctcgc tcactgactc

7381 gctgcgctcg gtcgttcggg taaagcctgg ggtgcctaat gagcaaaagg ccagcaaaag

7441 gccaggaacc gtaaaaaggc cgcgttgctg gcgtttttcc ataggctccg cccccctgac

7501 gagcatcaca aaaatcgacg ctcaagtcag aggtggcgaa acccgacagg actataaaga

7561 taccaggcgt ttccccctgg aagctccctc gtgcgctctc ctgttccgac cctgccgctt

7621 accggatacc tgtccgcctt tctcccttcg ggaagcgtgg cgctttctca tagctcacgc

7681 tgtaggtatc tcagttcggt gtaggtcgtt cgctccaagc tgggctgtgt gcacgaaccc

7741 cccgttcagc ccgaccgctg cgccttatcc ggtaactatc gtcttgagtc caacccggta

7801 agacacgact tatcgccact ggcagcagcc actggtaaca ggattagcag agcgaggtat

7861 gtaggcggtg ctacagagtt cttgaagtgg tggcctaact acggctacac tagaagaaca

7921 gtatttggta tctgcgctct gctgaagcca gttaccttcg gaaaaagagt tggtagctct

7981 tgatccggca aacaaaccac cgctggtagc ggtggttttt ttgtttgcaa gcagcagatt

8041 acgcgcagaa aaaaaggatc tcaagaagat cctttgatct tttctacggg gtctgacgct

8101 cagtggaacg aaaactcacg ttaagggatt ttggtcatga gattatcaaa aaggatcttc

8161 acctagatcc ttttaaatta aaaatgaagt tttaaatcaa tctaaagtat atatgagtaa

8221 acttggtctg acagttacca atgcttaatc agtgaggcac ctatctcagc gatctgtcta

8281 tttcgttcat ccatagttgc ctgactcccc gtcgtgtaga taactacgat acgggagggc

8341 ttaccatctg gccccagtgc tgcaatgata ccgcgagaac cacgctcacc ggctccagat

8401 ttatcagcaa taaaccagcc agccggaagg gccgagcgca gaagtggtcc tgcaacttta

8461 tccgcctcca tccagtctat taattgttgc cgggaagcta gagtaagtag ttcgccagtt

8521 aatagtttgc gcaacgttgt tgccattgct acaggcatcg tggtgtcacg ctcgtcgttt

8581 ggtatggctt cattcagctc cggttcccaa cgatcaaggc gagttacatg atcccccatg

8641 ttgtgcaaaa aagcggttag ctccttcggt cctccgatcg ttgtcagaag taagttggcc

8701 gcagtgttat cactcatggt tatggcagca ctgcataatt ctcttactgt catgccatcc

8761 gtaagatgct tttctgtgac tggtgagtac tcaaccaagt cattctgaga atagtgtatg

8821 cggcgaccga gttgctcttg cccggcgtca atacgggata ataccgcgcc acatagcaga

8881 actttaaaag tgctcatcat tggaaaacgt tcttcggggc gaaaactctc aaggatctta

8941 ccgctgttga gatccagttc gatgtaaccc actcgtgcac ccaactgatc ttcagcatct

9001 tttactttca ccagcgtttc tgggtgagca aaaacaggaa ggcaaaatgc cgcaaaaaag

9061 ggaataaggg cgacacggaa atgttgaata ctcatactct tcctttttca atattattga

9121 agcatttatc agggttattg tctcatgagc ggatacatat ttgaatgtat ttagaaaaat

9181 aaacaaatag gggttccgcg cacatttccc cgaaaagtgc cac

**pPPR-mCherry_CAT**

*388-1441 1054 bp of 3’ PPR sequence*

*1442-2152 mCherry coding sequence*

*2157-2469 SAG1 terminator*

*2482-2935 GRA1 promoter*

*2937-3602 CAT (chloramphenicol acetyltransferase) coding sequence*

*3607-3919 SAG1 terminator*

*4128-4795 Col E1 origin*

*4943-5803 AMP*

1 ctaaattgta agcgttaata ttttgttaaa attcgcgtta aatttttgtt aaatcagctc

61 attttttaac caataggccg aaatcggcaa aatcccttat aaatcaaaag aatagaccga

121 gatagggttg agtggccgct acagggcgct cccattcgcc attcaggctg cgcaactgtt

181 gggaagggcg tttcggtgcg ggcctcttcg ctattacgcc agctggcgaa agggggatgt

241 gctgcaaggc gattaagttg ggtaacgcca gggttttccc agtcacgacg ttgtaaaacg

301 acggccagtg agcgcgacgt aatacgactc actatagggc gaattggcgg aaggccgtca

361 aggcctaggc gcgccaggtc tcatgccgga gggaatgcag ccgaagagat cggccgatcc

421 ggagggcgac aagaacgatc ctggttgtca aacagcagga aacgatcccg ggggagagac

481 aggactgaga cgacgagaag gagagagaga cggagtgaat gaagaagcgg gaggtagaga

541 gaaaggcgca tcagggcaga tagaacgaga gcgagaagga gaacacgata cagaagaagc

601 aagagagaga ctcgaagaga aagaagaaga gagagaaaag gagaacagtg aacgggaagg

661 gagagcaata agagcagttt caggagagag aggattgtcg gtttctgctt tcttgagttc

721 cgtcctcggc ccgcgtcgcg cggtgcttga gagcatgcgg gaagagccgg ttttggagga

781 cacggtgacc gaggaacctg agagccttga actgcatgca gacgcagaag cgcagctgga

841 ggcgaagcag agggaaggcg agagccgcga cagaagcggt gaaaacacag aagcctcaac

901 ggagaggaga aactcgccag accgagaaga agagaaacag gatcctgtca cgacggcaac

961 ccaggagaca gatgcgaaca ctgggggaac gctgtcgcag gcgggaggaa gaaagagcga

1021 agcagcttcc agatccgaat ctgtcgagac gagtctggat cttcttcttc gactcaagca

1081 caagacgccg gcagagacgc agcagctcga agatggaccg cctcgctggc tcgccaggcg

1141 aagcggcaca ggggagccgg gagctgaggc cgtcgcaggt agccgccggg gtgtacatcc

1201 acccgacggc cgggcgggag tcggaatccc tggaggaggc gcaggctctc agaggcagag

1261 acaggagacg agagggcaga gtgcatttct ctttgcaggt gaccacggct ggggatggcc

1321 gccgcactgg gaagaaaaac tcgacaaggc tcgagccaga ttgcagcgaa cgaacgacga

1381 aagtggagaa accgaggaaa atgcaaagac agaaacccct tgggggatgc gaccgacttc

1441 gatggtgagc aagggcgagg aggataacat ggccatcatc aaggagttca tgcgcttcaa

1501 ggtgcacatg gagggctccg tgaacggcca cgagttcgag atcgagggcg agggcgaggg

1561 ccgcccctac gagggcaccc agaccgccaa gctgaaggtg accaagggtg gccccctgcc

1621 cttcgcctgg gacatcctgt cccctcagtt catgtacggc tccaaggcct acgtgaagca

1681 ccccgccgac atccccgact acttgaagct gtccttcccc gagggcttca agtgggagcg

1741 cgtgatgaac ttcgaggacg gcggcgtggt gaccgtgacc caggactcct ccctgcagga

1801 cggcgagttc atctacaagg tgaagctgcg cggcaccaac ttcccctccg acggccccgt

1861 aatgcagaag aaaaccatgg gctgggaggc ctcctccgag cggatgtacc ccgaggacgg

1921 cgccctgaag ggcgagatca agcagaggct gaagctgaag gacggcggcc actacgacgc

1981 tgaggtcaag accacctaca aggccaagaa gcccgtgcag ctgcccggcg cctacaacgt

2041 caacatcaag ttggacatca cctcccacaa cgaggactac accatcgtgg aacagtacga

2101 acgcgccgag ggccgccact ccaccggcgg catggacgag ctgtacaagt gagcttatca

2161 ccgttgtgct cacttctcaa atcgacaaag gaaacacact tcgtgcagca tgtgccccat

2221 tataaagaaa ctgagttgtt ccgctgtggc ttgcaggtgt cacatccaca aaaaccggcc

2281 gactctaaat aggagtgttt cgcagcaagc agcgaaagtt tatgactggg tccgaatctc

2341 tgaacggatg tgtggcggac ctggctgatg ttgatcgccg tcgacacacg cgccacatgg

2401 gtcaatacac aagacagcta tcagttgttt tagtcgaacc ggttaacaca attcttgccc

2461 ccccgagggc gctgcaagga gatagcgtgg tactcgtcac gaataccaat cgctggggtc

2521 gcggcgggag gaatatgctg tttgttgacc atacgatcac gctgaacgaa aacatggtat

2581 gagacgccgt aagcgggcac aggttgtttg ccctcgtctc attgcggacc aattcccggt

2641 ccaccgctgc gtctcgactc gacggttgtg accaccccac ttcgcattgg gcagtcggta

2701 aagccacaac attactttgc aattttatcg gttgaaactg ccgagcgagc ttgcgttttt

2761 gggtgctatc ttctcccacc ttttatcagt taagttgtac agtgagtgtc agcttgtttc

2821 gacacgtctg tatagacgca actcggtttg cttgtgttgt ttggtggctg gccaaatcaa

2881 aggctattca tttttcactt gctgttgttc tttgaagaaa tcaagcaaga gtcaaaatgc

2941 tgcatgagaa aaaaatcact ggatatacca ccgttgatat atcccaatcg catcgtaaag

3001 aacattttga ggcatttcag tcagttgctc aatgtaccta taaccagacc gttcagctgg

3061 atattacggc ctttttaaag accgtaaaga aaaataagca caagttttat ccggccttta

3121 ttcacattct tgcccgcctg atgaatgctc atccggaatt ccgtatggca atgaaagacg

3181 gtgagctggt gatatgggat agtgttcacc cttgttacac cgttttccat gagcaaactg

3241 aaacgttttc atcgctctgg agtgaatacc acgacgattt ccggcagttt ctacacatat

3301 attcgcaaga tgtggcgtgt tacggtgaaa acctggccta tttccctaaa gggtttattg

3361 agaatatgtt tttcgtctca gccaatccct gggtgagttt caccagtttt gatttaaacg

3421 tggccaatat ggacaacttc ttcgcccccg ttttcaccat gggcaaatat tatacgcaag

3481 gcgacaaggt gctgatgccg ctggcgattc aggttcatca tgccgtctgt gatggcttcc

3541 atgtcggcag aatgcttaat gaattacaac agtactgcga tgagtggcag ggcggggctt

3601 aagcttatca ccgttgtgct cacttctcaa atcgacaaag gaaacacact tcgtgcagca

3661 tgtgccccat tataaagaaa ctgagttgtt ccgctgtggc ttgcaggtgt cacatccaca

3721 aaaaccggcc gactctaaat aggagtgttt cgcagcaagc agcgaaagtt tatgactggg

3781 tccgaatctc tgaacggatg tgtggcggac ctggctgatg ttgatcgccg tcgacacacg

3841 cgccacatgg gtcaatacac aagacagcta tcagttgttt tagtcgaacc ggttaacaca

3901 attcttgccc ccccgagggc gctactagag gatgcacatg tgaccgaggg aattaattaa

3961 ctggcctcat gggccttccg ctcactgccc gctttccagt cgggaaacct gtcgtgccag

4021 ctgcattaac atggtcatag ctgtttcctt gcgtattggg cgctctccgc ttcctcgctc

4081 actgactcgc tgcgctcggt cgttcgggta aagcctgggg tgcctaatga gcaaaaggcc

4141 agcaaaaggc caggaaccgt aaaaaggccg cgttgctggc gtttttccat aggctccgcc

4201 cccctgacga gcatcacaaa aatcgacgct caagtcagag gtggcgaaac ccgacaggac

4261 tataaagata ccaggcgttt ccccctggaa gctccctcgt gcgctctcct gttccgaccc

4321 tgccgcttac cggatacctg tccgcctttc tcccttcggg aagcgtggcg ctttctcata

4381 gctcacgctg taggtatctc agttcggtgt aggtcgttcg ctccaagctg ggctgtgtgc

4441 acgaaccccc cgttcagccc gaccgctgcg ccttatccgg taactatcgt cttgagtcca

4501 acccggtaag acacgactta tcgccactgg cagcagccac tggtaacagg attagcagag

4561 cgaggtatgt aggcggtgct acagagttct tgaagtggtg gcctaactac ggctacacta

4621 gaagaacagt atttggtatc tgcgctctgc tgaagccagt taccttcgga aaaagagttg

4681 gtagctcttg atccggcaaa caaaccaccg ctggtagcgg tggttttttt gtttgcaagc

4741 agcagattac gcgcagaaaa aaaggatctc aagaagatcc tttgatcttt tctacggggt

4801 ctgacgctca gtggaacgaa aactcacgtt aagggatttt ggtcatgaga ttatcaaaaa

4861 ggatcttcac ctagatcctt ttaaattaaa aatgaagttt taaatcaatc taaagtatat

4921 atgagtaaac ttggtctgac agttaccaat gcttaatcag tgaggcacct atctcagcga

4981 tctgtctatt tcgttcatcc atagttgcct gactccccgt cgtgtagata actacgatac

5041 gggagggctt accatctggc cccagtgctg caatgatacc gcgagaacca cgctcaccgg

5101 ctccagattt atcagcaata aaccagccag ccggaagggc cgagcgcaga agtggtcctg

5161 caactttatc cgcctccatc cagtctatta attgttgccg ggaagctaga gtaagtagtt

5221 cgccagttaa tagtttgcgc aacgttgttg ccattgctac aggcatcgtg gtgtcacgct

5281 cgtcgtttgg tatggcttca ttcagctccg gttcccaacg atcaaggcga gttacatgat

5341 cccccatgtt gtgcaaaaaa gcggttagct ccttcggtcc tccgatcgtt gtcagaagta

5401 agttggccgc agtgttatca ctcatggtta tggcagcact gcataattct cttactgtca

5461 tgccatccgt aagatgcttt tctgtgactg gtgagtactc aaccaagtca ttctgagaat

5521 agtgtatgcg gcgaccgagt tgctcttgcc cggcgtcaat acgggataat accgcgccac

5581 atagcagaac tttaaaagtg ctcatcattg gaaaacgttc ttcggggcga aaactctcaa

5641 ggatcttacc gctgttgaga tccagttcga tgtaacccac tcgtgcaccc aactgatctt

5701 cagcatcttt tactttcacc agcgtttctg ggtgagcaaa aacaggaagg caaaatgccg

5761 caaaaaaggg aataagggcg acacggaaat gttgaatact catactcttc ctttttcaat

5821 attattgaag catttatcag ggttattgtc tcatgagcgg atacatattt gaatgtattt

5881 agaaaaataa acaaataggg gttccgcgca catttccccg aaaagtgcca c

//
