# Supplementary material for: An essential pentatricopeptide repeat protein in the apicomplexan remnant chloroplast
Source: Cell Microbiol. 2019 Sep 16;21(12):e13108. doi: 10.1111/cmi.13108 (PMC6899631; doi:10.1111/cmi.13108)
Supplement: Supplementary file 12 — Data S1: Supporting information [file CMI-21-na-s004.pdf]

1 **SUPPLEMENTAL INFORMATION**

## Supplemental Table S1. Predicted apicoplast localization.

| | | PlasmoAP Prediction | | | | | PlasMit Prediction | |
|---|---|---|---|---|---|---|---|---|
| **Protein ID** | **Species** | **Signal Peptide Value** | **Decision** | **Apicoplast Targeting Peptide Value** | **Decision** | **Targeted to the Apicoplast** | **Jury** | **Strict** |
| PF14_ 0061 | *Plasmodium falciparum 3D7* | 3/4 | + | 5/5 | ++ | YES | 99% non - mito | n/a |
| PFTANZ_0501 8 | *Plasmodium falciparum Tanzania* | 3/4 | + | 5/5 | ++ | YES | 99% non - mito | n/a |
| PFFVO_ 04677 | *Plasmodium falciparum Vietnam Oak-Knoll* | 3/4 | + | 5/5 | ++ | YES | 99% non - mito | n/a |
| PRCDC_14057 00 | *Plasmodium reichenowi* | 2/4 | 0 | 5/5 | ++ | NO? | 99% non - mito | n/a |
| PKH_ 13050 | *Plasmodium knowlesi strain H* | 4/4 | ++ | 5/5 | ++ | YES | 99% non - mito | n/a |
| PVX_ 086110 | *Plasmodium vivax Sal1* | 3/4 | + | 3/5 | - | NO? | mito 91% | failed |
| C922_ 03803 | *Plasmodium inui San Antonio I* | 0/4 | - | 2/5 | - | NO | 99% non-mito | n/a |
| PYO3222 | *Plasmodium yoelii 17XNL* | 3/4 | + | 5/5 | ++ | YES | 99% non-mito | n/a |
| PY17X_ 1038200 | *Plasmodium yoelii 17X* | 3/4 | + | 5/5 | ++ | YES | 99% non-mito | n/a |
| YYE_ 02192 | *Plasmodium vinckei* | 4/4 | ++ | 5/5 | ++ | YES | 99% non-mito | n/a |
| PBANKA_103 580 | *Plasmodium berghei ANKA* | 4/4 | ++ | 5/5 | ++ | YES | 99% non-mito | n/a |
| PCHAS_10366 0 | *Plasmodium chaubadi* | 4/4 | ++ | 5/5 | ++ | YES | 99% non-mito | n/a |
| PCYB_ 135000 | *Plasmodium cynmolgi Strain B* | 0/4 | - | 0/5 | - | NO | 99% non-mito | n/a |
| PFIT_ 1407400 | *Plasmodium falciparum IT* | 3/4 | + | 5/5 | ++ | YES | 99% non-mito | n/a |

**Supplemental Table S2. Conditions trialled for detection of PfPPR1 *in P. falciparum 3D7* lysate by Western Blot**

| **Problem** | **Conditions trialled** |
|---|---|
| Transfer efficiency | iBLOT (dry transfer), wet transfer, temperature of wet transfer. Checked transfer efficiency by staining the membrane with Ponceau and by staining the gel post-transfer with coomassie blue. Wet transfer at room temperature best. |
| Background in western blot images | Tested preimmune and postimmune serum, background also present in preimmune serum |
| Blocking solution to decrease background | 1, 2 and 5% (w/v) BSA and 5 and 10% (w/v) non-fat milk powder tested. 10% skim milk powder lowest background. |
| Increase specificity | Included 0.5 M NaCl in 1xTBS + tween washes. Did not make any difference. |
| Primary antibody detection | Purified primary antibody. Compared different dilutions of purified and non-purified primary antibody (1:200, 1:500, 1:1000, 1:2000). Decided upon 1:1000 dilution |
| Secondary antibody detection | Tested for specificity of anti-rabbit AlexaFluor-568 antibody by performing a western blot on purified PPR and plasmodium lysate with no primary antibody. No non-specific binding observed. |

**Supplemental Table S3. Primers used in this study**

| Primer | Sequence |
|---|---|
| clpC FWD | CCTTTATATGGAGCTCGT |
| clpC FWD + T7 | GCCATAATACGACTCACTATAGCCTTTATATGGAGCTCGT |
| clpC REV | CTGATATTTCTTTTAATTTTATTC |
| *KDPPR-Fwd* | GTCTGACAGTCTGTCTCTATGGTGCGCCCGTTGTCGAGACAACT CTGTC |
| *KDPPR-Rev* | GGAAAGAGAAGAGAGAAGGACGGCGCATGCTAGCAGATCTGG TTGAAGAC |
| LSU rRNA + T7 FWD | GCCATAATACGACTCACTATAG GTGTATAATTCCTAATAAGTTGA |
| LSU rRNA FWD | GTGTATAATTCCTAATAAGTTGA |
| LSUrRNA + T7 FWD | GCCATAATACGACTCACTATAGGTGTATAATTCCTAATAAGTT GA |
| LSUrRNA FWD | GTGTATAATTCCTAATAAGTTGA |
| PF11_0264 minus leader + T7 FWD | GCCATAATACGACTCACTATAGAAGGACACACATATTATTGAT |
| PF11_0264 minus leader FWD | AAGGACACACATATTATTGATAAAAAG |
| PF11_0264 REV | TCAGCTAAAAAAGTAAAGACTATCC |
| PF14_0061 minus leader +T7 FWD | GCCATAATACGACTCACTATAGGACAACTTTCTGAACG |
| PF14_0061 minus leader FWD | GACAACTTTCTGAACG |
| PF14_0061 REV | ATAGTTTTCGGTTTCGG |
| PF14_0061_Nterm Fwd | AAGTTCTGTTTCAGGGCCCGGACAACTTTCTGAACG |
| PF14_0061_Nterm Rev | ATGGTCTAGAAAGCTTTAATAGTTTTCGGTTTCGG |
| pGEM FWD | TGCAAGGCGATTAAGTTGGGT |
| pGEM REV | TGTGGAATTGTGAGCGGATAAC |
| rpl2 REV | TCCACCTCCTTTATTATAAATAG |
| rpoB REV | GAATACATGTTTTATATAATCC |
| rps2 REV | TATGAACCAATTATTTTAGGTA |
| tufA + T7 FWD | GCCATAATACGACTCACTATAGAATGTACAAAAAGTAGCTATA CCT |
| tufA FWD | AATGTACAAAAAGTAGCTATACCT |

**A**

*Plasmodium*

The sequence name: **5866954**
Per-protein P-value for being PPR: **2.0E-42**
Probability for being PPR: **100.00%**

| Repeat | Begin | Alignment | End | P-value |
|---|---|---|---|---|
| PPR | 167 | -LAFNAAMSAVEKKGCLSTMLDLIGTMKSKNIKPDL-- | 201 | 2.6e-08 |
| PPR | 202 | -VSYKLVLSLCDKYHLVDTAEILFEEMIESDKINPN-- | 236 | 6.3e-09 |
| PPR | 238 | -EIYAIMISCYAKTGNGYKAIELFEKLRNDPFVEEM-- | 272 | 1.5e-09 |
| PPR | 338 | -SEYANVIYACNISNLYEQGIKYFEELLKSGKYMPS-- | 372 | 4.0e-07 |
| PPR | 374 | -FVFENIFDLLSKNGDYEKSLEYYNNLKNDPNFKKN-- | 408 | 2.5e-06 |
| PPR | 412 | -NILNNLLKALSIHNKINVAEDIWNNEFDELLLTPN-- | 446 | 1.5e-04 |
| PPR | 448 | -LSYQILLKIYSHIDNYEKAFKLFKEMQVNKLLNNK-- | 482 | 1.0e-09 |
| PPR | 485 | -LPFIYTIESTKNCGIYNYAIYVLRVAKLLNFKAND-- | 519 | 1.0e-02 |
| PPR | 522 | -MLYNNTMISCINSKKYDVIISLYAELINMQQKDTS-- | 556 | 1.1e-03 |
| PPR | 562 | -NTLTFVLLAFKELNMKQDFINLKNIIIQRNYKLPP-- | 596 | 5.0e-04 |

If you use TPRpred for your research, please cite:

> A Completely Reimplemented MPI Bioinformatics Toolkit with a New HHpred Server at its Core.
> Zimmermann L, Stephens A, Nam SZ, Rau D, Kübler J, Lozajic M, Gabler F, Söding J, Lupas AN, Alva V. J Mol Biol. 2018 Jul 20. S0022-2836(17)30587-9.

The sequence name: **Toxoplasma**
Per-protein P-value for being PPR: **1.3E-19**
Probability for being PPR: **100.00%**

| Repeat | Begin | Alignment | End | P-value |
|---|---|---|---|---|
| PPR | 336 | -AAYNAALHACERQRDRPGALRIYAAMREKEIPIDV-- | 370 | 2.5e-09 |
| PPR | 371 | -VTLHSLFTLLEAFADDTALLQILAQVDSADPESSM-- | 405 | 3.7e-03 |
| PPR | 411 | -SPSSSLSPSPRSDSQASPTVSVTPSLLSLGISTCC-- | 445 | 3.0e-01 |
| PPR | 454 | -VQLMERLKVLLRRNADKFLLNFTSVLPPDTADATA-- | 488 | 6.6e-01 |
| PPR | 496 | -GVYVQLVVALTQEGRYEEALAYYEELKSLQRRFVE-- | 530 | 5.0e-07 |
| PPR | 585 | -GAVNAALEACLYTGRLKQALLIYKEDVEFPEKQRL-- | 619 | 4.3e-03 |
| PPR | 646 | -RACSQQRQAFALAQIWRDFEELQATVAETQNASPF-- | 680 | 8.4e-02 |
| PPR | 687 | -PCVASAIQGFAACGLWTYALRLLLLLHKETGDARQ-- | 721 | 3.5e-05 |
| PPR | 726 | -LLSDLLRDAREAAERSGAARETQSKMRSENEGERS-- | 760 | 7.2e-02 |
| PPR | 792 | -EPYLAVLRACRDVGAWKPALGILRLLQQRHTQAKL-- | 826 | 6.4e-04 |

**B**

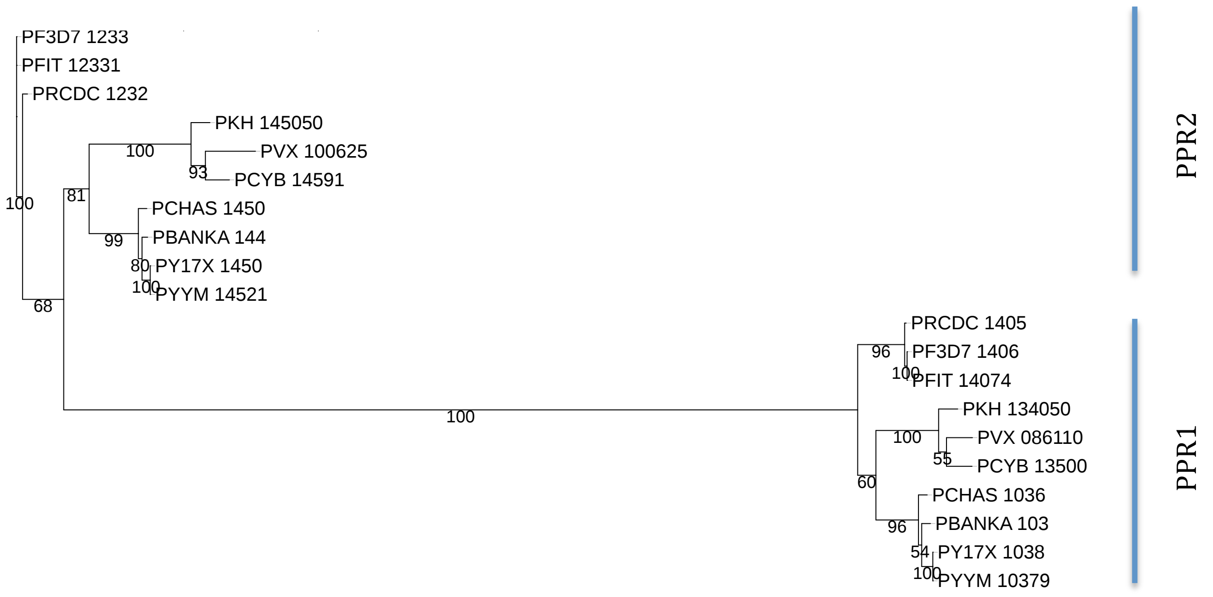


**Supplemental Figure S1.** PPR proteins in *Plasmodium* and *Toxoplasma.* A. PPR predictions for *Plasmodium falciparum* and *Toxoplasma gondii* PPR1. B. PPR1 and PPR2 proteins across the *Plasmodium* species. Mature (i.e. without targeting sequence) PPR1 (apicoplast) and PPR2 (mitochondrial) were aligned across selected *Plasmodium* species using ClustalW, and a phylogenetic tree inferred using PhyML.

Key:
PF3D7: *P. falciparum 3D7* (PF14_0061 and PF3D7_1233300),
PFIT: *P. falciparum* IT (PFIT_1407400 and PfIT_120038300),
PRCDC: *P. reichenowi*, (PRCDC_1232600 and PRCDC_1405700),
PKH: *P. knowlesi* strain H (PKH_13050 and PKNH_1452800),
PVH: *P. vivax (*PVX_086110 and PVX_100625),
PCYB: *P. cynmolgi* Strain B (PCYB_135000 and PCYB_145910),
PBANKA: *P.berghei* ANKA (PBANKA_103580 and PBANKA_1448000),
PY17X *P. yoelii yoelii* 17X (PY17X_1038200 and PY17X_1450500),
PYYM: *P. yoelii yoelii* YM (PYYM_1037900 and PYYM_1452100).

**Supplemental Figure 2.** Alignment of PPR1 proteins from *Plasmodium*, *Toxoplasma* and PPR10 from *Arabidopsis thaliana*. *Plasmodium* species as in Supplemental Figure 1.

```
AthPPR10           ------------------------------------------------------------   0
Toxoplasma         HFAFASFEKAKGSEKCEPAKTPRPTVETADGEPLRRDPADPSSGSARVCGVHTSGAPLAT   60
PVX_086110         ------------------------------------------------------------   0
PKH_134050         ------------------------------------------------------------   0
C922_03803         ------------------------------------------------------------   0
PCYB_135000        ------------------------------------------------------------   0
PRCDC_1405700      ------------------------------------------------------------   0
PF3D7_1406400      ------------------------------------------------------------   0
PFFVO_05018        ------------------------------------------------------------   0
PFIT_1407400       ------------------------------------------------------------   0
PFTANZ_05018       ------------------------------------------------------------   0
PCHAS_103660       ------------------------------------------------------------   0
PY17X_1038200      ------------------------------------------------------------   0
PY03222            ------------------------------------------------------------   0
PYYM_1037900       ------------------------------------------------------------   0
PBANKA_103580      ------------------------------------------------------------   0

AthPPR10           ------------------------------------------------------------   0
Toxoplasma         VPKANNESCEGKRHFRFRTNDEEGNHGNELEGRFEEPEEPEEPDRAAEGQEKRRRENSGK   120
PVX_086110         ------------------------------------------------------------   0
PKH_134050         ------------------------------------------------------------   0
C922_03803         ------------------------------------------------------------   0
PCYB_135000        ------------------------------------------------------------   0
PRCDC_1405700      ------------------------------------------------------------   0
PF3D7_1406400      ------------------------------------------------------------   0
PFFVO_05018        ------------------------------------------------------------   0
PFIT_1407400       ------------------------------------------------------------   0
PFTANZ_05018       ------------------------------------------------------------   0
PCHAS_103660       ------------------------------------------------------------   0
PY17X_1038200      ------------------------------------------------------------   0
PY03222            ------------------------------------------------------------   0
PYYM_1037900       ------------------------------------------------------------   0
PBANKA_103580      ------------------------------------------------------------   0

AthPPR10           ------------------------------------------------------------   0
Toxoplasma         TPGRATGKRKSLNGYHASWLASAFNRHQASHIHSLRVSSFRSPLSSFLSSSHSSSLALCS   180
PVX_086110         ------------------------------------------------------------   0
PKH_134050         ------------------------------------------------------------   0
C922_03803         ------------------------------------------------------------   0
PCYB_135000        ------------------------------------------------------------   0
PRCDC_1405700      ------------------------------------------------------------   0
PF3D7_1406400      ------------------------------------------------------------   0
PFFVO_05018        ------------------------------------------------------------   0
PFIT_1407400       ------------------------------------------------------------   0
PFTANZ_05018       ------------------------------------------------------------   0
PCHAS_103660       ------------------------------------------------------------   0
PY17X_1038200      ------------------------------------------------------------   0
PY03222            ------------------------------------------------------------   0
PYYM_1037900       ------------------------------------------------------------   0
PBANKA_103580      ------------------------------------------------------------   0
```

```
AthPPR10           --------------------------------------------------ASRRDELLRA   10
Toxoplasma         SSFSPFASSLLSPSPLFLNPASSASGRRVSLSNFLSSPSSSSPSVSASSSCSSSSSLLRA   240
PVX_086110         ------------------------------------------------------------   0
PKH_134050         ------------------------------------------------------------   0
C922_03803         ------------------------------------------------------------   0
PCYB_135000        ------------------------------------------------------------   0
PRCDC_1405700      ------------------------------------------------------------   0
PF3D7_1406400      ------------------------------------------------------------   0
PFFVO_05018        ------------------------------------------------------------   0
PFIT_1407400       ------------------------------------------------------------   0
PFTANZ_05018       ------------------------------------------------------------   0
PCHAS_103660       ------------------------------------------------------------   0
PY17X_1038200      ------------------------------------------------------------   0
PYO3222            ------------------------------------------------------------   0
PYYM_1037900       ------------------------------------------------------------   0
PBANKA_103580      ------------------------------------------------------------   0

AthPPR10           D----------------------------------------ITSLLKALELSGHWEWALA   30
Toxoplasma         LVACHSSPQTASVSLSPPRGSSFSSLSPSVLLRRSETRTQAPTLQAPLLSVRGS------   294
PVX_086110         -----------------------------------------------DSPPDGD------   7
PKH_134050         -----------------------------------------------AAIHDED------   7
C922_03803         -----------------------------------------------TATPDED------   7
PCYB_135000        -----------------------------------------------GATPDED------   7
PRCDC_1405700      -----------------------------------------------DNFLNEN------   7
PF3D7_1406400      -----------------------------------------------DNFLNEN------   7
PFFVO_05018        -----------------------------------------------DNFLNEN------   7
PFIT_1407400       -----------------------------------------------DNFLNEN------   7
PFTANZ_05018       -----------------------------------------------DNFLNEN------   7
PCHAS_103660       ------------------------------------------------NQNDDS------   6
PY17X_1038200      ------------------------------------------------NKNDDN------   6
PYO3222            ------------------------------------------------NKNDDN------   6
PYYM_1037900       ------------------------------------------------NKNDDN------   6
PBANKA_103580      ------------------------------------------------NRNDD-------   5

AthPPR10           LLRWAGKEGAADASALEMVVRALGREGQHDAVCALLDETPLPPGSRLDVRAYTTVLHALS   90
Toxoplasma         ---WPPDAGDDRET--EKRLSPLRI--DYRQAFE-LLENADPTGIKALTAAYNAALHACE   346
PVX_086110         ---ILKRKGAEKEV--EKSVVPLNM--DWVKVMN-LIYAS--RDVDATTLAFNAAMSAVE   57
PKH_134050         ---ILRKKGAQKEV--EKSVVPLNM--DWVKVMN-LIYAS--RDVDATTLAFNAAMSAVE   57
C922_03803         ---ILKKKGAQKEV--EQSVVPLNM--DWVKVMN-LIYAS--RDVDATTLAFNAAMSAVE   57
PCYB_135000        ---ILKKKGAQKEV--EKSVVPLNM--DWVKVMN-LIYAS--RDVDATTLAFNAAMSAVE   57
PRCDC_1405700      ---ILKKKSSEKEI--EQSLTPLNM--DWVKVMN-LIYSS--NDIDATTLAFNAAMSAVE   57
PF3D7_1406400      ---ILKKKSSEKEI--EQSLTPLNM--DWVKVMN-LIYSS--NDIDATTLAFNAAMSAVE   57
PFFVO_05018        ---ILKKKSSEKEI--EQSLTPLNM--DWVKVMN-LIYSS--NDIDATTLAFNAAMSAVE   57
PFIT_1407400       ---ILKKKSSEKEI--EQSLTPLNM--DWVKVMN-LIYSS--NDIDATTLAFNAAMSAVE   57
PFTANZ_05018       ---ILKKKSSEKEI--EQSLTPLNM--DWVKVMN-LIYSS--NDIDATTLAFNAAMSAVE   57
PCHAS_103660       ---ILKKKHLEKEI--EQSVIPLNM--DWVKVMN-LISSS--SDVNTTTLAFNAALSAVE   56
PY17X_1038200      ---ILKKKHSEKQI--EQSIIPLNI--DWIKVMN-LISSS--NDVNTTTLAFNAALSAVE   56
PYO3222            ---ILKKKHSEKQI--EQSIIPLNI--DWIKVMN-LISSS--NDVNTTTLAFNAALSAVE   56
PYYM_1037900       ---ILKKKHSEKQI--EQSIIPLNI--DWIKVMN-LISSS--NDVNTTTLAFNAALSAVE   56
PBANKA_103580      ---ILKKKYSEKEI--EQSIIPLNM--DWVKVMN-LISAS--NDVNTTTLAFNAALSAVE   55
                                   *  :  *    :   .   *       .    . *:.:.: * .

AthPPR10           RAGRYERALELFAELRRQGVAPTLVTYNVVLDVYGRMGRSWPRIVALLDEMR-AAGVEPD   149
Toxoplasma         RQRDRPGALRIYAAMREKEIPIDVVTLHSLFTLLEAFADDTA----LLQILAQVDSADPE   402
PVX_086110         KKGCLKSMLEVFEIMKKKNIKPDLVSYKLLLRLCANYHLGEHAEI-LFDEMVETDKLTPT   116
PKH_134050         KKGCLKSMLELFEVMKKKNIKPDLVSYKLLLRLCANYHLGDHAEI-LFDEMVETDKLTPT   116
C922_03803         KKGCLKSMLELFEIMKKKNIKPDLVSYKLLLRLCANYHLGEHAEI-LFDEMVETEKLTPT   116
PCYB_135000        KKGCLKSMLELFEIMKKKNIKPDLVSYKLLLRLCANYHLGEHAEI-LFDEMVETDKLTPT   116
PRCDC_1405700      KKGCLTTMLDLIGTMKSKNIKPDLVSYKLVLSLCDKYHLVDTAEI-LFEEMIESDKINPN   116
PF3D7_1406400      KKGCLSTMLDLIGTMKSKNIKPDLVSYKLVLSLCDKYHLVDTAEI-LFEEMIESDKINPN   116
PFFVO_05018        KKGCLSTMLDLIGTMKSKNIKPDLVSYKLVLSLCDKYHLVDTAEI-LFEEMIESDKINPN   116
PFIT_1407400       KKGCLSTMLDLIGTMKSKNIKPDLVSYKLVLSLCDKYHLVDTAEI-LFEEMIESDKINPN   116
PFTANZ_05018       KKGCLSTMLDLIGTMKSKNIKPDLVSYKLVLSLCDKYHLVDTAEI-LFEEMIESDKINPN   116
PCHAS_103660       KKGCLTSIIELFEIMKKKNIKPDLISYKLILTLCDKYHLAEYAEI-LFDEMTESDNIRPN   115
PY17X_1038200      KKGCLTSIIELFEIMKKKNIKPDLISYKLILSLCDKYHLAEYAEI-LFDEMVESDNIRPS   115
PYO3222            KKGCLTSIIELFEIMKKKNIKPDLISYKLILSLCDKYHLAEYAEI-LFDEMVESDNIRPS   115
PYYM_1037900       KKGCLTSIIELFEIMKKKNIKPDLISYKLILSLCDKYHLAEYAEI-LFDEMVESDNIRPS   115
PBANKA_103580      KKRCLTSIIELFEIMKKKNIKPDLISYKLILTLCDKYHLADYAEI-LFNEMVESDNIRPN   114
                   :       : :   :: : :   ::: : :: :             *:: :       *
```

```
AthPPR10           -------------------------------GFTASTVIAACCRDGLVDEAVAFFEDLKA    178
Toxoplasma         SSMAFLSRSPSSSLSPSPRSDSQASPTVSVTPSLLSLGISTCCRAGNATAAVQLMERLKV    462
PVX_086110         -------------------------------YEIYALMISCFAKVGDGHRAVEFVEKLRS    145
PKH_134050         -------------------------------YEIYALMINCFAKVGDGHKAVEFLEKLRS    145
C922_03803         -------------------------------YEIYALMISCFAKVGDGHKAVEFLEKLRS    145
PCYB_135000        -------------------------------YEIYALMISCFAKVGDGHKAVEFVEKLRS    145
PRCDC_1405700      -------------------------------YEIYAIMISCYAKTGNGYKAIELFEKLRN    145
PF3D7_1406400      -------------------------------YEIYAIMISCYAKTGNGYKAIELFEKLRN    145
PFFVO_05018        -------------------------------YEIYAIMISCYAKTGNGYKAIELFEKLRN    145
PFIT_1407400       -------------------------------YEIYAIMISCYAKTGNGYKAIELFEKLRN    145
PFTANZ_05018       -------------------------------YEIYAIMISCYAKTGNGYKAIELFEKLRN    145
PCHAS_103660       -------------------------------YEIYSIMISCFSKVGDGHKAIEFLEKLRN    144
PY17X_1038200      -------------------------------YEIYSIMISCFSKVGDGHKAIEFLEKLRN    144
PY03222            -------------------------------YEIYSIMISCFSKVGDGHKAIEFLEKLRN    144
PYYM_1037900       -------------------------------YEIYSIMISCFSKVGDGHKAIEFLEKLRN    144
PBANKA_103580      -------------------------------YEIYSIMISCFSKVGDGYKASEFLEKLRN    143
                                                      :  *   .: *    *  :.* *:

AthPPR10           RG---HAPCVVTYNALLQVFGKAGNYTEALRV--LGEMEQNG------------------    215
Toxoplasma         LLRRNADKFLLNFTSVLPPDTADA------------------------------------    486
PVX_086110         D------PLVGEVNNWG-EAGG---------------------VSGSGSADD--------    169
PKH_134050         D------PLVEGVNNWEVDGSGDGNGSAGRNVSNDGNVSNDGNVSDDGNVSDDGNVNRGE    199
C922_03803         D------PLVEEVNNWG-DRSGD--------------------TADRGDTAD-----RGD    173
PCYB_135000        D------PLVEEVNNWGGDSSGVGDSSGG------GDSSGGGDRSGAGDSSGDG---DGS    190
PRCDC_1405700      D------PLVEEMRSLNITNSNDN-------------------KENSNDLET--------    172
PF3D7_1406400      D------PFVEEMRSLNITNTNDN-------------------KENSNDLQT--------    172
PFFVO_05018        D------PFVEEMRSLNITNTNDN-------------------KENSNDLQT--------    172
PFIT_1407400       D------PFVEEMRSLNITNTNDN-------------------KENSNDLQT--------    172
PFTANZ_05018       D------PFVEEMRSLNITNTNDN-------------------KENSNDLQT--------    172
PCHAS_103660       D------PFVENFKEL---TSKYD-------------------KEKSNRWED--------    168
PY17X_1038200      D------PFVENIKEL---DSKYD-------------------KEKSNRWEN--------    168
PY03222            D------PFVENIKEL---DSKYD-------------------KEKSNRWEN--------    168
PYYM_1037900       D------PFVENIKEL---DSKYD-------------------KEKSNRWEN--------    168
PBANKA_103580      D------PFVENIKEL---NSKYD-------------------KEKSNRWDN--------    167
                            :

AthPPR10           -------------------------------------------CQPDAVTYNELAGTYA-    231
Toxoplasma         ------------------------------------------------------------    486
PVX_086110         RQHPAWRDSFAEEATTEEGAEAEGSTYTNQFKEITKKIKHIERGSS-KIQYSEYANVIFA    228
PKH_134050         RGNYLWKDHFDEQAATEEDAKAEGNAYTNQFKDITKKIKHIEKGSS-KIQYSEYTNVIFA    258
C922_03803         GGNPAWKDAFAEEAPTEEGAKAEGKTYTNEFKEITKKIKQIEKGSS-KIQYSEYTNVIFA    232
PCYB_135000        GENRVWKDAFAEEAATEEGAKEEGNNYTNQFKEITKKIKHIEKGSS-KIQFSEYTNVIFA    249
PRCDC_1405700      --SIIHNNIEDNNNN-NNNN---NNIYDDKFKHISNKIKNVENYSG-KIQYSEYANVIYA    225
PF3D7_1406400      --SIIHNNMEDNNNN-NNNN--DNNIYDDKFKHISNKIKNVENCSG-KIQYSEYANVIYA    226
PFFVO_05018        --SIIHNNMEDNNNN-NNNNNNDNNIYDDKFKHISNKIKNVENCSG-KIQYSEYANVIYA    228
PFIT_1407400       --SIIHNNMEDNNNN-NNNNNNDNNIYDDKFKHISNKIKNVENCSG-KIQYSEYANVIYA    228
PFTANZ_05018       --SIIHNNMEDNNNN-NNNNN-DNNIYDDKFKHISNKIKNVENCSG-KIQYSEYANVIYA    227
PCHAS_103660       --TFAQVNS-------NNGEDNTPNYYNAQFKELTEKIKNVENNNN-KIQYSEYANVIFA    218
PY17X_1038200      --TFAQVNSKNDDNKINNGEDDSPNYYNTQFKELTEKIKNVENNNNNKIQYSEYANVIFA    226
PY03222            --TFAQVNSKNDDNKINNGEDDSPNYYNTQFKELTEKIKNVENNNNNKIQYSEYANVIFA    226
PYYM_1037900       --TFAQVNSKNDDNKINNGEDDSPNYYNTQFKELTEKIKNVENNNNNKIQYSEYANVIFA    226
PBANKA_103580      --TFAQVKSKSEDNKINHGEDDSPNYYNTQFKELTEKIKNVENNN--KIQYSEYANVIFA    223


AthPPR10           --RAGFFEEAARCLD-TMASKGLLPNAFTYNTVMTAYGNVGKVDEALALFDQMKKTGFVP    288
Toxoplasma         ------------------TAAVAHPPTGVYVQLVVALTQEGRYEEALAYYEELKSLQRRF    528
PVX_086110         CNMSNLPEQGIKYFEELLNTGKYMPSTLLLESIFDLLAKNGNYEKCLDYYNKLKDDPNFK    288
PKH_134050         CNMSNLPEQGIKYFEELLNTGKYMPSTLLLESIFDLLAKNGKYEKCLDYYNKLKDDPNFK    318
C922_03803         CNMSNLPEQGIKYFEELLNTGKYMPSTLLLESIFDLLAKNGNYEKCLEYYNKLKDDPNFK    292
PCYB_135000        CNMSNLPEQGIKYFEELLNTGKYMPSTLLLESIFDLLAKNGKYEKSLEYYNKLKDDPNFK    309
PRCDC_1405700      CNISNLYEQGIKYFEELLKSGKYMPSIFVFENIFDLLSKNGDYEKSLEYYNNLKNDPNFK    285
PF3D7_1406400      CNISNLYEQGIKYFEELLKSGKYMPSIFVFENIFDLLSKNGDYEKSLEYYNNLKNDPNFK    286
PFFVO_05018        CNISNLYEQGIKYFEELLKSGKYMPSIFVFENIFDLLSKNGDYEKSLEYYNNLKNDPNFK    288
PFIT_1407400       CNISNLYEQGIKYFEELLKSGKYMPSIFVFENIFDLLSKNGDYEKSLEYYNNLKNDPNFK    288
PFTANZ_05018       CNISNLYEQGIKYFEELLKSGKYMPSIFVFENIFDLLSKNGDYEKSLEYYNNLKNDPNFK    287
PCHAS_103660       CNISNLHEQGIKYFEELLNSTKYIPSTFIFENIFDLLGKNGNYEKALDYYNKIKDDPNFK    278
PY17X_1038200      CNMSNLHEHGIKYFEELLNSTKYIPSTFIFENIFNLLGKNGNYEKALDYYNKIKDDPNFK    286
PY03222            CNMSNLHEHGIKYFEELLNSTKYIPSTFIFENIFNLLGKNGNYEKALDYYNKIKDDPNFK    286
PYYM_1037900       CNMSNLHEHGIKYFEELLNSTKYIPSTFIFENIFNLLGKNGNYEKALDYYNKIKDDPNFK    286
PBANKA_103580      CNISNLHEHGIKYFEELLNSTKYIPSTFIFENIFDLLGKNGNYEKALDYYNKIKDDPNFK    283
                                      :    *       :.    : *  ::.*  ::::*.
```

```
AthPPR10          NVNTYNLVLGMLGKKSRF----------TVMLEMLGEMSRSGCTPNRVTWNTMLAVCGKR   338
Toxoplasma        VEK-QQLLEREVARRAELVERELKNAPEQERKKLLGDIERQV------------------   569
PVX_086110        -KA-I-------------------------------------------------------   291
PKH_134050        -KY-I-------------------------------------------------------   321
C922_03803        -KY-I-------------------------------------------------------   295
PCYB_135000       -KY-I-------------------------------------------------------   312
PRCDC_1405700     -KN-I-------------------------------------------------------   288
PF3D7_1406400     -KN-I-------------------------------------------------------   289
PFFVO_05018       -KN-I-------------------------------------------------------   291
PFIT_1407400      -KN-I-------------------------------------------------------   291
PFTANZ_05018      -KN-I-------------------------------------------------------   290
PCHAS_103660      -KY-I-------------------------------------------------------   281
PY17X_1038200     -KY-I-------------------------------------------------------   289
PYO3222           -KY-I-------------------------------------------------------   289
PYYM_1037900      -KY-I-------------------------------------------------------   289
PBANKA_103580     -KY-I-------------------------------------------------------   286

AthPPR10          GMEDYVTRVLEGMRSCGVELSRDTYNTLIAAYGRCGSRTNAFKMYNEMTSAGFTPCITTY   398
Toxoplasma        ------------------------------------------VEEQELLLEDYQPPIGAV   587
PVX_086110        -------------------------------------------------------NVNIL   296
PKH_134050        -------------------------------------------------------NVNIL   326
C922_03803        -------------------------------------------------------NVNIL   300
PCYB_135000       -------------------------------------------------------NVNIL   317
PRCDC_1405700     -------------------------------------------------------NVNIL   293
PF3D7_1406400     -------------------------------------------------------NVNIL   294
PFFVO_05018       -------------------------------------------------------NVNIL   296
PFIT_1407400      -------------------------------------------------------NVNIL   296
PFTANZ_05018      -------------------------------------------------------NVNIL   295
PCHAS_103660      -------------------------------------------------------NVNIL   286
PY17X_1038200     -------------------------------------------------------NVNIL   294
PYO3222           -------------------------------------------------------NVNIL   294
PYYM_1037900      -------------------------------------------------------NVNIL   294
PBANKA_103580     -------------------------------------------------------NVNIL   291
                                                                          :

AthPPR10          NALLNVLSRQGDWSTAQSIVSKMRTKGFKPNEQSYSLLLQCYAKGGNVAGIAAIENEVYG   458
Toxoplasma        NAALEACLYTGRLKQALLIYK---------------------------------------   608
PVX_086110        NNILKALSVQGKINIIEDLWK---------------------------------------   317
PKH_134050        NNILKALCVHGKINIIEQVWK---------------------------------------   347
C922_03803        NNILKALSVHAKSNIIEDVWK---------------------------------------   321
PCYB_135000       NNILKALTVHGKINIIEQVWK---------------------------------------   338
PRCDC_1405700     NNLLKTLSIHNKINVAEDIWN---------------------------------------   314
PF3D7_1406400     NNLLKALSIHNKINVAEDIWN---------------------------------------   315
PFFVO_05018       NNLLKALSIHNKINVAEDIWN---------------------------------------   317
PFIT_1407400      NNLLKALSIHNKINVAEDIWN---------------------------------------   317
PFTANZ_05018      NNLLKALSIHNKINVAEDIWN---------------------------------------   316
PCHAS_103660      NNLLKSLSLSNKINIIENIWN---------------------------------------   307
PY17X_1038200     NNILKSLSLSNKINIIENIWN---------------------------------------   315
PYO3222           NNILKSLSLSNKINIIENIWN---------------------------------------   315
PYYM_1037900      NNILKSLSLSNKINIIENIWN---------------------------------------   315
PBANKA_103580     NNILKSLSLSNKINIIENIWN---------------------------------------   312
                  *  *:        .    : .

AthPPR10          SGAVFPSWVILRTLVIANFKCRRLDGMETAFQEVKARGYNPDLVIFNSMLSIYAKNGMYS   518
Toxoplasma        EDVEFPEKQRL------------------------------------------------R   620
PVX_086110        N-----------------------------------------------------------   318
PKH_134050        N-----------------------------------------------------------   348
C922_03803        N-----------------------------------------------------------   322
PCYB_135000       N-----------------------------------------------------------   339
PRCDC_1405700     N-----------------------------------------------------------   315
PF3D7_1406400     N-----------------------------------------------------------   316
PFFVO_05018       N-----------------------------------------------------------   318
PFIT_1407400      N-----------------------------------------------------------   318
PFTANZ_05018      N-----------------------------------------------------------   317
PCHAS_103660      N-----------------------------------------------------------   308
PY17X_1038200     N-----------------------------------------------------------   316
PYO3222           N-----------------------------------------------------------   316
PYYM_1037900      N-----------------------------------------------------------   316
PBANKA_103580     N-----------------------------------------------------------   313
                  .
```

```
AthPPR10            KATEVFDSIKRSGLSPDLITYNSLMDMYAKCSESWEAEKILNQLKCSQTMK----PDVVS   574
Toxoplasma          AGEALDQDLAPSKAAPTLRTFELLLRACSQQRQAFALAQIWRDFEELQAT-VAETQNASP   679
PVX_086110          -------EFDELMLTPNEISYGYMLKVYSIVDD---YEKAFKLFKEMQMKKMLSNKNIIP   368
PKH_134050          -------EFDELMLTPNAISYAYMLNAYSIVDD---YEKAFKLFKEMQMKKMLNNKNIIP   398
C922_03803          -------EFDDLMLTPNAISYAYMLNVYSIMDD---YEKAFKLFKEMQMKKLLNNKNIIP   372
PCYB_135000         -------EFDDLMLTPNAISYGFMLNAYSIVDD---YEKAFKLFKEMHMKKLLNKKNIIP   389
PRCDC_1405700       -------EFDELLLTPNNLSYQILLKIYSHIDN---YEKAFKLFKEMQINKLLNNKNILP   365
PF3D7_1406400       -------EFDELLLTPNNLSYQILLKIYSHIDN---YEKAFKLFKEMQVNKLLNNKNILP   366
PFFVO_05018         -------EFDELLLTPNNLSYQILLKIYSHIDN---YEKAFKLFKEMQVNKLLNNKNILP   368
PFIT_1407400        -------EFDELLLTPNNLSYQILLKIYSHIDN---YEKAFKLFKEMQVNKLLNNKNILP   368
PFTANZ_05018        -------EFDELLLTPNNLSYQILLKIYSHIDN---YEKAFKLFKEMQVNKLLNNKNILP   367
PCHAS_103660        -------EYDELLLTHNSVSYQIMLNVYGNIDD---YEKAFKLFKEMQIKKMLNKKNILP   358
PY17X_1038200       -------EYDELLLVQNSVSYQIMLNVYSNIDD---YEKAFKLFKEMQMKKMLNKKNILP   366
PYO3222             -------EYDELLLVQNSVSYQIMLNVYSNIDD---YEKAFKLFKEMQMKKMLNKKNILP   366
PYYM_1037900        -------EYDELLLVQNSVSYQIMLNVYSNIDD---YEKAFKLFKEMQMKKMLNKKNILP   366
PBANKA_103580       -------EYDELLLTQNSVSYQIMLNVYSNIDD---YEKSFKLFKEMQMKKMLNKKNILP   363
                           .           ::  ::   .   :     :  . ::  :        :

AthPPR10            Y---------NTVINGFCKQGLVKEAQRVLS------------------EMVADGMAPCA   607
Toxoplasma          FRLPSAAPCVASAIQGFAACGLWTYALRLLLLLHKETGDARQRHCELLSDLLRDAREAAE   739
PVX_086110          F---------VYTINAFKNCGIYNYAIYVLRVAKLLGV-SSED----LLKLYNDAMVACV   414
PKH_134050          F---------VYTIHAFKNCGIYNYAIYVLRVAKLLNV-SSED----LLKLYNDAMVACI   444
C922_03803          F---------VYTINAFKNCGIYNYAIYVLRVAKLLNV-SSED----LLKLYNDAMIACI   418
PCYB_135000         F---------VYTINAFKNCGIYNYAIYVLRVAKLLDV-FSED----LLKLYNNAMIACV   435
PRCDC_1405700       F---------IYTIESTKNCGIYNYAIYVLRVAKLLNF-KAND----LLMLYNNTMISCI   411
PF3D7_1406400       F---------IYTIESTKNCGIYNYAIYVLRVAKLLNF-KAND----LLMLYNNTMISCI   412
PFFVO_05018         F---------IYTIESTKNCGIYNYAIYVLRVAKLLNF-KAND----LLMLYNNTMISCI   414
PFIT_1407400        F---------IYTIESTKNCGIYNYAIYVLRVAKLLNF-KAND----LLMLYNNTMISCI   414
PFTANZ_05018        F---------IYTIESTKNCGIYNYAIYVLRVAKLLNF-KAND----LLMLYNNTMISCI   413
PCHAS_103660        F---------VYVLNSFKNCGIYSYSIYVLRIAKLIGI-VGKD----LLFLYNNAMITCV   404
PY17X_1038200       F---------VYTINSFKNCGIYNYSIYVLRIAKLIGI-VGKD----LLFLYNNAMISCI   412
PYO3222             F---------VYTINSFKNCGIYNYSIYVLRIAKLIGI-VGKD----LLFLYNNAMISCI   412
PYYM_1037900        F---------VYTINSFKNCGIYNYSIYVLRIAKLIGI-VGKD----LLFLYNNAMISCI   412
PBANKA_103580       F---------VYTINSFKNCGIYNYSIYVLRIAKLIGI-VGKN----ILFLYNNAMIACI   409
                    :           .:..    *: . :  :*                    :  :    .

AthPPR10            V----------------------------------------------------TYHTLVG   615
Toxoplasma          RSGAARETQSKMRSENEGERSSEQKDGDARAAASESGDWERCLEGRFVDLGVEPYLAVLR   799
PVX_086110          NAK---KYDVVI--SLYAELITMQEKGAPS-----------------LEISISTLGFVLL   452
PKH_134050          NSK---KYEVVI--SLYAELVTMQEKGAPS-----------------LEINISTLGFVLL   482
C922_03803          SCK---KYDVVI--SLYAELITMQEKGAPS-----------------LEINISTLGFVLL   456
PCYB_135000         NCK---KYDVVI--SLYAELITMQEKGAPS-----------------LEINISTLGFVLL   473
PRCDC_1405700       NSK---KYDVII--SLYAELINMQQK-DTS-----------------FQININTLTFVLL   448
PF3D7_1406400       NSK---KYDVII--SLYAELINMQQK-DTS-----------------FQININTLTFVLL   449
PFFVO_05018         NSK---KYDVII--SLYAELINMQQK-DTS-----------------FQININTLTFVLL   451
PFIT_1407400        NSK---KYDVII--SLYAELINMQQK-DTS-----------------FQININTLTFVLL   451
PFTANZ_05018        NSK---KYDVII--SLYAELINMQQK-DTS-----------------FQININTLTFVLL   450
PCHAS_103660        NAK---KYDVII--SLYTELIALQEK-DTS-----------------LTININTLSFVLL   441
PY17X_1038200       NAK---KYDVII--SLYTELIALQEK-DTS-----------------LTININTLSFVLL   449
PYO3222             NAK---KYDVII--SLYTELIALQEK-DTS-----------------LTININTLSFVLL   449
PYYM_1037900        NAK---KYDVII--SLYTELIALQEK-DTS-----------------LTININTLSFVLL   449
PBANKA_103580       NAK---KYDVII--SLYTELIALQEK-DTS-----------------LIININTLSFVLL   446
                                                                         ::

AthPPR10            GYSSLEMFSEAREVIGYMVQHGLKPMELTYRRVV--------------------ESYCRA   655
Toxoplasma          ACRDVGAWKPALGILRLLQQRHTQAKLLHALGAARERREAERREQFQQRVALRRRLSEA   859
PVX_086110          AFRELDMREDFTNLKNLIIQKNYKLTPL--------------------------------   480
PKH_134050          AFKELNMRDDFINLKNLIIQKNYKLTPL--------------------------------   510
C922_03803          AFKELNMREDFTNLKNLIIQKNYKLTPL--------------------------------   484
PCYB_135000         AFKELNMREDFTNLKNLIIQKNYKLTPL--------------------------------   501
PRCDC_1405700       AFKELNMKQDFINLKNIIIQRNYKLPPL--------------------------------   476
PF3D7_1406400       AFKELNMKQDFINLKNIIIQRNYKLPPL--------------------------------   477
PFFVO_05018         AFKELNMKQDFINLKNIIIQRNYKLPPL--------------------------------   479
PFIT_1407400        AFKELNMKQDFINLKNIIIQRNYKLPPL--------------------------------   479
PFTANZ_05018        AFKELNMKQDFINLKNIIIQRNYKLPPL--------------------------------   478
PCHAS_103660        AFKELKMKEDFSNLKNLILQKNYKLTPL--------------------------------   469
PY17X_1038200       AFKELKMKEDFLNLKNIILQKNYKLTPL--------------------------------   477
PYO3222             AFKELKMKEDFLNLKNIILQKNYKLTPL--------------------------------   477
PYYM_1037900        AFKELKMKEDFLNLKNIILQKNYKLTPL--------------------------------   477
PBANKA_103580       AFKELKMKEDFSNLKNLILQKNYKLTPL--------------------------------   474
                    .  .:   .    :   : *:  :   *
```

```
AthPPR10           KRFEEARGFLSEVSETDLD-----------FDKKALEAYIE----DAQFGR---------   691
Toxoplasma         KRKTNGADCVGEAAEQQRPTSASPTGDSDPLLSRDMERFLGSLHNSVAVGRTPLLPEFPY   919
PVX_086110         --------CGQLVSEQPH------------------------------------------   490
PKH_134050         --------CGKLVNEQQND-----------------------------------------   521
C922_03803         --------CGKLVNEEPEW-----------------------------------------   495
PCYB_135000        --------CAKLVSEESDS-----------------------------------------   512
PRCDC_1405700      --------CSKIFSETENY*----------------------------------------   487
PF3D7_1406400      --------CSKIFSETENY-----------------------------------------   488
PFFVO_05018        --------CSKIFSETENY-----------------------------------------   490
PFIT_1407400       --------CSKIFSETENY*----------------------------------------   490
PFTANZ_05018       --------CSKIFSETENY-----------------------------------------   489
PCHAS_103660       --------CSKIINEPEDQ*----------------------------------------   480
PY17X_1038200      --------CSKVINEQES*-----------------------------------------   487
PYO3222            --------CSKVINEQES------------------------------------------   487
PYYM_1037900       --------CSKVINEQES------------------------------------------   487
PBANKA_103580      --------CSKVINEP--------------------------------------------   482
                                 *

AthPPR10           ------------------------------------------------------------   691
Toxoplasma         EAYALTLGTMAAARAWDRVLAVSSEFFSRRDSGACGSASLGADGPAGEANEERKPARRAV   979
PVX_086110         ------------------------------------------------------------   490
PKH_134050         ------------------------------------------------------------   521
C922_03803         ------------------------------------------------------------   495
PCYB_135000        ------------------------------------------------------------   512
PRCDC_1405700      ------------------------------------------------------------   487
PF3D7_1406400      ------------------------------------------------------------   488
PFFVO_05018        ------------------------------------------------------------   490
PFIT_1407400       ------------------------------------------------------------   490
PFTANZ_05018       ------------------------------------------------------------   489
PCHAS_103660       ------------------------------------------------------------   480
PY17X_1038200      ------------------------------------------------------------   487
PYO3222            ------------------------------------------------------------   487
PYYM_1037900       ------------------------------------------------------------   487
PBANKA_103580      ------------------------------------------------------------   482


AthPPR10           ------------------------------------------------------------   691
Toxoplasma         GFGGERDEGGQPERGAEGERRDAPLEIRKSVHAYRLMALMHLGRHEEVEAERRSLIRLTE   1039
PVX_086110         ------------------------------------------------------------   490
PKH_134050         ------------------------------------------------------------   521
C922_03803         ------------------------------------------------------------   495
PCYB_135000        ------------------------------------------------------------   512
PRCDC_1405700      ------------------------------------------------------------   487
PF3D7_1406400      ------------------------------------------------------------   488
PFFVO_05018        ------------------------------------------------------------   490
PFIT_1407400       ------------------------------------------------------------   490
PFTANZ_05018       ------------------------------------------------------------   489
PCHAS_103660       ------------------------------------------------------------   480
PY17X_1038200      ------------------------------------------------------------   487
PYO3222            ------------------------------------------------------------   487
PYYM_1037900       ------------------------------------------------------------   487
PBANKA_103580      ------------------------------------------------------------   482


AthPPR10           ------------------------------------------------------------   691
Toxoplasma         MERRTRERRDARQREREGGEEKREEGEGSEEAFLPKWREETEHEEGEGGEDEGSHGIATL   1099
PVX_086110         ------------------------------------------------------------   490
PKH_134050         ------------------------------------------------------------   521
C922_03803         ------------------------------------------------------------   495
PCYB_135000        ------------------------------------------------------------   512
PRCDC_1405700      ------------------------------------------------------------   487
PF3D7_1406400      ------------------------------------------------------------   488
PFFVO_05018        ------------------------------------------------------------   490
PFIT_1407400       ------------------------------------------------------------   490
PFTANZ_05018       ------------------------------------------------------------   489
PCHAS_103660       ------------------------------------------------------------   480
PY17X_1038200      ------------------------------------------------------------   487
PYO3222            ------------------------------------------------------------   487
PYYM_1037900       ------------------------------------------------------------   487
PBANKA_103580      ------------------------------------------------------------   482
```

```
AthPPR10          ------------------------------------------------------------   691
Toxoplasma        VLEEAETWLMRGVTRERRTQGKDNIYPTPSIHETTPHTYEATHHSFPSPSKSSSASSSAS   1159
PVX_086110        ------------------------------------------------------------   490
PKH_134050        ------------------------------------------------------------   521
C922_03803        ------------------------------------------------------------   495
PCYB_135000       ------------------------------------------------------------   512
PRCDC_1405700     ------------------------------------------------------------   487
PF3D7_1406400     ------------------------------------------------------------   488
PFFVO_05018       ------------------------------------------------------------   490
PFIT_1407400      ------------------------------------------------------------   490
PFTANZ_05018      ------------------------------------------------------------   489
PCHAS_103660      ------------------------------------------------------------   480
PY17X_1038200     ------------------------------------------------------------   487
PYO3222           ------------------------------------------------------------   487
PYYM_1037900      ------------------------------------------------------------   487
PBANKA_103580     ------------------------------------------------------------   482

AthPPR10          ------------------------------------------------------------   691
Toxoplasma        SSASSSASSSASSSSFSSASSSSFSSSPLQPSFIAGSGGGRSMSLRDVRHLEGVSAMSEG   1219
PVX_086110        ------------------------------------------------------------   490
PKH_134050        ------------------------------------------------------------   521
C922_03803        ------------------------------------------------------------   495
PCYB_135000       ------------------------------------------------------------   512
PRCDC_1405700     ------------------------------------------------------------   487
PF3D7_1406400     ------------------------------------------------------------   488
PFFVO_05018       ------------------------------------------------------------   490
PFIT_1407400      ------------------------------------------------------------   490
PFTANZ_05018      ------------------------------------------------------------   489
PCHAS_103660      ------------------------------------------------------------   480
PY17X_1038200     ------------------------------------------------------------   487
PYO3222           ------------------------------------------------------------   487
PYYM_1037900      ------------------------------------------------------------   487
PBANKA_103580     ------------------------------------------------------------   482

AthPPR10          ------------------------------------------------------------   691
Toxoplasma        LKEGRGEETSVARRAEFLIGGGTKRKVCVQEEDRGDAVAEGEANHKSVCRDETGEEHSGR   1279
PVX_086110        ------------------------------------------------------------   490
PKH_134050        ------------------------------------------------------------   521
C922_03803        ------------------------------------------------------------   495
PCYB_135000       ------------------------------------------------------------   512
PRCDC_1405700     ------------------------------------------------------------   487
PF3D7_1406400     ------------------------------------------------------------   488
PFFVO_05018       ------------------------------------------------------------   490
PFIT_1407400      ------------------------------------------------------------   490
PFTANZ_05018      ------------------------------------------------------------   489
PCHAS_103660      ------------------------------------------------------------   480
PY17X_1038200     ------------------------------------------------------------   487
PYO3222           ------------------------------------------------------------   487
PYYM_1037900      ------------------------------------------------------------   487
PBANKA_103580     ------------------------------------------------------------   482

AthPPR10          ------------------------------------------------------------   691
Toxoplasma        SLDRFFAGLEPVSEREARAVAEAVERRRTEEHWRQEKMQSGVKRATGDREGMQPKRSADP   1339
PVX_086110        ------------------------------------------------------------   490
PKH_134050        ------------------------------------------------------------   521
C922_03803        ------------------------------------------------------------   495
PCYB_135000       ------------------------------------------------------------   512
PRCDC_1405700     ------------------------------------------------------------   487
PF3D7_1406400     ------------------------------------------------------------   488
PFFVO_05018       ------------------------------------------------------------   490
PFIT_1407400      ------------------------------------------------------------   490
PFTANZ_05018      ------------------------------------------------------------   489
PCHAS_103660      ------------------------------------------------------------   480
PY17X_1038200     ------------------------------------------------------------   487
PYO3222           ------------------------------------------------------------   487
PYYM_1037900      ------------------------------------------------------------   487
PBANKA_103580     ------------------------------------------------------------   482
```

```
AthPPR10           ------------------------------------------------------------    691
Toxoplasma         EGDKNDPGCQTAGNDPGGETGLRRREGERDGVNEEAGGREKGASGQIEREREGEHDTEEA    1399
PVX_086110         ------------------------------------------------------------    490
PKH_134050         ------------------------------------------------------------    521
C922_03803         ------------------------------------------------------------    495
PCYB_135000        ------------------------------------------------------------    512
PRCDC_1405700      ------------------------------------------------------------    487
PF3D7_1406400      ------------------------------------------------------------    488
PFFVO_05018        ------------------------------------------------------------    490
PFIT_1407400       ------------------------------------------------------------    490
PFTANZ_05018       ------------------------------------------------------------    489
PCHAS_103660       ------------------------------------------------------------    480
PY17X_1038200      ------------------------------------------------------------    487
PYO3222            ------------------------------------------------------------    487
PYYM_1037900       ------------------------------------------------------------    487
PBANKA_103580      ------------------------------------------------------------    482

AthPPR10           ------------------------------------------------------------    691
Toxoplasma         RERLEEKEEEREKENSEREGRAIRAVSGERGLSVSAFLSSVLGPRRAVLESMREEPVLED    1459
PVX_086110         ------------------------------------------------------------    490
PKH_134050         ------------------------------------------------------------    521
C922_03803         ------------------------------------------------------------    495
PCYB_135000        ------------------------------------------------------------    512
PRCDC_1405700      ------------------------------------------------------------    487
PF3D7_1406400      ------------------------------------------------------------    488
PFFVO_05018        ------------------------------------------------------------    490
PFIT_1407400       ------------------------------------------------------------    490
PFTANZ_05018       ------------------------------------------------------------    489
PCHAS_103660       ------------------------------------------------------------    480
PY17X_1038200      ------------------------------------------------------------    487
PYO3222            ------------------------------------------------------------    487
PYYM_1037900       ------------------------------------------------------------    487
PBANKA_103580      ------------------------------------------------------------    482

AthPPR10           ------------------------------------------------------------    691
Toxoplasma         TVTEEPESLELHADAEAQLEAKQREGESRDRSGENTEASTERRNSPDREEEKQDPVTTAT    1519
PVX_086110         ------------------------------------------------------------    490
PKH_134050         ------------------------------------------------------------    521
C922_03803         ------------------------------------------------------------    495
PCYB_135000        ------------------------------------------------------------    512
PRCDC_1405700      ------------------------------------------------------------    487
PF3D7_1406400      ------------------------------------------------------------    488
PFFVO_05018        ------------------------------------------------------------    490
PFIT_1407400       ------------------------------------------------------------    490
PFTANZ_05018       ------------------------------------------------------------    489
PCHAS_103660       ------------------------------------------------------------    480
PY17X_1038200      ------------------------------------------------------------    487
PYO3222            ------------------------------------------------------------    487
PYYM_1037900       ------------------------------------------------------------    487
PBANKA_103580      ------------------------------------------------------------    482

AthPPR10           ------------------------------------------------------------    691
Toxoplasma         QETDANTGGTLSQAGGRKSEAASRSESVETSLDLLLRLKHKTPAETQQLEDGPPRWLARR    1579
PVX_086110         ------------------------------------------------------------    490
PKH_134050         ------------------------------------------------------------    521
C922_03803         ------------------------------------------------------------    495
PCYB_135000        ------------------------------------------------------------    512
PRCDC_1405700      ------------------------------------------------------------    487
PF3D7_1406400      ------------------------------------------------------------    488
PFFVO_05018        ------------------------------------------------------------    490
PFIT_1407400       ------------------------------------------------------------    490
PFTANZ_05018       ------------------------------------------------------------    489
PCHAS_103660       ------------------------------------------------------------    480
PY17X_1038200      ------------------------------------------------------------    487
PYO3222            ------------------------------------------------------------    487
PYYM_1037900       ------------------------------------------------------------    487
PBANKA_103580      ------------------------------------------------------------    482
```

```
AthPPR10          ------------------------------------------------------------   691
Toxoplasma        SGTGEPGAEAVAGSRRGVHPPDGRAGVGIPGGGAGSQRQRQETRGQSAFLFAGDHGWGWP   1639
PVX_086110        ------------------------------------------------------------   490
PKH_134050        ------------------------------------------------------------   521
C922_03803        ------------------------------------------------------------   495
PCYB_135000       ------------------------------------------------------------   512
PRCDC_1405700     ------------------------------------------------------------   487
PF3D7_1406400     ------------------------------------------------------------   488
PFFVO_05018       ------------------------------------------------------------   490
PFIT_1407400      ------------------------------------------------------------   490
PFTANZ_05018      ------------------------------------------------------------   489
PCHAS_103660      ------------------------------------------------------------   480
PY17X_1038200     ------------------------------------------------------------   487
PYO3222           ------------------------------------------------------------   487
PYYM_1037900      ------------------------------------------------------------   487
PBANKA_103580     ------------------------------------------------------------   482

AthPPR10          ---------------------------------------  691
Toxoplasma        PHWEEKLDKARARLQRTNDESGETEENAKTETPWGMRPT  1678
PVX_086110        ---------------------------------------  490
PKH_134050        ---------------------------------------  521
C922_03803        ---------------------------------------  495
PCYB_135000       ---------------------------------------  512
PRCDC_1405700     ---------------------------------------  487
PF3D7_1406400     ---------------------------------------  488
PFFVO_05018       ---------------------------------------  490
PFIT_1407400      ---------------------------------------  490
PFTANZ_05018      ---------------------------------------  489
PCHAS_103660      ---------------------------------------  480
PY17X_1038200     ---------------------------------------  487
PYO3222           ---------------------------------------  487
PYYM_1037900      ---------------------------------------  487
PBANKA_103580     ---------------------------------------  482
```

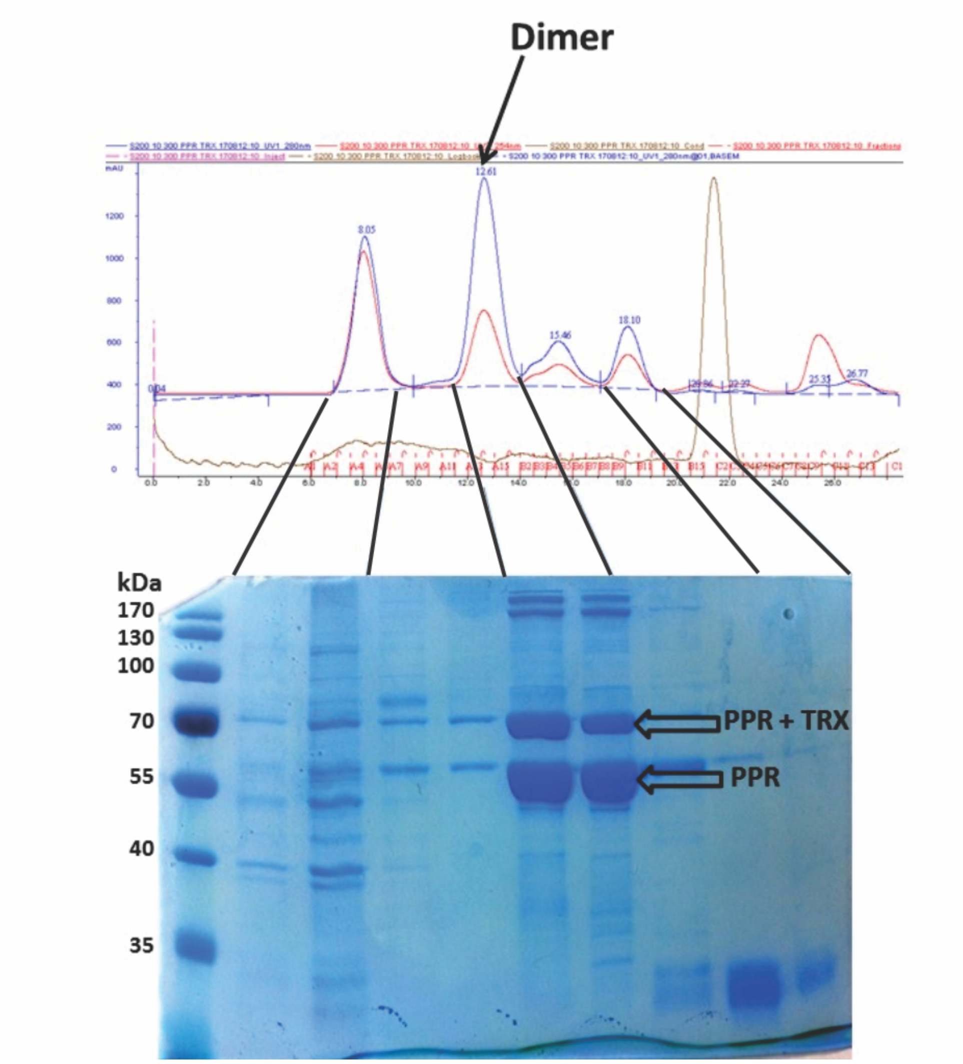


**Supplemental Figure S3. Gel filtration chromatography of His$_6$-TRX-PfPPR1 and corresponding SDS-PAGE gel**. Blue line represents absorbance at 280 nm, red line absorbance at 260 nm and brown line conductivity. The trace for absorbance at 280 nm (blue line) shows four major peaks eluted from the S200 10/300 column. Based on calibration of the S200 10/300 column, the second peak corresponds to a molecular weight of approximately 135 kDa, and the estimated molecular weight of the His$_6$-TRX-*Pf*PPR1 dimer is 141.6 kDa. Spontaneous cleavage of the TRX-Histag is observed in the second peak, corresponding to PPR protein without the His$_6$-TRX tag (estimated molecular weight of 56.8 kDa) with a smaller amount in the third peak. The fourth peak corresponds to the His$_6$-TRX tag only (14 kDa).

A B

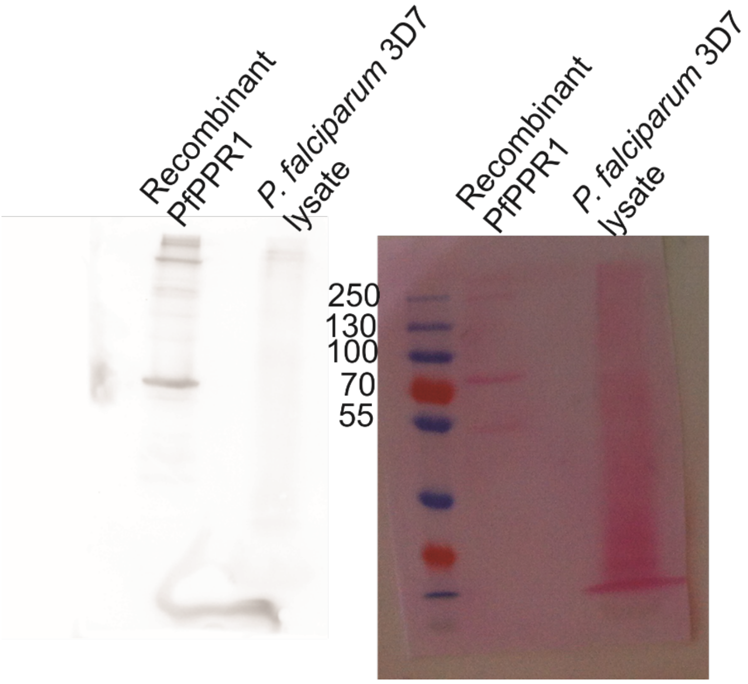


**Supplemental Figure S4. Western blot of purified *Pf*PPR1 protein and *P. falciparum* 3D7 lysate using the purified polyclonal anti-*Pf*PPR1 antibody.** Purified PfPPR1 protein was run on 10% SDS-PAGE gel along with *P. falciparum 3D7* cell lysate. After incubation with the purified *Pf*PPR1 polyclonal rabbit antibody and a secondary goat anti-rabbit antibody conjugated to HRP,  no *Pf*PPR1 protein could be detected in the *P. falciparum* 3D7 lysate (Panel A).. The positive control (recombinant *Pf*PPR1) shows a band of the correct size (*Pf*PPR1 + TRX $His_6$ ~ 72.8 kDa) .The ponceau stained gel (Panel B) shows good transfer of proteins of all molecular weights. Size markers are shown in kDa.

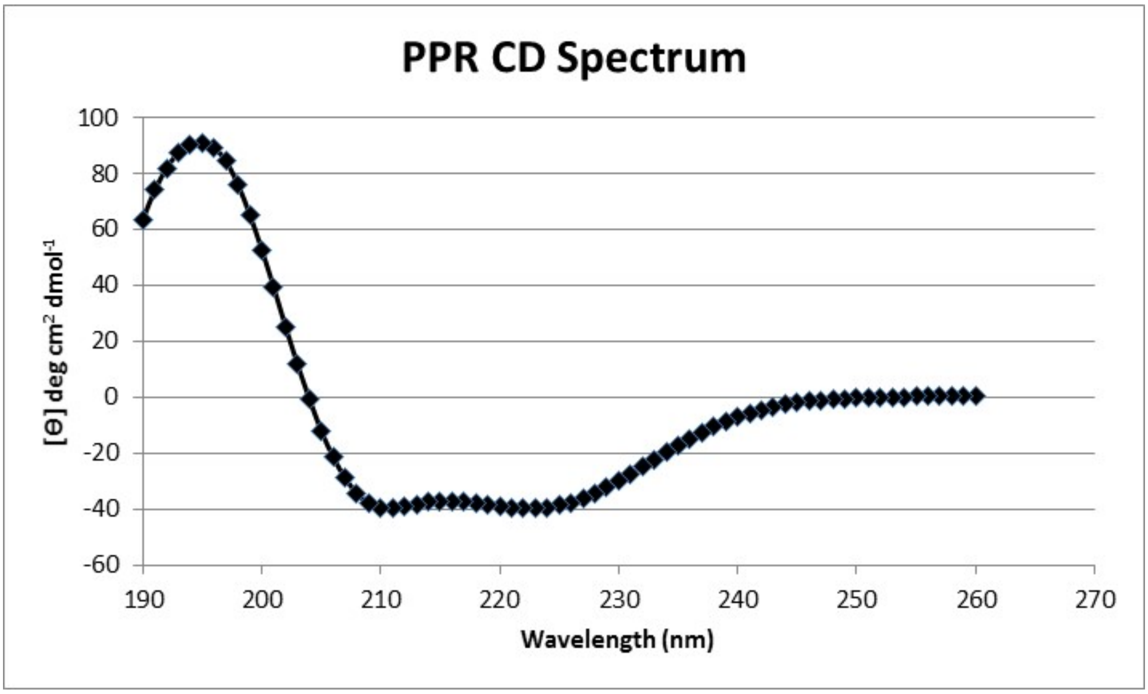


**Supplemental Figure S5. Circular Dichroism (CD) spectrum of purified *Pf*PPR1.**

*Pf*PPR1 minus His-TRX tag at 25°C in 10 mM potassium phosphate pH 8.0, 50 mM Na fluoride. Spectrum is typical of that for an alpha-helical protein.

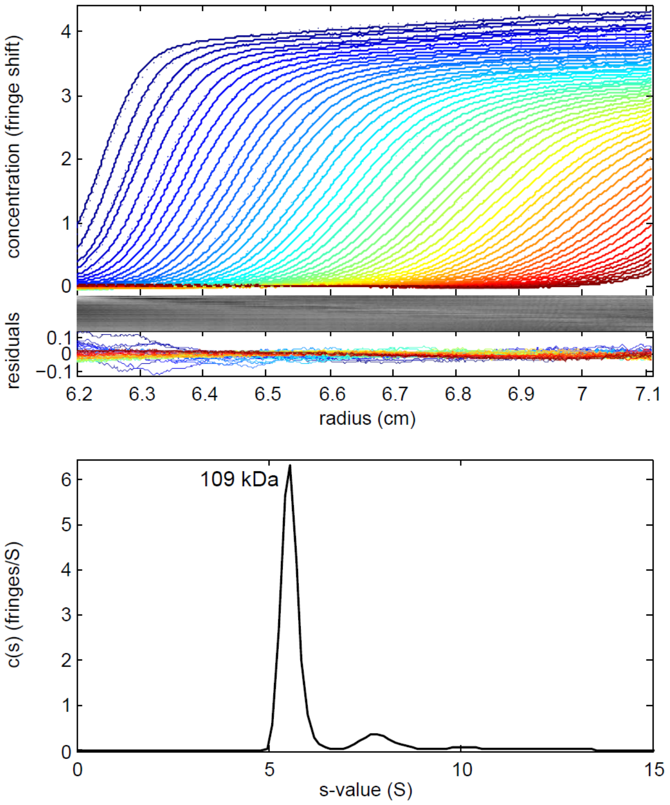


**Supplemental Figure S6. Analytical ultracentrifugation (AUC) sedimentation velocity data for *Pf*PPR1 minus $His_6$-TRX tag**. The residuals are from the fit with the continuous c(s) distribution model. Component sedimentation coefficient distribution for PPR at 1.8 mg/mL showing populations of dimeric (fitted mass of 109 kDa) and higher-order species, fitting to a uniform frictional ratio of Fk,w = 1.378. The r.m.s.d. was 0.016.

**Supplementary Figure S7**. SWISS-MODEL analysis of PPR1 from both *Toxoplasma* and *Plasmodium.*

*Plasmodium*

| Model #01 | File | Built with | Oligo-State | Ligands | GMQE | QMEAN |
|---|---|---|---|---|---|---|
| 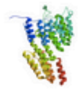 | PDB | ProMod3 Version 1.3.0. | monomer | None | 0.39 | -4.61 |

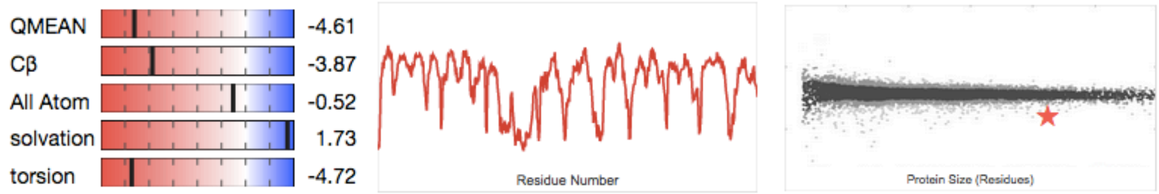


| Template | Seq Identity | Oligo-state | Found by | Method | Resolution | Seq Similarity | Range | Coverage | Description |
|---|---|---|---|---|---|---|---|---|---|
| 5i9f.1.A | 20.00 | monomer | HHblits | X-ray | 2.19Å | 0.31 | 160 - 584 | 0.63 | pentatricopeptide repeat protein dPPR-U10 |

The template contained no ligands.

*Toxoplasma*

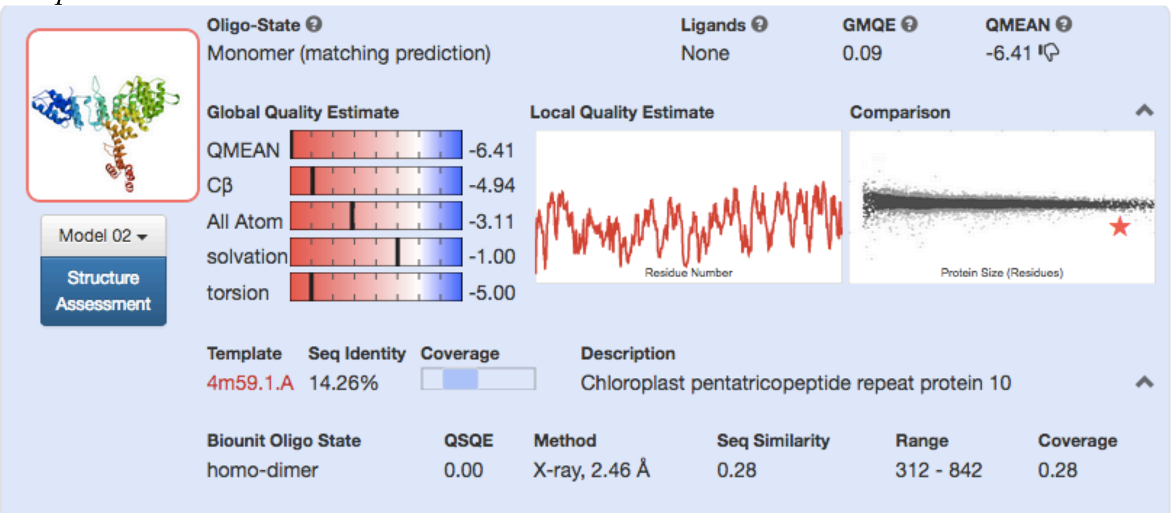

**Supplemental Figure S8. *Pf*PPR1 – RNA pull down assays.** (A) Five 150 nt RNA molecules (RNAs 1- 5, sequences as shown in Figure 3) and apicoplast RNA transcripts (*LSU rRNA – rpoB* and *tufA – clpC*) were used in a pull-down experiment. Biotinylated RNA was bound to streptavidin beads and used as 'bait' to pull down *Pf*PPR1 protein (with the TRX-$His_6$ tag removed). Bound *Pf*PPR1 was protein detected using a purified polyclonal anti-*Pf*PPR1 antibody from rabbit. Loading controls (PPR1 only) and a no RNA control reaction showed no non-specific binding in the absence of RNA. (B) The same pull down experiment using RNA oligonucleotides 1 – 3. The same result was obtained when the experiment was repeated (E). Details of RNA sequences are given in the legend to Figure 3, and specific sequences are shown (C and D).

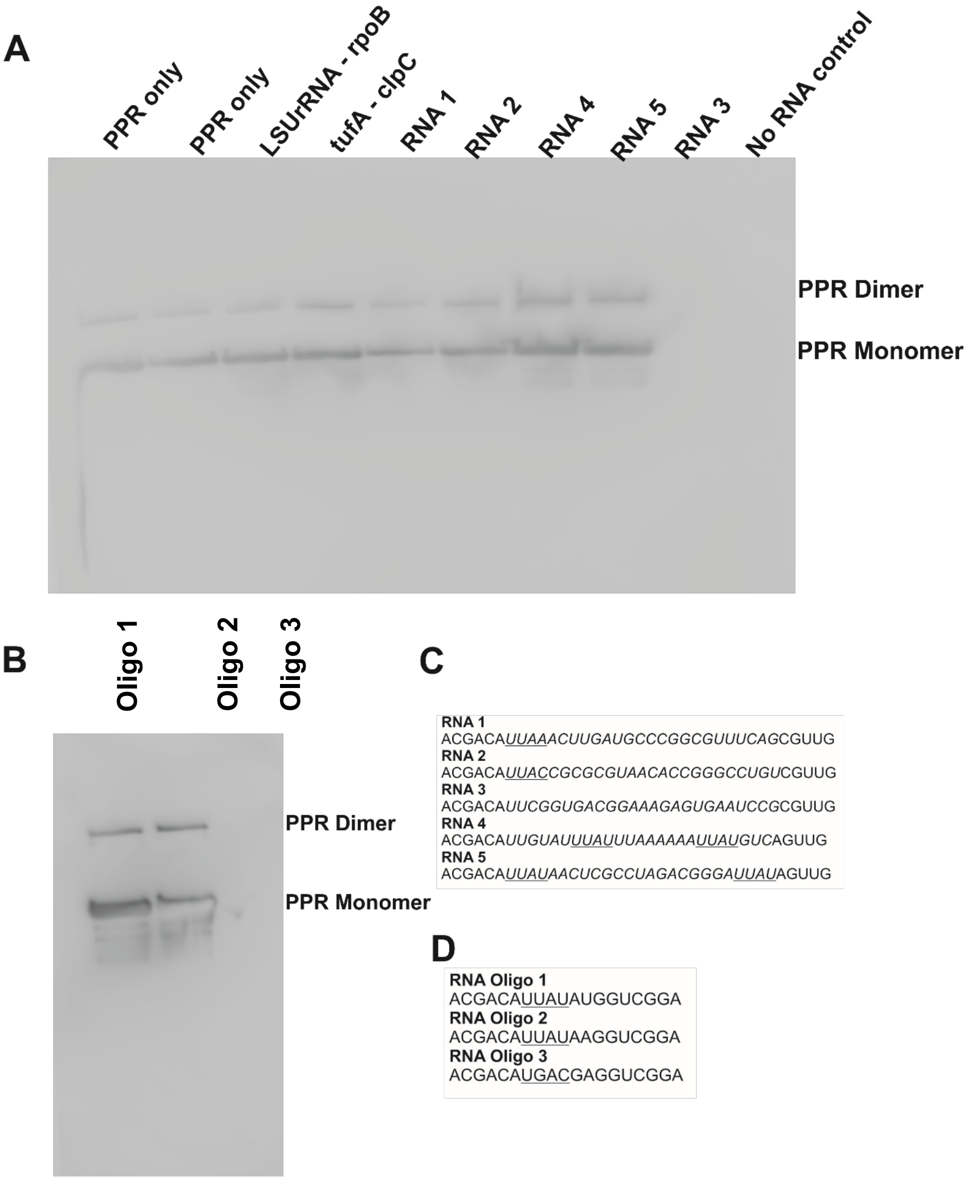

**E**

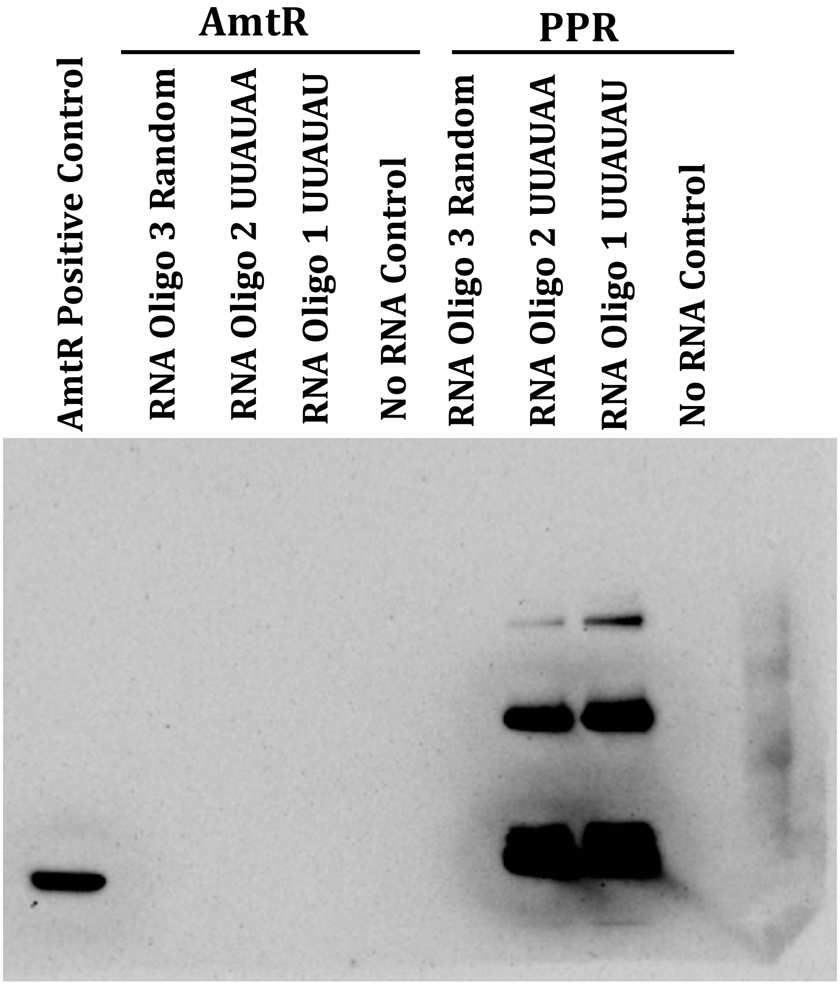
AmtR
PPR
AmtR Positive Control
RNA Oligo 3 Random
RNA Oligo 2 UUAUAA
RNA Oligo 1 UUAUAU
No RNA Control
RNA Oligo 3 Random
RNA Oligo 2 UUAUAA
RNA Oligo 1 UUAUAU
No RNA Control

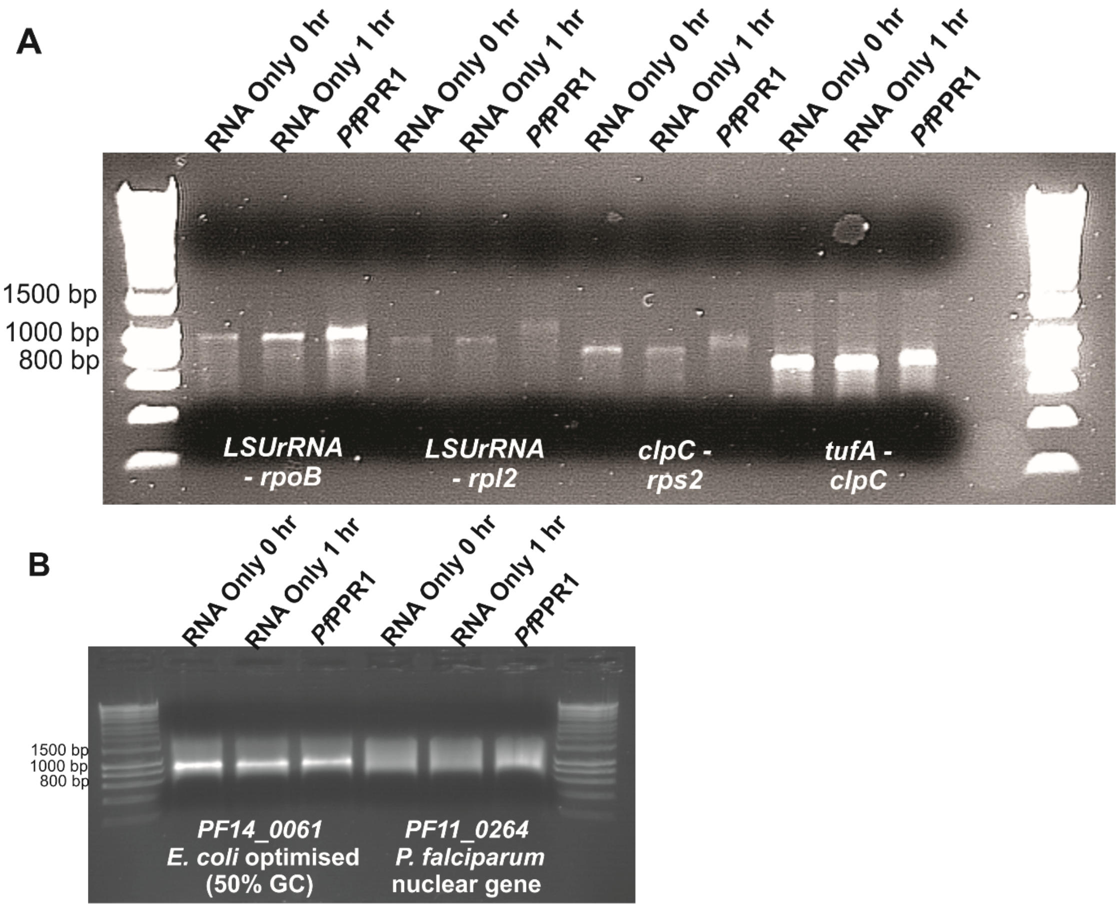


**Supplemental Figure S9. *Pf*PPR1 shows specific binding to apicoplast RNA transcripts.** *Pf*PPR1 binding to apicoplast RNA transcripts was tested in a gel shift experiment. Recombinant *Pf*PPR1 (minus TRX-$His_6$) causes a shift in the migration of *in vitro* transcribed apicoplast RNA molecules following incubation for one hour (Panel A). No shift is seen in the RNA if it is not bound to PPR1. Panel B shows that no shift is seen when PPR1 is incubated with *in vitro* transcribed RNA from a nuclear encoded *P. falciparum* gene (PF11_0264, or from an *E. coli* codon-optomized *P. falciparum* gene (PF14_0061) and a *P. falciparum 3D7* nuclear gene (PF11_0264).

**Supplemental Figure S10 Gel filtration shows a change in elution profile when *Pf*PPR1 is bound to RNA.** This is a repeat of the data shown in Figure 4, showing that the change in mobility following gel filtration is reproducible.

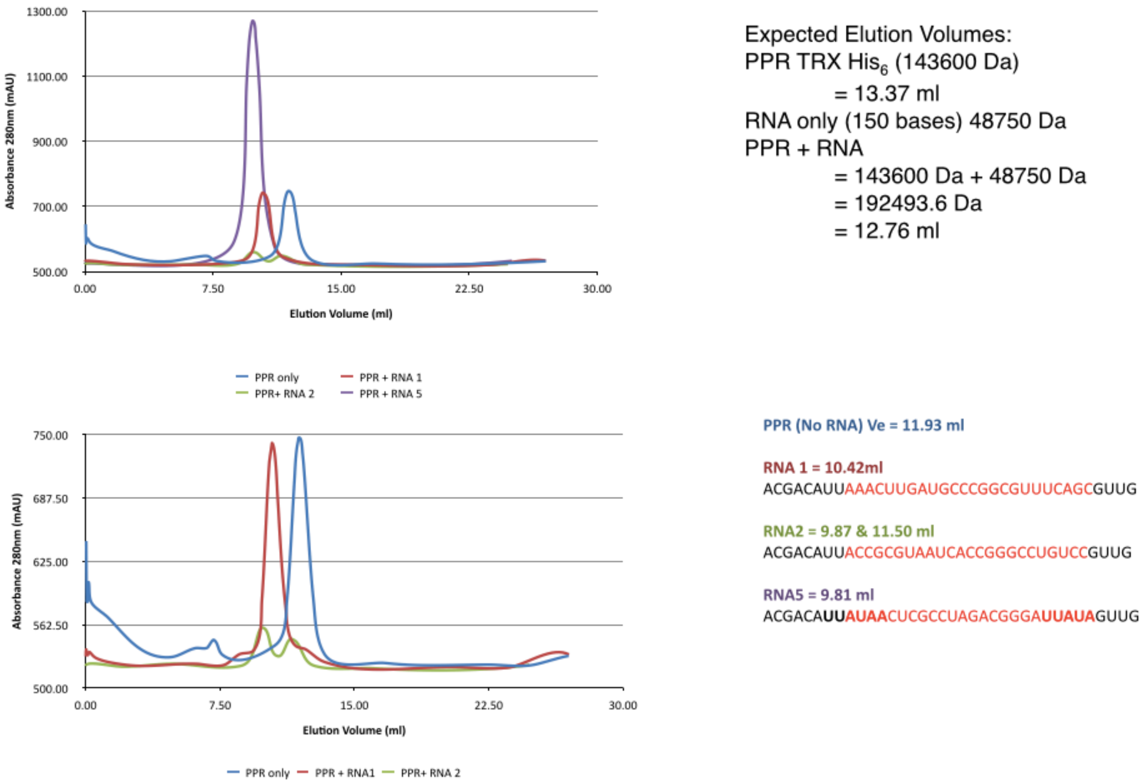

**Supplemental Figure 11. Ribonuclease A protection assays.** RNA transcripts 1, 4 and 5 and RNA oligos 1,2 and 3 were incubated in a 1:1 molar ratio with *Pf*PPR1 prior to treatment with RNase A. Samples were analyzed using a native acrylamide gel (ladder in nt). Experiments with no *Pf*PPR1 bound to RNA showed complete degradation by RNase A

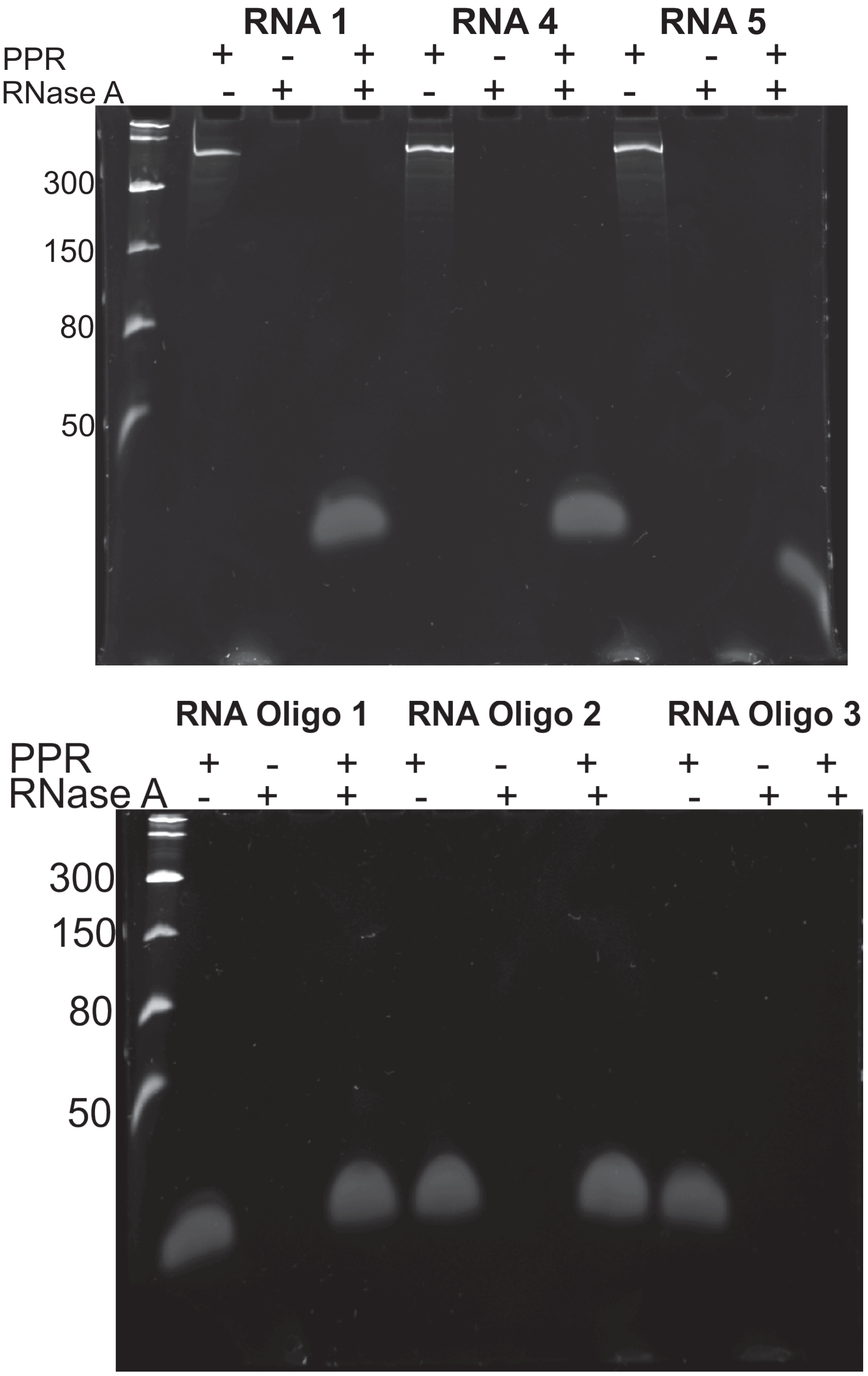

## Plasmid sequences used for *T. gondii* transformation.

**pCRISPR/Cas9-GFP_PPR-sgRNA**

*392-1148 SAG1 Promoter*
*1149-5022 Cas9-GFP coding sequence*
*6178-6490 SAG1 Terminator*
*6508-7053 U6 Promoter*
*7054-7170 sgRNA (7060-7079 PPR protospacer)*
*7420-8087 Col E1 origin*
*8235-9095 AMP*

```
   1 ctaaattgta agcgttaata ttttgttaaa attcgcgtta aatttttgtt aaatcagctc
  61 attttttaac caataggccg aaatcggcaa aatcccttat aaatcaaaag aatagaccga
 121 gatagggttg agtggccgct acagggcgct cccattcgcc attcaggctg cgcaactgtt
 181 gggaagggcg tttcggtgcg ggcctcttcg ctattacgcc agctggcgaa agggggatgt
 241 gctgcaaggc gattaagttg ggtaacgcca gggttttccc agtcacgacg ttgtaaaacg
 301 acggccagtg agcgcgacgt aatacgactc actatagggc gaattggcgg aaggccgtca
 361 aggcctaggc gcgccaggtc tcatgccgga gaagctttta catccgttgc cttttccacg
 421 gtccgtgatt tcatgtgcgt gcagcttcaa agactggtcg ttgcgactaa taagactgca
 481 gtgacaggtc gaatggtggg caccttgctg atgactatct actgcaaagt ctgagacaac
 541 gaacgaaact tcccacacga ggcatttgaa actgacggtg tctaggtaat atgcactgca
 601 agacacggta ctggggcctc gctgaattag ggccgatct cgttgcccta tcagtgctca
 661 cagtgccgca acgtaacacc agggcaggtt cttgacagtg gcaacaatgt gcgacgggcg
 721 tgtgaacgtt tcgtagtcat agcgctagca cgtacctagc cacatggtcg tgaggagctt
 781 taccatgcgt ctagaaggtg gatgcgggac acgccttcct ggcctttggc tcccgagacg
 841 cgtgttctaa ccacaaacct tgagacgcgt gttccaacca cgcaccctga cacgcgtgtt
 901 ccaaccacgc accctgagac gcgtgttcta accacgcacc ctgagacgcg tgttctaacc
 961 acgcaccctg agacgcgtgt tctgccgcac aatgtgcacc tgtaggaagc tgtagtcact
1021 gctgattctc actgttctcg gcaagggccg acgaccggag tacagttttt gtgggcagag
1081 ccgttgtgca gctttccgtt cttctcggtt gtgtcacatg tgtcattgtc gtgtaaacac
1141 acggttgtat gtcggtttcg ctgcaccact tcattatttc ttctggtttt ttgacgagta
1201 tgcatctaga caaaatggac aagaagtaca gcatcggcct ggacatcggc accaactctg
1261 tgggctgggc cgtgatcacc gacgagtaca aggtgcccag caagaaattc aaggtgctgg
1321 gcaacaccga ccggcacagc atcaagaaga acctgatcgg cgccctgctg ttcgacagcg
1381 gagaaacagc cgaggccacc cggctgaaga gaaccgccag aagaagatac accagacgga
1441 agaaccggat ctgctatctg caagagatct tcagcaacga gatggccaag gtggacgaca
1501 gcttcttcca cagactggaa gagtccttcc tggtggaaga ggataagaag cacgagcggc
1561 accccatctt cggcaacatc gtggacgagg tggcctacca cgagaagtac cccaccatct
1621 accacctgag aaagaaactg gtggacagca ccgacaaggc cgacctgcgg ctgatctatc
1681 tggccctggc ccacatgatc aagttccggg gccacttcct gatcgagggc gacctgaacc
1741 ccgacaacag cgacgtggac aagctgttca tccagctggt gcagacctac aaccagctgt
1801 tcgaggaaaa ccccatcaac gccagcggcg tggacgccaa ggccatcctg tctgccagac
1861 tgagcaagag cagacggctg gaaaatctga tcgcccagct gcccggcgag aagaagaatg
1921 gcctgttcgg caacctgatt gccctgagcc tgggcctgac ccccaacttc aagagcaact
1981 tcgacctggc cgaggatgcc aaactgcagc tgagcaagga cacctacgac gacgacctgg
2041 acaacctgct ggcccagatc ggcgaccagt acgccgacct gtttctggcc gccaagaacc
2101 tgtccgacgc catcctgctg agcgacatcc tgagagtgaa caccgagatc accaaggccc
2161 ccctgagcgc ctctatgatc aagagatacg acgagcacca ccaggacctg accctgctga
2221 aagctctcgt gcggcagcag ctgcctgaga agtacaaaga gattttcttc gaccagagca
2281 agaacggcta cgccggctac atcgatggcg gagccagcca ggaagagttc tacaagttca
2341 tcaagcccat cctggaaaag atggacggca ccgaggaact gctcgtgaag ctgaacagag
2401 aggacctgct gcggaagcag cggaccttcg acaacggcag catcccccac cagatccacc
2461 tgggagagct gcacgccatt ctgcggcggc aggaagattt ttacccattc ctgaaggaca
2521 accgggaaaa gatcgagaag atcctgacct tccgcatccc ctactacgtg ggccctctgg
2581 ccaggggaaa cagcagattc gcctggatga ccagaaagag cgaggaaacc atcacccccct
2641 ggaacttcga ggaagtggtg gacaagggcg ccagcgccca gagcttcatc gagcggatga
```

2701 ccaacttcga taagaacctg cccaacgaga aggtgctgcc caagcacagc ctgctgtacg
2761 agtacttcac cgtgtacaac gagctgacca aagtgaaata cgtgaccgag ggaatgagaa
2821 agcccgcctt cctgagcggc gagcagaaaa aagccatcgt ggacctgctg ttcaagacca
2881 accggaaagt gaccgtgaag cagctgaaag aggactactt caagaaaatc gagtgcttcg
2941 actccgtgga aatctccggc gtggaagatc ggttcaacgc ctccctgggc acataccacg
3001 atctgctgaa aattatcaag gacaaggact tcctggacaa tgaggaaaac gaggacattc
3061 tggaagatat cgtgctgacc ctgacactgt ttgaggacag agagatgatc gaggaacggc
3121 tgaaaaccta tgcccacctg ttcgacgaca aagtgatgaa gcagctgaag cggcggagat
3181 acaccggctg gggcaggctg agccggaagc tgatcaacgg catccgggac aagcagtccg
3241 gcaagacaat cctggatttc ctgaagtccg acggcttcgc caacagaaac ttcatgcagc
3301 tgatccacga cgacagcctg acctttaaag aggacatcca gaaagcccag gtgtccggcc
3361 agggcgatag cctgcacgag cacattgcca atctggccgg cagccccgcc attaagaagg
3421 gcatcctgca gacagtgaag gtggtggacg agctcgtgaa agtgatgggc cggcacaagc
3481 ccgagaacat cgtgatcgaa atggccagag agaaccagac cacccagaag ggacagaaga
3541 acagccgcga gagaatgaag cggatcgaag agggcatcaa agagctgggc agccagatcc
3601 tgaaagaaca ccccgtggaa aacacccagc tgcagaacga gaagctgtac ctgtactacc
3661 tgcagaatgg gcgggatatg tacgtggacc aggaactgga catcaaccgg ctgtccgact
3721 acgatgtgga ccatatcgtg cctcagagct ttctgaagga cgactccatc gataacaaag
3781 tgctgactcg gagcgacaag aaccggggca agagcgacaa cgtgccctcc gaagaggtcg
3841 tgaagaagat gaagaactac tggcgccagc tgctgaatgc caagctgatt acccagagga
3901 agttcgacaa tctgaccaag gccgagagag gcggcctgag cgaactggat aaggccggct
3961 tcatcaagag acagctggtg gaaacccggc agatcacaaa gcacgtggca cagatcctgg
4021 actcccggat gaacactaag tacgacgaga acgacaaact gatccgggaa gtgaaagtga
4081 tcaccctgaa gtccaagctg gtgtccgatt tccggaagga tttccagttt tacaaagtgc
4141 gcgagatcaa caactaccac cacgcccacg acgcctacct gaacgccgtc gtgggaaccg
4201 ccctgatcaa aaagtaccct aagctggaaa gcgagttcgt gtacggcgac tacaaggtgt
4261 acgacgtgcg gaagatgatc gccaagagcg agcaggaaat cggcaaggct accgccaagt
4321 acttcttcta cagcaacatc atgaactttt tcaagaccga gattaccctg gccaacggcg
4381 agatccggaa gcggcctctg atcgagacaa acggcgaaac aggcgagatc gtgtgggata
4441 agggccggga ctttgccacc gtgcggaaag tgctgtctat gccccaagtg aatatcgtga
4501 aaaagaccga ggtgcagaca ggcggcttca gcaaagagtc tatcctgccc aagaggaaca
4561 gcgacaagct gatcgccaga aagaaggact gggaccctaa gaagtacggc ggcttcgaca
4621 gccccaccgt ggcctattct gtgctggtgg tggccaaagt ggaaaagggc aagtccaaga
4681 aactgaagag tgtgaaagag ctgctgggga tcaccatcat ggaaagaagc agcttcgaga
4741 agaatcccat cgactttctg gaagccaagg gctacaaaga agtgaaaaag gacctgatca
4801 tcaagctgcc taagtactcc ctgttcgagc tggaaaacgg ccggaagaga atgctggcct
4861 ctgccggcga actgcagaag ggaaacgaac tggccctgcc ctccaaatat gtgaacttcc
4921 tgtacctggc cagccactat gagaagctga agggctcccc cgaggataat gagcagaaac
4981 agctgtttgt ggaacagcac aaacactacc tggacgagat catcgagcag atcagcgagt
5041 tctccaagag agtgatcctg gccgacgcta atctggacaa ggtgctgagc gcctacaaca
5101 agcacagaga caagcctatc agagagcagg ccgagaatat catccacctg tttaccctga
5161 ccaatctggg agcccctgcc gccttcaagt actttgacac caccatcgac cggaagaggt
5221 acaccagcac caaagaggtg ctggacgcca ccctgatcca ccagagcatc accggcctgt
5281 acgagacacg gatcgacctg tctcagctgg gaggcgacgc ctatccctat gacgtgcccg
5341 attatgccag cctgggcagc ggctccccca agaaaaaacg caaggtggaa gatcctaaga
5401 aaaagcggaa agtggacggc attggtagtg ggagcaacgg cagcagcgga tccgtgagca
5461 agggcgagga gctgttcacc ggggtggtgc ccatcctggt cgagctggac ggcgacgtaa
5521 acggccacaa gttcagcgtg cgcggcgagg gcgagggcga tgccaccaac ggcaagctga
5581 ccctgaagtt catctgcacc accggcaagc tgcccgtgcc ctggcccacc ctcgtgacca
5641 ccctgaccta cggcgtgcag tgcttcagcc gctaccccga ccacatgaag cagcacgact
5701 tcttcaagtc cgccatgccc gaaggctacg tccaggagcg caccatctcc ttcaaggacg
5761 acggcaccta caagacccgc gccgaggtga agttcgaggg cgacaccctg gtgaaccgca
5821 tcgagctgaa gggcatcgac ttcaaggagg acggcaacat cctggggcac aagctggagt
5881 acaacttcaa cagccacaac gtctatatca cggccgacaa gcagaagaac ggcatcaagg
5941 cgaacttcaa gatccgccac aacgtcgagg acggcagcgt gcagctcgcc gaccactacc
6001 agcagaacac ccccatcggc gacggccccg tgctgctgcc cgacaaccac tacctgagca
6061 cccagtccaa gctgagcaaa gaccccaacg agaagcgcga tcacatggtc ctgctggagt
6121 tcgtgaccgc cgccgggatc actctcggca tggacgagct gtacaagtag ttaattaatc
6181 accgttgtgc tcacttctca aatcgacaaa ggaaacacac ttcgtgcagc atgtgcccca
6241 ttataaagaa actgagttgt tccgctgtgg cttgcaggtg tcacatccac aaaaaccggc
6301 cgactctaaa taggagtgtt tcgcagcaag cagcgaaagt ttatgactgg gtccgaatct
6361 ctgaacggat gtgtggcgga cctggctgat gttgatcgcc gtcgacacac gcgccacatg
6421 ggtcaataca caagacagct atcagttgtt ttagtcgaac cggttaacac aattcttgcc

6481 cccccgaggg cgctgcaagg agtctcagga gcaagtaagc agaagcacgc tgtatttccg
6541 ggagggtgcg atgagacaaa gtgcgcgagt tgaaatcgtc gtggggacga tttcaccgcg
6601 gccacatgtt ggagacactg agggcacacg ggaaacgcga aagatttcaa attaacgtac
6661 ccaaacgcga aagcttgcgc agcatacact cgaagcgaac atcccgaacc atcgagaggc
6721 ggagagcgat aagtctttca cgctgcgaag tgttgcgacg gctgcgccgc tgcactgtga
6781 attgggcgcc aatattgcat cctaggcctg acgcgcctcc tgcagaacgc gagacactgg
6841 gatatgtaga gccaaggggg aaaccttcga actctcgaat gtattctctg acaagaatca
6901 tatttccatc agttctgtca gattttcaaa tggcgacctg cagaggcctg cttcctccct
6961 gtgcgctctt cgaaggggct ttctgtcgcg cagggtcacc tcgtccccga agggggtgtt
7021 tgccttctgg taaatgggga tgtcaagtta gttgagttgt tgagcgagag acagctcagg
7081 ttttagagct agaaatagca agttaaaata aggctagtcc gttatcaact tgaaaaagtg
7141 gcaccgagtc ggtgcttttt tgagctccag cttttgttcc ctttagtgag ggttaattgc
7201 gcgcttggcg tcgctactag aggatgcaca tgtgaccgag ggaattaatt aactggcctc
7261 atgggccttc cgctcactgc ccgctttcca gtcgggaaac ctgtcgtgcc agctgcatta
7321 acatggtcat agctgtttcc ttgcgtattg ggcgctctcc gcttcctcgc tcactgactc
7381 gctgcgctcg gtcgttcggg taaagcctgg ggtgcctaat gagcaaaagg ccagcaaaag
7441 gccaggaacc gtaaaaaggc cgcgttgctg gcgtttttcc ataggctccg cccccctgac
7501 gagcatcaca aaaatcgacg ctcaagtcag aggtggcgaa acccgacagg actataaaga
7561 taccaggcgt ttccccctgg aagctccctc gtgcgctctc ctgttccgac cctgccgctt
7621 accggatacc tgtccgcctt tctcccttcg ggaagcgtgg cgctttctca tagctcacgc
7681 tgtaggtatc tcagttcggt gtaggtcgtt cgctccaagc tgggctgtgt gcacgaaccc
7741 cccgttcagc ccgaccgctg cgccttatcc ggtaactatc gtcttgagtc caacccggta
7801 agacacgact tatcgccact ggcagcagcc actggtaaca ggattagcag agcgaggtat
7861 gtaggcggtg ctacagagtt cttgaagtgg tggcctaact acggctacac tagaagaaca
7921 gtatttggta tctgcgctct gctgaagcca gttaccttcg gaaaaagagt tggtagctct
7981 tgatccggca aacaaaccac cgctggtagc ggtggttttt ttgtttgcaa gcagcagatt
8041 acgcgcagaa aaaaaggatc tcaagaagat cctttgatct tttctacggg gtctgacgct
8101 cagtggaacg aaaactcacg ttaagggatt ttggtcatga gattatcaaa aaggatcttc
8161 acctagatcc ttttaaatta aaaatgaagt tttaaatcaa tctaaagtat atatgagtaa
8221 acttggtctg acagttacca atgcttaatc agtgaggcac ctatctcagc gatctgtcta
8281 tttcgttcat ccatagttgc ctgactcccc gtcgtgtaga taactacgat acgggagggc
8341 ttaccatctg gccccagtgc tgcaatgata ccgcgagaac cacgctcacc ggctccagat
8401 ttatcagcaa taaaccagcc agccggaagg gccgagcgca gaagtggtcc tgcaacttta
8461 tccgcctcca tccagtctat taattgttgc cgggaagcta gagtaagtag ttcgccagtt
8521 aatagtttgc gcaacgttgt tgccattgct acaggcatcg tggtgtcacg ctcgtcgttt
8581 ggtatggctt cattcagctc cggttcccaa cgatcaaggc gagttacatg atcccccatg
8641 ttgtgcaaaa aagcggttag ctccttcggt cctccgatcg ttgtcagaag taagttggcc
8701 gcagtgttat cactcatggt tatggcagca ctgcataatt ctcttactgt catgccatcc
8761 gtaagatgct tttctgtgac tggtgagtac tcaaccaagt cattctgaga atagtgtatg
8821 cggcgaccga gttgctcttg cccggcgtca atacgggata ataccgcgcc acatagcaga
8881 actttaaaag tgctcatcat tggaaaacgt tcttcggggc gaaaactctc aaggatctta
8941 ccgctgttga gatccagttc gatgtaaccc actcgtgcac ccaactgatc ttcagcatct
9001 tttactttca ccagcgtttc tgggtgagca aaaacaggaa ggcaaaatgc cgcaaaaaag
9061 ggaataaggg cgacacggaa atgttgaata ctcatactct tcctttttca atattattga
9121 agcatttatc agggttattg tctcatgagc ggatacatat ttgaatgtat ttagaaaaat
9181 aaacaaatag gggttccgcg cacatttccc cgaaaagtgc cac

**pPPR-mCherry_CAT**

*388-1441 1054 bp of 3′ PPR sequence*
*1442-2152 mCherry coding sequence*
*2157-2469 SAG1 terminator*
*2482-2935 GRA1 promoter*
*2937-3602 CAT (chloramphenicol acetyltransferase) coding sequence*
*3607-3919 SAG1 terminator*
*4128-4795 Col E1 origin*
*4943-5803 AMP*

```
   1 ctaaattgta agcgttaata ttttgttaaa attcgcgtta aatttttgtt aaatcagctc
  61 atttttaac caataggccg aaatcggcaa aatcccttat aaatcaaaag aatagaccga
 121 gatagggttg agtggccgct acagggcgct cccattcgcc attcaggctg cgcaactgtt
 181 gggaagggcg tttcggtgcg ggcctcttcg ctattacgcc agctggcgaa agggggatgt
 241 gctgcaaggc gattaagttg ggtaacgcca gggttttccc agtcacgacg ttgtaaaacg
 301 acggccagtg agcgcgacgt aatacgactc actatagggc gaattggcgg aaggccgtca
 361 aggcctaggc gcgccaggtc tcatgccgga gggaatgcag ccgaagagat cggccgatcc
 421 ggagggcgac aagaacgatc ctggttgtca aacagcagga aacgatcccg gggagagagac
 481 aggactgaga cgacgagaag gagagagaga cggagtgaat gaagaagcgg gaggtagaga
 541 gaaaggcgca tcagggcaga tagaacgaga gcgagaagga gaacacgata cagaagaagc
 601 aagagagaga ctcgaagaga aagaagaaga gagagaaaag gagaacagtg aacgggaagg
 661 gagagcaata agagcagttt caggagagag aggattgtcg gtttctgctt tcttgagttc
 721 cgtcctcggc ccgcgtcgcg cggtgcttga gagcatgcgg gaagagccgg ttttggagga
 781 cacggtgacc gaggaacctg agagccttga actgcatgca gacgcagaag cgcagctgga
 841 ggcgaagcag agggaaggcg agagccgcga cagaagcggt gaaaacacag aagcctcaac
 901 ggagaggaga aactcgccag accgagaaga agagaaacag gatcctgtca cgacggcaac
 961 ccaggagaca gatgcgaaca ctgggggaac gctgtcgcag gcgggaggaa gaaagagcga
1021 agcagcttcc agatccgaat ctgtcgagac gagtctggat cttcttcttc gactcaagca
1081 caagacgccg gcagagacgc agcagctcga agatggaccg cctcgctggc tcgccaggcg
1141 aagcggcaca ggggagccgg gagctgaggc cgtcgcaggt agccgccggg gtgtacatcc
1201 acccgacggc cgggcgggag tcggaatccc tggaggaggc gcaggctctc agaggcagag
1261 acaggagacg agagggcaga gtgcatttct ctttgcaggt gaccacggct ggggatggcc
1321 gccgcactgg gaagaaaaac tcgacaaggc tcgagccaga ttgcagcgaa cgaacgacga
1381 aagtggagaa accgaggaaa atgcaaagac agaaacccct tggggggatgc gaccgacttc
1441 gatggtgagc aagggcgagg aggataacat ggccatcatc aaggagttca tgcgcttcaa
1501 ggtgcacatg gagggctccg tgaacggcca cgagttcgag atcgagggcg agggcgaggg
1561 ccgcccctac gagggcaccc agaccgccaa gctgaaggtg accaagggtg gcccccctgcc
1621 cttcgcctgg gacatcctgt cccctcagtt catgtacggc tccaaggcct acgtgaagca
1681 ccccgccgac atccccgact acttgaagct gtccttcccc gagggcttca agtgggagcg
1741 cgtgatgaac ttcgaggacg gcggcgtggt gaccgtgacc caggactcct ccctgcagga
1801 cggcgagttc atctacaagg tgaagctgcg cggcaccaac ttcccctccg acggccccgt
1861 aatgcagaag aaaaccatgg gctgggaggc ctcctccgag cggatgtacc ccgaggacgg
1921 cgccctgaag ggcgagatca agcagaggct gaagctgaag gacggcggcc actacgacgc
1981 tgaggtcaag accacctaca aggccaagaa gcccgtgcag ctgcccggcg cctacaacgt
2041 caacatcaag ttggacatca cctcccacaa cgaggactac accatcgtgg aacagtacga
2101 acgcgccgag ggccgccact ccaccggcgg catggacgag ctgtacaagt gagcttatca
2161 ccgttgtgct cacttctcaa atcgacaaag gaaacacact tcgtgcagca tgtgccccat
2221 tataaagaaa ctgagttgtt ccgctgtggc ttgcaggtgt cacatccaca aaaaccggcc
2281 gactctaaat aggagtgttt cgcagcaagc agcgaaagtt tatgactggg tccgaatctc
2341 tgaacggatg tgtggcggac ctggctgatg ttgatcgccg tcgacacacg cgccacatgg
2401 gtcaatacac aagacagcta tcagttgttt tagtcgaacc ggttaacaca attcttgccc
2461 ccccgagggc gctgcaagga gatagcgtgg tactcgtcac gaataccaat cgctggggtc
2521 gcggcgggag gaatatgctg tttgttgacc atacgatcac gctgaacgaa aacatggtat
2581 gagacgccgt aagcgggcac aggttgtttg ccctcgtctc attgcggacc aattcccggt
2641 ccaccgctgc gtctcgactc gacggttgtg accaccccac ttcgcattgg gcagtcggta
2701 aagccacaac attactttgc aattttatcg gttgaaactg ccgagcgagc ttgcgttttt
```

2761 gggtgctatc ttctcccacc ttttatcagt taagttgtac agtgagtgtc agcttgtttc
2821 gacacgtctg tatagacgca actcggtttg cttgtgttgt ttggtggctg gccaaatcaa
2881 aggctattca tttttcactt gctgttgttc tttgaagaaa tcaagcaaga gtcaaaatgc
2941 tgcatgagaa aaaaatcact ggatatacca ccgttgatat atcccaatcg catcgtaaag
3001 aacattttga ggcatttcag tcagttgctc aatgtaccta taaccagacc gttcagctgg
3061 atattacggc ctttttaaag accgtaaaga aaaataagca caagttttat ccggccttta
3121 ttcacattct tgcccgcctg atgaatgctc atccggaatt ccgtatggca atgaaagacg
3181 gtgagctggt gatatgggat agtgttcacc cttgttacac cgttttccat gagcaaactg
3241 aaacgttttc atcgctctgg agtgaatacc acgacgattt ccggcagttt ctacacatat
3301 attcgcaaga tgtggcgtgt tacggtgaaa acctggccta tttccctaaa gggtttattg
3361 agaatatgtt tttcgtctca gccaatccct gggtgagttt caccagtttt gatttaaacg
3421 tggccaatat ggacaacttc ttcgcccccg ttttcaccat gggcaaatat tatacgcaag
3481 gcgacaaggt gctgatgccg ctggcgattc aggttcatca tgccgtctgt gatggcttcc
3541 atgtcggcag aatgcttaat gaattacaac agtactgcga tgagtggcag ggcggggctt
3601 aagcttatca ccgttgtgct cacttctcaa atcgacaaag gaaacacact tcgtgcagca
3661 tgtgccccat tataaagaaa ctgagttgtt ccgctgtggc ttgcaggtgt cacatccaca
3721 aaaaccggcc gactctaaat aggagtgttt cgcagcaagc agcgaaagtt tatgactggg
3781 tccgaatctc tgaacggatg tgtggcggac ctggctgatg ttgatcgccg tcgacacacg
3841 cgccacatgg gtcaatacac aagacagcta tcagttgttt tagtcgaacc ggttaacaca
3901 attcttgccc ccccgagggc gctactagag gatgcacatg tgaccgaggg aattaattaa
3961 ctggcctcat gggccttccg ctcactgccc gctttccagt cgggaaacct gtcgtgccag
4021 ctgcattaac atggtcatag ctgtttcctt gcgtattggg cgctctccgc ttcctcgctc
4081 actgactcgc tgcgctcggt cgttcgggta aagcctgggg tgcctaatga gcaaaaggcc
4141 agcaaaaggc caggaaccgt aaaaaggccg cgttgctggc gtttttccat aggctccgcc
4201 cccctgacga gcatcacaaa aatcgacgct caagtcagag gtggcgaaac ccgacaggac
4261 tataaagata ccaggcgttt ccccctggaa gctccctcgt gcgctctcct gttccgaccc
4321 tgccgcttac cggatacctg tccgcctttc tcccttcggg aagcgtggcg ctttctcata
4381 gctcacgctg taggtatctc agttcggtgt aggtcgttcg ctccaagctg ggctgtgtgc
4441 acgaaccccc cgttcagccc gaccgctgcg ccttatccgg taactatcgt cttgagtcca
4501 acccggtaag acacgactta tcgccactgg cagcagccac tggtaacagg attagcagag
4561 cgaggtatgt aggcggtgct acagagttct tgaagtggtg gcctaactac ggctacacta
4621 gaagaacagt atttggtatc tgcgctctgc tgaagccagt taccttcgga aaaagagttg
4681 gtagctcttg atccggcaaa caaaccaccg ctggtagcgg tggttttttt gtttgcaagc
4741 agcagattac gcgcagaaaa aaaggatctc aagaagatcc tttgatcttt tctacggggt
4801 ctgacgctca gtggaacgaa aactcacgtt aagggatttt ggtcatgaga ttatcaaaaa
4861 ggatcttcac ctagatcctt ttaaattaaa aatgaagttt taaatcaatc taaagtatat
4921 atgagtaaac ttggtctgac agttaccaat gcttaatcag tgaggcacct atctcagcga
4981 tctgtctatt tcgttcatcc atagttgcct gactccccgt cgtgtagata actacgatac
5041 gggagggctt accatctggc cccagtgctg caatgatacc gcgagaacca cgctcaccgg
5101 ctccagattt atcagcaata aaccagccag ccggaagggc cgagcgcaga agtggtcctg
5161 caactttatc cgcctccatc cagtctatta attgttgccg ggaagctaga gtaagtagtt
5221 cgccagttaa tagtttgcgc aacgttgttg ccattgctac aggcatcgtg gtgtcacgct
5281 cgtcgtttgg tatggcttca ttcagctccg gttcccaacg atcaaggcga gttacatgat
5341 cccccatgtt gtgcaaaaaa gcggttagct ccttcggtcc tccgatcgtt gtcagaagta
5401 agttggccgc agtgttatca ctcatggtta tggcagcact gcataattct cttactgtca
5461 tgccatccgt aagatgcttt tctgtgactg gtgagtactc aaccaagtca ttctgagaat
5521 agtgtatgcg gcgaccgagt tgctcttgcc cggcgtcaat acgggataat accgcgccac
5581 atagcagaac tttaaaagtg ctcatcattg gaaaacgttc ttcggggcga aaactctcaa
5641 ggatcttacc gctgttgaga tccagttcga tgtaacccac tcgtgcaccc aactgatctt
5701 cagcatcttt tactttcacc agcgtttctg ggtgagcaaa aacaggaagg caaaatgccg
5761 caaaaaaggg aataagggcg acacggaaat gttgaatact catactcttc ctttttcaat
5821 attattgaag catttatcag ggttattgtc tcatgagcgg atacatattt gaatgtattt
5881 agaaaaataa acaaataggg gttccgcgca catttccccg aaaagtgcca c
//
